# Pancreatic β-Cell Dysfunction in Diet-Induced Obese Mice: Roles of AMP-Kinase, Protein Kinase Cε, Mitochondrial and Cholesterol Metabolism, and Alterations in Gene Expression

**DOI:** 10.1371/journal.pone.0153017

**Published:** 2016-04-04

**Authors:** Émilie Pepin, Anfal Al-Mass, Camille Attané, Kezhuo Zhang, Julien Lamontagne, Roxane Lussier, S. R. Murthy Madiraju, Erik Joly, Neil B. Ruderman, Robert Sladek, Marc Prentki, Marie-Line Peyot

**Affiliations:** 1 Montreal Diabetes Research Center and Centre de Recherche du CHUM, Montréal, Québec, Canada; 2 Departments of Medicine and Human Genetics, McGill University, Montreal, Québec, Canada; 3 Departments of Nutrition, Biochemistry and Molecular Medicine, Faculty of Medicine, University of Montréal, Montreal, Québec, Canada; 4 Departments of Medicine and Physiology and Biophysics, Boston University School of Medicine and Diabetes Unit, Boston Medical Center, Boston, MA, United States of America; Universidad Miguel Hernández de Elche, SPAIN

## Abstract

Diet induced obese (DIO) mice can be stratified according to their weight gain in response to high fat diet as low responders (LDR) and high responders (HDR). This allows the study of β-cell failure and the transitions to prediabetes (LDR) and early diabetes (HDR). C57BL/6N mice were fed for 8 weeks with a normal chow diet (ND) or a high fat diet and stratified as LDR and HDR. Freshly isolated islets from ND, LDR and HDR mice were studied *ex-vivo* for mitochondrial metabolism, AMPK activity and signalling, the expression and activity of key enzymes of energy metabolism, cholesterol synthesis, and mRNA profiling. Severely compromised glucose-induced insulin secretion in HDR islets, as compared to ND and LDR islets, was associated with suppressed AMP-kinase activity. HDR islets also showed reduced acetyl-CoA carboxylase activity and enhanced activity of 3-hydroxy-3-methylglutaryl-CoA reductase, which led respectively to elevated fatty acid oxidation and increased cholesterol biosynthesis. HDR islets also displayed mitochondrial membrane hyperpolarization and reduced ATP turnover in the presence of elevated glucose. Expression of protein kinase Cε, which reduces both lipolysis and production of signals for insulin secretion, was elevated in DIO islets. Genes whose expression increased or decreased by more than 1.2-fold were minor between LDR and ND islets (17 differentially expressed), but were prominent between HDR and ND islets (1508 differentially expressed). In HDR islets, particularly affected genes were related to cell cycle and proliferation, AMPK signaling, mitochondrial metabolism and cholesterol metabolism. In conclusion, chronically reduced AMPK activity, mitochondrial dysfunction, elevated cholesterol biosynthesis in islets, and substantial alterations in gene expression accompany β-cell failure in HDR islets. The β-cell compensation process in the prediabetic state (LDR) is largely independent of transcriptional adaptive changes, whereas the transition to early diabetes (HDR) is associated with major alterations in gene expression.

## Introduction

Obesity when associated with dyslipidemia, hyperinsulinemia, and insulin resistance, increases the risk of developing type 2 diabetes (T2D), cardiovascular disease and certain cancers [1]. Obesity-associated T2D is generally thought to result from the inability of pancreatic islets to secrete sufficient insulin to compensate for the increased insulin resistance of peripheral tissues [2,3]. Alternatively, the initial hyperinsulinemia may drive obesity, and insulin resistance and T2D follow as a result of β-cell failure via its exhaustion and other mechanisms [4–6].

Several studies have shown that β-cell mitochondrial and lipid metabolism [7–9] as well as AMP-activated protein kinase (AMPK) [10]; and protein kinase Cε (PKCε) [11] signaling play major roles in the regulation of insulin secretion. Fatty acids augment glucose-stimulated insulin secretion (GSIS) by β-cells via the receptor FFAR1[12] and the generation of metabolic coupling factors [13], in particular 1-monoacylglycerol [14], generated by the lipolysis arm of the glycerolipid/free fatty acid (GL/FFA) cycle [15]. However, chronic exposure of the β-cell to elevated FFA causes metabolic stress and curtails the GSIS response via glucolipotoxicity [5,16].

High fat diet-induced obese (DIO) mice gradually develop hyperglycemia [17] and defective GSIS [18–20]; they represent a model of mild diabetes resembling human T2D. We recently showed that C57BL/6N DIO mice, that do not harbor a mutation in the nicotinamide nucleotide transhydrogenase gene [21], display heterogeneity in their weight gain response and can be stratified as low HFD responders (LDR) and high HFD responders (HDR) [22]. LDR mice have a moderate phenotype with obesity, β-cell compensation for insulin resistance and mild β-cell dysfunction. Essentially, they show characteristics of ‘pre-diabetes’ in humans. HDR mice in turn have the characteristics of ‘early-diabetes’ with more severe β-cell dysfunction. Both LDR and HDR DIO mice exhibit reduced GSIS and altered free fatty acid (FFA) metabolism with HDR mouse islets showing more defective secretion in association with elevated β-oxidation of FFA and free cholesterol accumulation in β-cells [22]. Thus, a comparison of the responses of these stratified DIO mice with the same genetic background offers a unique opportunity to enhance our understanding of the mechanisms involved in both the development of obesity-linked β-cell dysfunction, and the transition from pre-diabetes to early diabetes.

The present study provides evidence that altered β-cell AMPK and PKCε signaling, mitochondrial dysfunction, enhanced cholesterol synthesis and deposition, and major changes in gene expression contribute to β-cell failure in the most diet responsive HDR mice.

## Materials and Methods

### Materials

Glucose-free RPMI 1640 media was purchased from Gibco (Burlington, ON, Canada). Fatty-acid free BSA and all chemicals, unless otherwise specified, were purchased from Sigma-Aldrich (St-Louis, MO, USA). Rhodamine 123 was obtained from Molecular Probes (Burlington, ON, Canada). Cell culture supplies were purchased from Corning (New York, USA). Antibodies for Western blotting were purchased from Cell Signaling Technology (Beverly, MA, USA) except SIRT1, phosphor-Ser^79^ ACC and phospho-Ser^872^ HMG-CoA reductase (Millipore, Billerica, MA, USA), PKCε, phosphor-Ser^729^ PKCε and LKB1 (Santa Cruz Biotechnology, Santa Cruz, CA, USA) and tubulin (Abcam, Cambridge, MA, USA). Protein concentration was determined using a BCA kit (Pierce, Rockford, IL, USA).

### Animals and diets

Five-week old male C57BL/6N mice with a pure genetic background were purchased from Charles River Laboratories (Raleigh, NC, USA). They were housed two per cage, on a 12h light/dark cycle at 21°C with free access to water and a standard diet (Teklad Global 18% protein rodent diet, 15% fat by energy, Harland Teklad, Madison, WI, USA). After one week of acclimatization, the mice were fed either the standard normal diet (ND) or high fat diet (HFD) for 8 weeks (Bioserv F-3282, 60% energy from fat, Frenchtown, NJ, USA). Body weight was measured weekly and at 7.5 weeks, blood glucose was measured in fed mice with a portable glucometer (Contour, Bayer, Toronto, ON, Canada). After 8 weeks, HFD fed mice were stratified into LDR and HDR groups according to their body weight gain, and plasma parameters (insulin, cholesterol, non-esterified free fatty acid and triglycerides) were determined in fed mice as described previously [22]. None of the animals involved in this study showed sign of illness. At the end of the HFD protocol, all mice were anesthetized with intraperitoneal injection of ketamine/xylazine. Anesthesia was confirmed by lack of responsiveness to toe pinching and all efforts were made to minimize animal suffering. Animals were sacrificed by cervical dislocation and blood was collected by cardiac puncture followed by removal of pancreas. All procedures were approved by the Institutional Committee for the Protection of Animals at the Centre de Recherche du Centre Hospitalier de l’Université de Montréal and were done in accordance with the Canadian Council of Animal Care guidelines.

### Islet isolation and culture

Islets from ND, LDR and HDR mice were isolated as described before [23] with the exception that Liberase TL (Roche Diagnostic, Laval, QC, Canada) was used instead of collagenase XI for pancreatic tissue digestion. After isolation, islets were allowed to recover for 2h at 37°C in RPMI complete medium containing 3 mM glucose and supplemented with 10% (w/v) fetal bovine serum (FBS) [23].

### Insulin secretion

After recovery, islets were distributed in 12-well plates at a density of 10 islets per well and preincubated for 45 min at 37°C in Krebs-Ringer bicarbonate buffer containing 10 mM HEPES (pH 7.4) (KRBH), 0.5% defatted bovine serum albumin (d-BSA), and 3 mM glucose. They were then incubated for 1 h in KRBH/0.5% d-BSA at 3, 8, and 16 mM glucose or 3 mM glucose with 35 mM KCl. After 1h, media were kept for insulin measurements by radioimmunoassay. Total islet insulin content was measured by radioimmunoassay [23] following acid—ethanol (0.2 mM HCl in 75% ethanol) extraction.

### Mitochondrial membrane potential

Isolated islets were dispersed by trypsinization and cultured overnight on glass coverslips coated with poly-lysine (Sigma-Aldrich, Oakville, ON, Canada) in RPMI complete medium containing 5.5 mM glucose and supplemented with 10% FBS. Islet cells were starved for 2h in RPMI complete medium with 3 mM glucose and then loaded for 25 min with 10μg/mL Rhodamine-123 in KRBH supplemented with 2.8 mM glucose and 0.5% BSA. Fluorescence intensity was measured by confocal microscopy following incubations at 3 mM and 16 mM glucose, and at 3 mM glucose in the presence of 25 μM carbonyl cyanide 4-(trifluoromethoxy) phenylhydrazone (FCCP) [24].

### Islet oxygen consumption

Batches of 75 hand-picked islets were placed in to a 24-well Seahorse islet plate. Islets were preincubated for 40 min at 37°C without CO_2_ in DMEM supplemented with 2 mM L-glutamine, 1 mM sodium pyruvate, 1% FBS and 3 mM glucose before loading in to the XF24 analyzer (Seahorse Bioscience, Billerica, MA, USA). Oxygen consumption rate (OCR) was measured for 20 min at 3 mM glucose to assess basal respiration, and after glucose levels were elevated to 16 mM for 50 min to determine glucose-induced O_2_ consumption. Finally, different inhibitors of the respiratory chain or uncouplers were added by three successive injections to assess uncoupled respiration, maximal respiration and non-mitochondrial respiration, respectively: oligomycin (F1F0-ATP synthase inhibitor; 5 μM); FCCP (uncoupler; 1 μM) and rotenone plus antimycin A (complex I and III inhibitors, both at 5 μM). ATP turnover at 16 mM glucose (OCR 16 mM glucose—OCR oligomycin), uncoupled respiration (OCR FCCP—OCR oligomycin), and maximal respiration (OCR FCCP—OCR rotenone plus antimycin) were calculated [25].

### Immunoblotting

To determine the expression of specific proteins, isolated islets were washed three times with PBS, resuspended in lysis buffer (50 mM HEPES pH 7.5, 1% NP40, 2 mM sodium orthovanadate, 4 mM EDTA, 10 μg/mL aprotinin, 1 μg/mL leupeptin, 100 mM sodium fluoride, 10 mM sodium pyrophosphate, 1 mM phenylmethanesulfonylfluoride) and snap-frozen in liquid nitrogen till use. For the determinations of various phosphoproteins, isolated islets were preincubated for 1h in KRBH containing 0.5% d-BSA and 3 mM glucose, and subsequently incubated for 30 min at 3 or 16 mM glucose. Islets were then washed three times with PBS, resuspended in lysis buffer and snap-frozen in liquid nitrogen. Total protein extracts (15 μg) from ND, LDR and HDR islets were separated on 4–15% gradient SDS-PAGE (Bio-Rad, Missisauga, ON, Canada) and transferred to PVDF-Immobilon membranes (Millipore, Billerica, MA, USA). Membranes were probed with antibodies against total-AMPKα (1/500 in 5% milk, #2532), phospho-Thr^172^ AMPKα (1/1000 in 5% BSA, #2535), total-acetyl-CoA carboxylase (ACC, 1/500 in 5% milk, #3662), phospho-Ser^79^ ACC (1/1000 in 5% milk, #07–303), SIRT1 (1/1000 in 5% milk, #09–844), phospho-Ser^872^ HMG-CoA reductase (1/400 in 5% milk, #09–356), total-PKCε (1/500 in 5% BSA, #sc-214), phospho-Ser^729^ PKCε (1/250 in 5% BSA, #sc-12355) and total-LKB1 (1/500 in 5% BSA, #sc32245). Alpha tubulin (1/10000 in 5% milk, #ab4074) was used as a loading control. Protein bands were detected using an HRP-labeled anti-rabbit IgG (Bio-Rad, Mississauga, ON, Canada) and a SuperSignal West Pico chemiluminescent kit (Thermo Scientific, Rockford, IL, USA). Protein bands were quantified using Scion Image (Frederick, MD, USA).

### Total cholesterol content

After 2h recovery in RPMI complete medium supplemented with 1% FBS at 3 mM glucose, batches of 100 islets from ND, LDR and HDR mice were distributed in 6-well plates and incubated for 3h at 37°C in RPMI 1% FBS and 3 mM or 16 mM glucose. At the end of the incubation, total islet cholesterol (free cholesterol plus cholesterol esters) was measured [22].

### Transcriptomic Profiling

Total RNA was extracted from 300 islets for 8 separate mice from each group (ND, LDR, HDR: 8 islet isolations per group), using the RNeasy Mini kit (Qiagen, Valencia, CA, USA) following the manufacturer’s protocol. The quality of the total RNA was evaluated on an Agilent 2100 Bioanalyzer system (Agilent, Palo Alto, CA, USA). Microarray analysis was performed on total RNA using the GeneChip Mouse Gene 1.0 ST microarrays (eight arrays per group; Affymetrix, Santa Clara, CA, USA). One hundred nanograms of total RNA were processed using the Ambion WT Expression Kit (Invitrogen). The resulting fragmented and labeled single-stranded cDNA was processed according to the Affymetrix protocol. The Partek (St. Louis, MO, USA) Genomics Suite was used for data analysis. The data were normalized by Robust Multichip Average (RMA) algorithm, which uses background adjustment, quantile normalization, and summarization. After correction of statistical significance for multiple comparisons using false discovery rate; the transcripts that showed significant differences in expression between the HDR, LDR and ND groups (i.e. whose expression increased or decreased by more than 1.2 fold change, *p* <0.05) were classified using pathway enrichment analysis using Ingenuity Pathways Analysis (IPA; Ingenuity Systems, Redwood City, CA, USA).

### Quantitative Real time-PCR

Total RNA was extracted from islets as described above. Gene expression was determined by a standard curve method and normalized to the expression of beta-actin. Real-time PCR analysis was performed on Rotor-Gene R3000 (Corbett Research, Mortlake, NSW, Australia) using Quantitech Sybrgreen (Qiagen, Mississauga, ON, Canada) according to the manufacturer’s instructions. Primers, listed in S1 Table, were designed using Primer3 software. Results are expressed as the ratio of target mRNA to beta-actin mRNA.

### Statistical analysis

Data are expressed as mean ± SEM. Statistical significance was calculated by ANOVA with Tukey or Bonferroni post-hoc testing, as indicated, using GraphPad Prism 6.0. A *p* value of <0.05 was considered significant.

## Results

### Impaired insulin secretion and mitochondrial dysfunction in DIO islets

**Fig 1:**
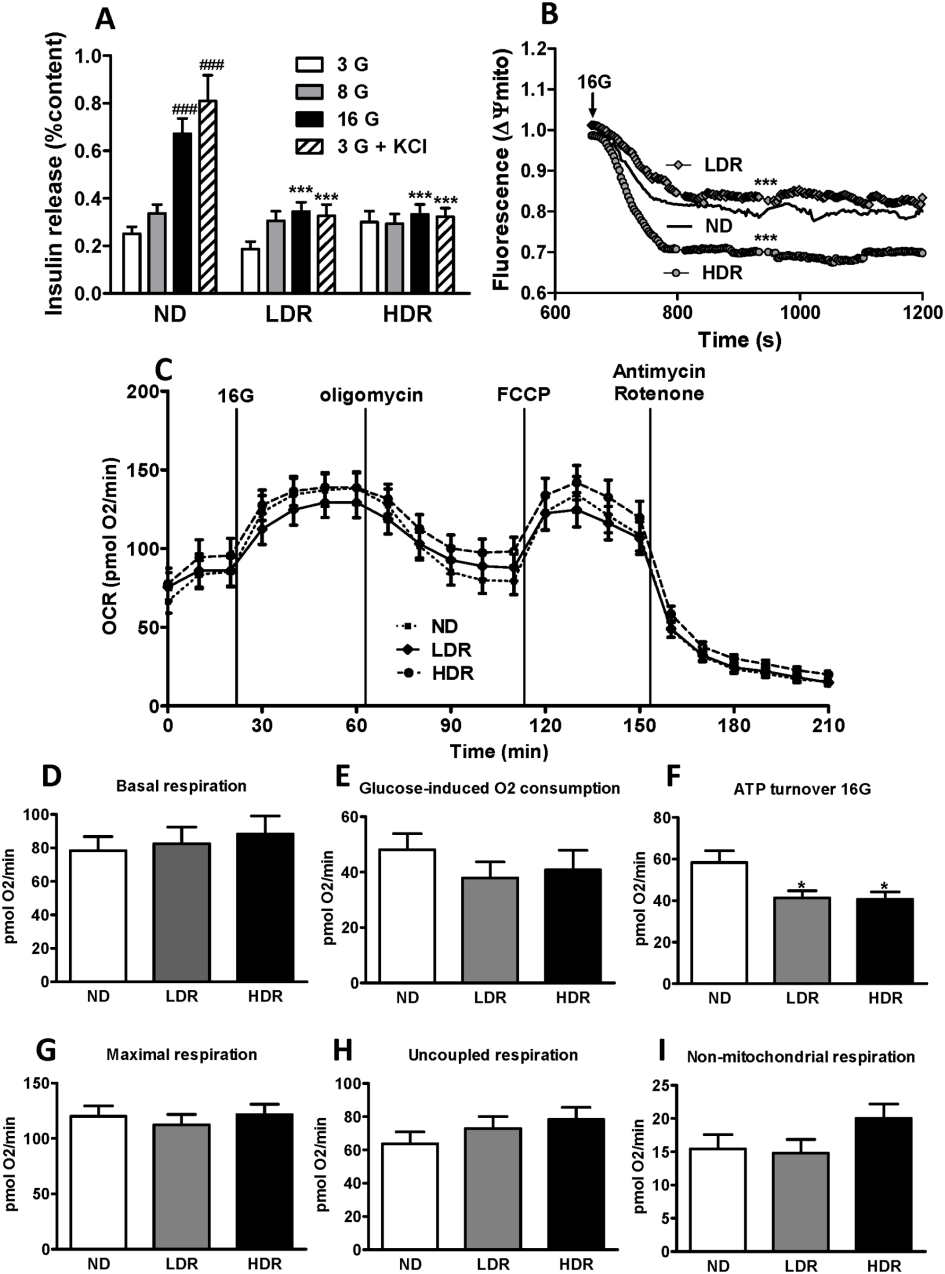
Defective insulin secretion and mitochondrial dysfunction in DIO islets. (A) Insulin secretion was measured in freshly isolated islets from normal diet (ND), and obese high fat diet fed low responders (LDR) and high responders (HDR) mice. Groups of 10 islets were incubated 1 h in KRBH at 3, 8, or 16 mM glucose (G) or 3 mM glucose ± 35 mM KCl. Means ± SEM of 10–12 determinations from islets of 6 animals per group in three separate experiments. ***p<0.001 versus ND for the same glucose concentration; ###p<0.001 versus 3 mM glucose; one-way ANOVA, Tukey post-hoc test. (B) Mitochondrial membrane potential (Δψmito) measured by Rhodamine123 fluorescence in dispersed islet cells from ND, LDR and HDR mice. Δψmito was initially measured at 3 mM glucose to set a baseline and then at 16 mM glucose. Data were normalized to baseline fluorescence. Means of 6 (ND) or 5 (LDR and HDR) mice. ***p<0.0001 vs ND; One-way ANOVA, repeated measures, Tukey post-hoc test. (C) Mitochondrial O_2_ consumption rate (OCR) measured at 3 mM glucose and then at 16 mM glucose (16G). (D) Baseline respiration at 3 mM glucose, (E) glucose-induced respiration as the difference in OCR between 16 and 3 mM glucose, (F) ATP-turnover at 16G, (G) maximal respiration, (H) uncoupled respiration and (I) non-mitochondrial respiration were determined using mitochondrial inhibitors. Means ± SEM of 5 mice per group, each with quadruplicate observations. *p<0.05 versus ND; One-way ANOVA, Tukey post-hoc test.

Insulin secretion was measured in freshly isolated islets from ND, LDR and HDR mice. Increasing the ambient glucose concentration from 3 to 16 mM stimulated insulin secretion in control ND islets by 3 fold, a response that was reduced in LDR islets and almost completely abolished in HDR islets (Fig 1A), as previously observed [22]. A very similar pattern of events was observed when KCl-induced insulin secretion was studied (Fig 1A).

Mitochondria in the β-cell generate signals for insulin secretion and their dysfunction is associated with reduced GSIS and the development of T2D [26,27]. We assessed whether mitochondrial function is defective in islets of mice with DIO. Mitochondrial membrane potential (Δψ_mito_) measurements in HDR islets showed hyperpolarization in response to 16 mM glucose while Δψ_mito_ was similar in LDR and ND islets (Fig 1B). We also examined mitochondrial respiration (Fig 1C). There was no change in basal (Fig 1D), maximal (Fig 1G), uncoupled (Fig 1H) and non-mitochondrial respiration (Fig 1I) in DIO islets compared to ND islets. Glucose-induced O_2_ consumption was reduced in LDR and HDR islets without reaching statistical significance (Fig 1E), and glucose-induced ATP turnover was decreased to the same extent in both LDR and HDR islets (Fig 1F). Lack of change in the oxidation of ^14^C-glucose to ^14^CO_2_ in LDR and HDR islets was noticed in our earlier study [22]. Thus, some mitochondrial dysfunction is apparent in both LDR and HDR islets but the alteration is more prominent in the HDR group which exhibited mitochondrial hyperpolarization at high glucose.

### Altered AMPK activity and enhanced cholesterol synthesis in HDR islets

The fuel sensor AMPK plays a central role in energy homeostasis and chronic alterations in its activity have been implicated in diseases associated with the metabolic syndrome [28]. AMPK activation caused by increases in AMP and ADP and upstream kinases [29], is associated with elevated fatty acid oxidation and decreased cholesterol biosynthesis due to the inactivation of acetyl CoA carboxylase (ACC) [30] and HMG-CoA reductase (HMGCR) [31], respectively. Glucose acutely reduces AMPK activity in the β-cell and increased AMPK activity in isolated islets has been shown to decrease GSIS [32]. The chronic effects of AMPK alterations are less understood.

**Fig 2:**
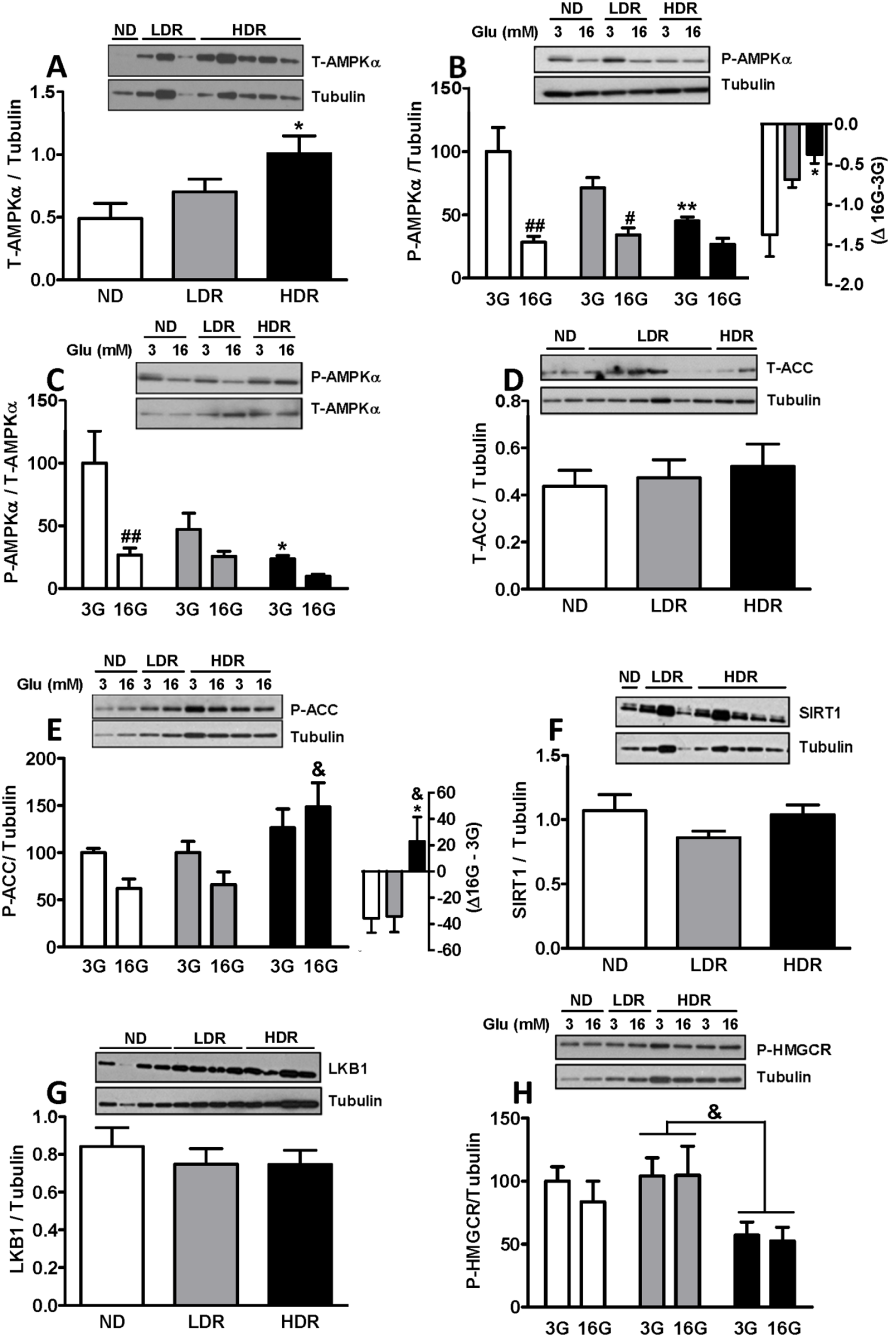
Altered activities of AMP-Kinase, acetyl-CoA carboxylase and HMG-CoA reductase in HDR islets. (A) Total AMPKα (n = 16–23); (B,C) phospho-AMPKα (P-Thr172 n = 6–14); (D) Total ACC (n = 12–18); (E) phospho-ACC (P-Ser79) (n = 7–9); (F) Total SIRT1 (n = 19–22); (G) Total LKB1 (n = 6–11); and (H) phospho-HMGCR (P-Ser872) (n = 6–12). Tubulin or total AMPK (T-AMPK) were used for normalization. Graphs represent means ± SEM for the number of determinations per experimental conditions in parenthesis. (A,D,F,G) non-treated islets. (B,C,E,H) islets treated at 3 mM (3G) or 16 mM (16G) glucose for 30 min and data are expressed as % of ND islets at 3 mM glucose (3G). # p<0.05, ##p<0.01 vs 3G for the same islet group; * p<0.05, ** p<0.01 vs ND for the same glucose concentration; & p<0.05 for HDR vs LDR of the same glucose concentration, One-way Anova, Tukey post-hoc test. ND: white; LDR: gray; HDR: black.

**Fig 3:**
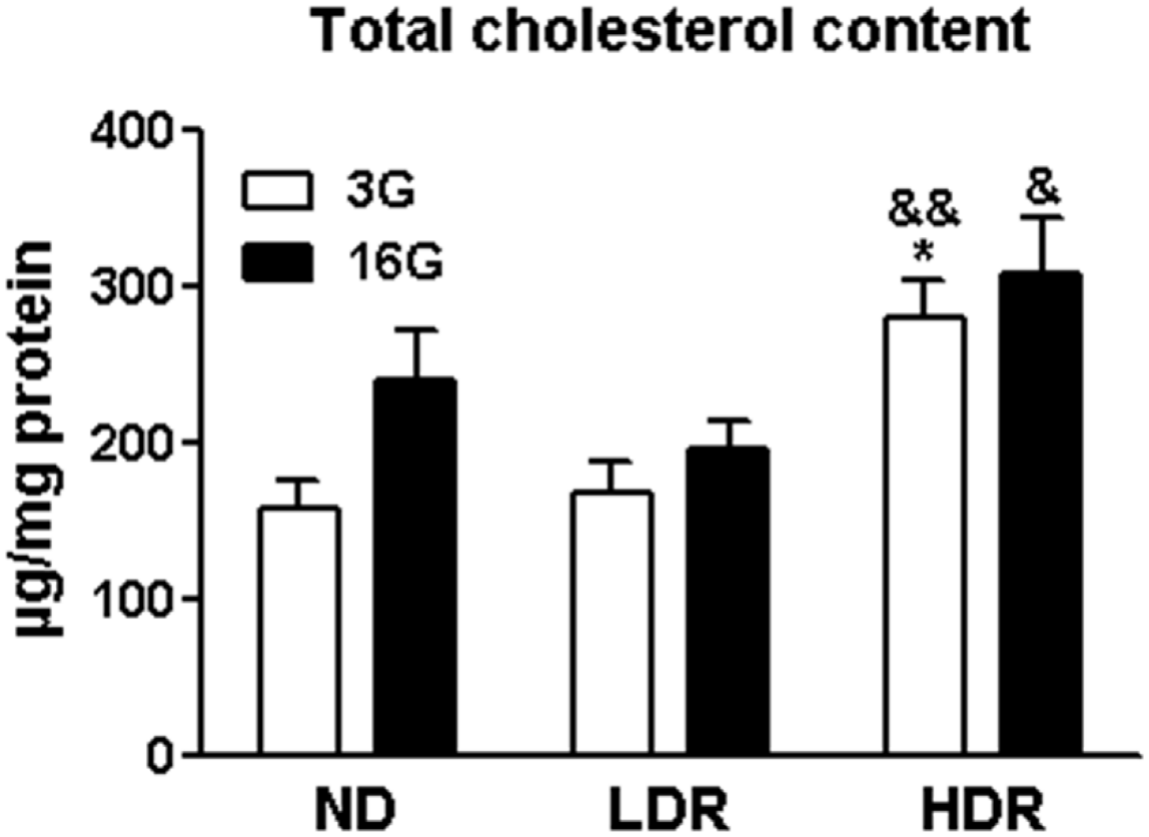
Increased total cholesterol content in HDR islets. Total cholesterol content of islets incubated at 3 mM (3G) or 16 mM (16G) glucose for 3h. Means ± SEM from 6 (ND and HDR) and 5 (LDR) mice. *p<0.05 vs ND, &p<0.05, &&p<0.01 for HDR vs LDR, for the same glucose concentration. One-way ANOVA, Tukey post-hoc test.

We noticed an increase in total AMPKα abundance in DIO islets, which was significant in the HDR group (Fig 2A). Activation of AMPK, as indicated by phospho-AMPK levels, was as expected decreased in ND, LDR and HDR islets incubated with elevated glucose (Fig 2B). Interestingly, phospho-AMPK levels, expressed as a function of either tubulin or total AMPK, greatly decreased in DIO islets particularly under basal conditions (3 mM glucose) in comparison to ND islets and this decrease was more pronounced in HDR islets (Fig 2B and 2C). We then examined whether the altered phospho-AMPK levels in DIO islets impacted AMPK’s downstream targets. There were no changes in total protein levels of the AMPK target ACC or of its phosphorylation in LDR islets, but a slight increase in phospho-ACC levels was noticed in HDR islets (Fig 2D and 2E). Even though phospho-ACC levels in response to high glucose treatment islets decreased in ND and LDR islets, presumably due to changes in phospho-AMPK, such was not the case in HDR islets (Fig 2E). Moreover, when expressed as the difference between 16 and 3 mM glucose, ACC phosphorylation was increased in HDR islets compared to both the ND and LDR groups (Fig 2E, inset). There was no change in total levels of SIRT1 (Fig 2F), a putative downstream target of AMPK or in its upstream kinase LKB1 (Fig 2G). Phosphorylation of HMGCR, which inactivates this enzyme, was similar at both low and high glucose in LDR islets as compared to ND islets; however, this was decreased by nearly 50% in HDR islets (Fig 2H), indicating that the activity of HMGCR which catalyzes the rate limiting step in the biogenesis of cholesterol was higher in HDR islets. This change in HMGCR phosphorylation status was reflected in total islet cholesterol levels, which were also not responsive to elevated glucose concentration in HDR vs ND islets, and showed an increase in HDR islets relative to ND and LDR islets (Fig 3).

Overall, in DIO conditions and particularly in the HDR group, glucose regulation of AMPK activity in the islets is completely obliterated and this impacts the AMPK targets ACC (reduced activity) with a resulting increase in fat oxidation [22], and HMGCR (enhanced activity) in association which decreases cholesterol synthesis and deposition.

### Increased PKCε expression in DIO islets

**Fig 4:**
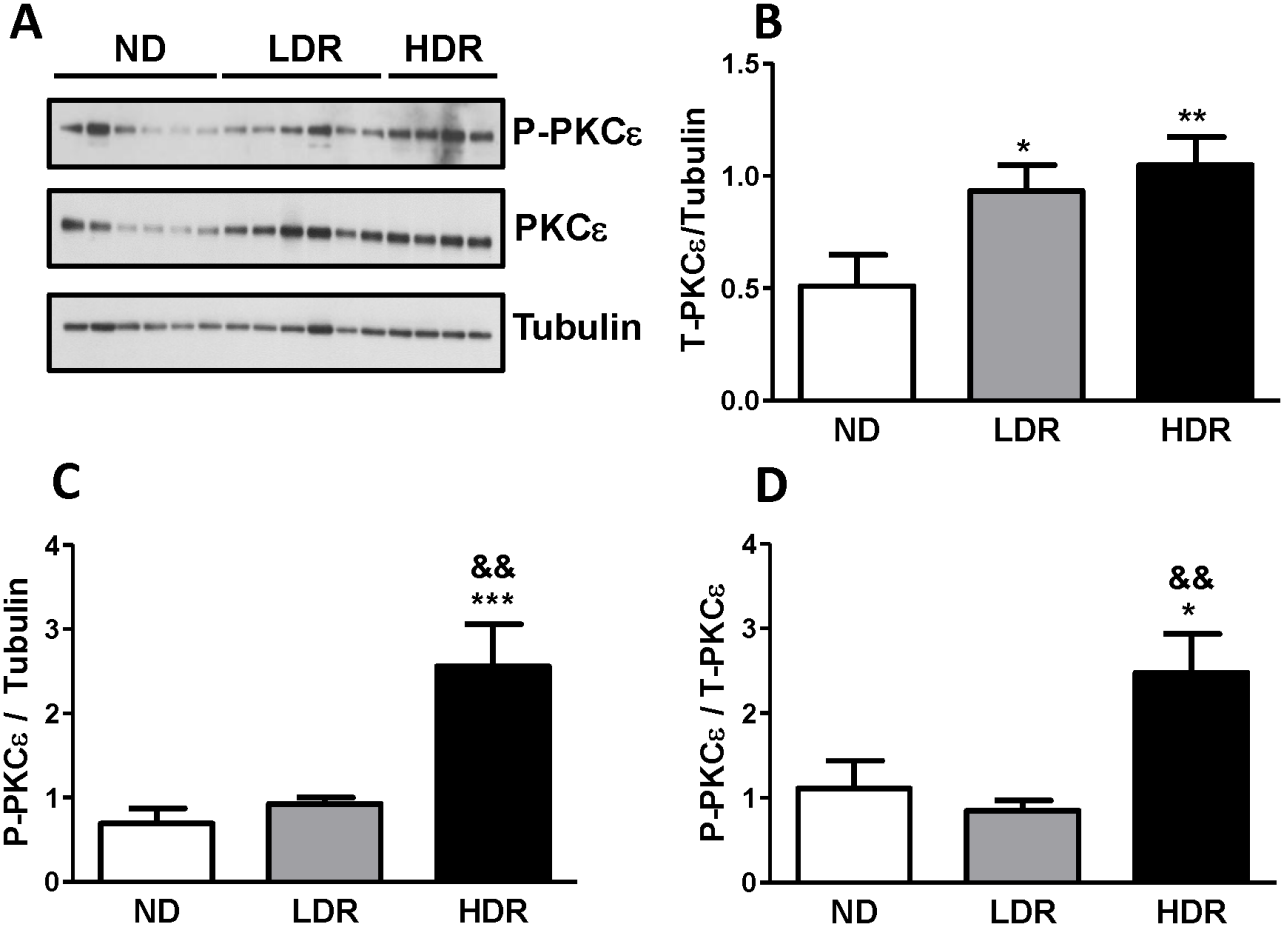
Increased total PKCε levels in DIO islets. Proteins from ND, LDR and HDR islets were probed with an antibody against total PKCε (T-PKCε) and phospho-Ser^729^ PKCε (P-PKCε). Tubulin or total PKCε were used for normalization. (A) Representative western blot of T-PKCε and P-PKCε, (B) expression level of T-PKCε normalized by tubulin, (C) P-PKCε level normalized by tubulin and (D) by total PKCε. Means ± SEM of 8 (ND), 13 (LDR) and 10 (HDR) mice. *p<0.05, **p<0.01 vs ND, One-way ANOVA, Tukey post-hoc test.

PKCε is a negative regulator of insulin secretion and lipolysis [11]. As in the present work we noticed decreased GSIS in both LDR and HDR islets and in an earlier study that decreased GSIS in DIO islets correlated with lipolysis [22], we examined the expression of PKCε in the DIO islets. As shown in Fig 4A and 4B, PKCε protein levels were increased more than two fold in LDR and HDR islets in comparison with ND islets. However, phospho-PKCε was only increased in HDR islets (Fig 4A, 4C and 4D).

### Altered gene expression in the HDR versus LDR and ND islets

**Fig 5:**
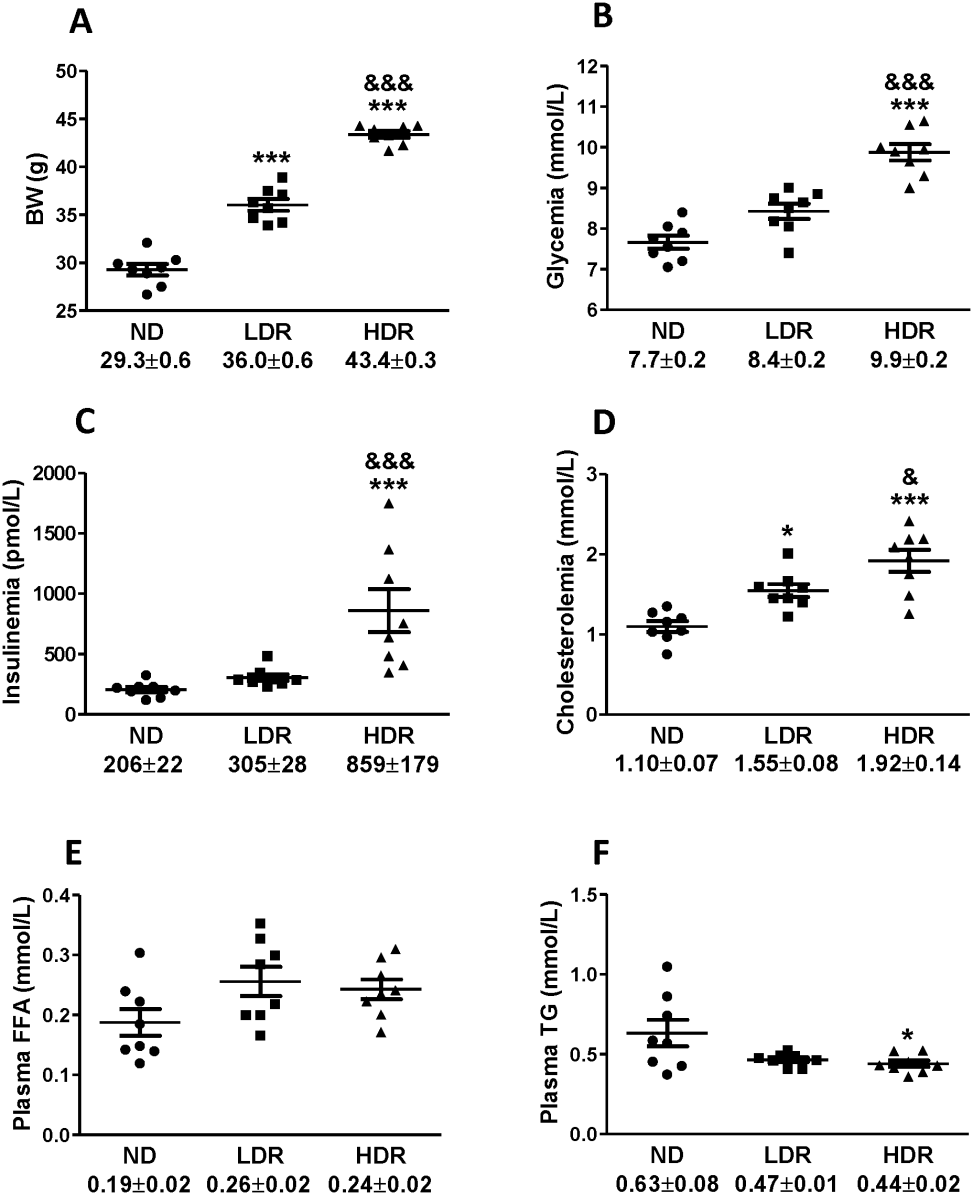
Individual metabolic parameters of C57BL/6N mice fed with a normal or HFD for 8 weeks used for islet gene expression analysis. (A) Body weight (BW), (B) glycemia, (C) insulinemia, (D) cholesterolemia, (E) plasma fatty acids and (F) plasma triglycerides. Means ± SEM of 8 animals per group are indicated below the X- axis for each graph. LDR or HDR versus ND: *P<0.05, ***P<0.001; HDR versus LDR: & P<0.05, && P<0.01, &&& P<0.001. One-way ANOVA-Bonferroni’s multiple comparison post hoc test.

We performed a microarray-based transcriptomic analysis on eight islet preparations from the ND, LDR and HDR groups. Body weight and blood parameters of the individual mice used for the microarray assay are shown in Fig 5. Increased plasma levels of glucose, insulin and cholesterol were prominent in the HDR group whereas the same parameters, although slightly elevated in LDR, were close to the values of the control ND animals.

**Fig 6:**
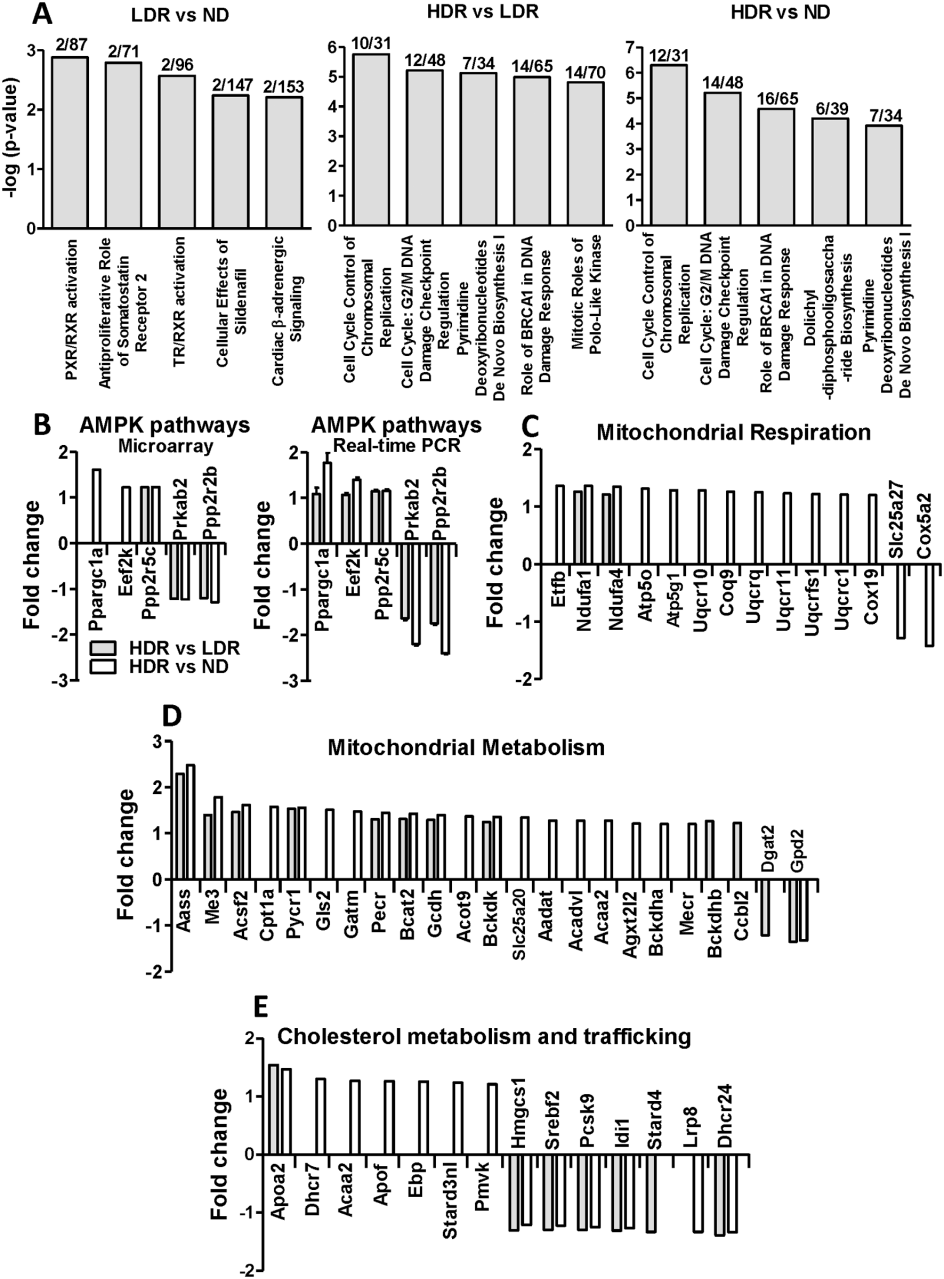
mRNA profiling analysis of control and DIO mice. (A) Top five most significant enriched canonical pathways identified by Ingenuity Pathway Analysis (grey bars) using all the differentially expressed genes in LDR vs ND, HDR vs LDR and HDR vs ND islets comparisons that are listed according to their p-values expressed in—log (Y axis). The ratio of the number of differentially expressed genes over the total number of genes involved in each canonical pathway is reported on top of the bars. (B) AMPK pathways, (C) mitochondrial respiration, (D) mitochondrial metabolism and (E) cholesterol metabolism and trafficking gene expression by microarray analysis (B, C, D and E; +/- 1.2 fold change) and quantitative real time PCR (B; +/- 1.15 fold change) of HDR vs LDR (gray bar) and HDR vs ND (white bar).

Among the 28853 genes detected on the gene-chip mouse gene 1.0 ST array, 1508 genes were differentially expressed (i.e. their expression changed by more than ±1.2-fold) between HDR and ND islets (S2 Table). Of these, 883 genes were upregulated and 625 were downregulated in HDR islets. LDR vs ND comparison showed the fewest expression differences (17 altogether) with only 14 genes upregulated and 3 genes downregulated in the LDR group (S3 Table). The surprising observation of extremely few expression changes despite the very different diet, is consistent with the ‘compensation’ phenotype of the LDR group and is reflected by the similar serum biochemistry results obtained from LDR and ND mice (Fig 5). In the HDR vs LDR comparison, we found 1041 genes to be differentially expressed in HDR islets, of which 523 were upregulated and 518 were downregulated (S4 Table). Pathway enrichment analysis identified the top five enriched canonical pathways for each comparison (Fig 6A). Major changes occurred in pathways related to cell-cycle and proliferation in the HDR vs ND comparison and in the HDR vs LDR comparison, which correlated with increased β-cell mass and proliferation index in HDR islets [22].

As islets from DIO mice showed alterations in AMPK activity and signalling, we also examined gene expression changes in this pathway and found decreased AMPK-β subunit (*Prkab2*). Other genes that were altered in this pathway included the downstream direct targets, *Eef2k* and *Ppargc1a* and the upstream genes *Ppp2r2b* and *Ppp2r5c*, particularly in the HDR vs ND comparison (Fig 6B). These expression changes in genes related to AMPK pathways were validated by real time PCR (Fig 6B). Several genes related to mitochondrial respiration and metabolism were differentially expressed in the HDR islets (Fig 6C and 6D), along with 17 genes coding for mitochondrial proteins (S2 and S4 Tables). Finally, we observed changes in the expression of various genes related to cholesterol metabolism and trafficking in HDR islets (Fig 6E), in keeping with the elevated cholesterol synthesis and content in HDR islets. LDR islets in comparison to ND showed almost no change in the expression levels of the same pathways except for *Cpt1* and *Ppargc1a* that reflect enhanced fat oxidation, as expected in animals fed a high fat diet.

## Discussion

The present study reveals some of the underlying causes for islet β-cell dysfunction and defective GSIS in HFD fed mice (both LDR and HDR). The results implicate altered mitochondrial function, decreased AMPK activity and elevated expression of PKCε as key factors. However, unlike most DIO mouse studies, the animals were stratified with the goal of identifying specific differences that characterize a largely ‘prediabetic’ compensatory state (LDR group) from an ‘early diabetic’ state (HDR mice). This comparison revealed the following key differences that will be discussed below: 1) a more pronounced mitochondrial dysfunction with marked mitochondrial membrane hyperpolarization; 2) a suppression of active AMPK relative to total AMPK; 3) reduced ACC activity, in association with enhanced fat oxidation [22]; 4) increased HMGCR activity in association with elevated islet cholesterol synthesis and plasma cholesterol; and finally 5) widespread changes in islet gene expression.

The Δψ_mito_ results indicating mitochondrial hyperpolarization at a high glucose concentration in HDR islets could be due to fewer protons reentering mitochondria via the ATP-synthase complex with a resultant decrease in ATP production [33]. Interestingly, Wikstrom *et al*. [34] observed an increased number of hyperpolarized mitochondria following a 24 h glucolipotoxic treatment of dispersed islet β-cells, suggesting that mitochondrial hyperpolarization can be associated with β-cell dysfunction under metabolic stress. Also, we previously observed that HDR islets show increased FFA oxidation compared to LDR and ND islets, without a change in glucose oxidation [22], which could lead to an increase in the shuttling of protons into the mitochondrial inter-membrane space. This could explain the increase in Δψ_mito_ observed in HDR islets. Altered mitochondrial reactive oxygen species (ROS) production could contribute to β-cells dysfunction in HDR islets since hyperpolarization of Δψ_mito_ is associated with ROS production [35], and β-cells exposed to a glucolipotoxic insult show ROS accumulation both *in vitro* and *in vivo* [36,37]. However, the current ROS measurement tools are not very reliable and additional work is needed to test this possibility. Uncoupling did not appear to be a contributor to the lowered ATP production in DIO islets, like uncoupled respiration was unchanged in LDR and HDR islets. A recent study identified the accumulation of carnitine esters of hydroxylated FFA, in particular 3-hydroxytetradecanoylcarnitine in isolated islets exposed to a glucolipotoxic insult [38]. Whether DIO islets also show this alteration that may cause dysfunction of mitochondrial energy metabolism [26] is not known.

We further examined the possibility that the decreased ATP turnover and content in DIO mouse islets affects the activity of the energy sensor AMPK [28] which has been implicated in the control of insulin secretion, β-cell growth and apoptosis [32,36,39]. AMPK phosphorylation of ACC and HMGCR inactivates these enzymes to respectively promote β-oxidation of FFA [40] and to reduce cholesterol biosynthesis [41]. AMPK activation also reduces GSIS [32,42] and β-cell proliferation [39,43]. We found that despite an increase in total AMPK protein content in both LDR and HDR islets, phosphorylation status of AMPK under basal conditions was greatly decreased. Also net glucose-responsive AMPK activity in LDR and HDR islets was reduced, with changes in the HDR islets being more pronounced. Thus, the gradual decline in β-cell function from LDR to HDR islets correlates with corresponding decreases in AMPK activation.

Interestingly, despite the decreased AMPK activation in DIO islets, there was no corresponding effect on ACC phosphorylation: in fact, ACC phosphorylation was slightly elevated in HDR islets. The disassociation between AMPK phosphorylation and ACC phosphorylation appears to be unique to the HDR islets and resembles the dissociation observed in normal muscle during prolonged exercise, where is correlated with a shift in fuel utilization from glucose to FFA [44,45]. The reason for this dissociation in islets is unclear. Inasmuch as total AMPK levels increase in HDR islets, the possibility that the dephosphorylated AMPK shows some activity towards ACC and thus causes elevated phospho-ACC needs to be considered. Nevertheless, the reduced activity of ACC due to its increased phosphorylation is expected to lower malonyl-CoA production in HDR islets so that mitochondrial β-oxidation is elevated, as observed previously [22]. This could be part of an adaptive protective mechanism important for nutrient detoxification at the expense of normal insulin secretion in HDR islets.

AMPK signaling is also regulated by protein phosphatase PP2a and a recent study showed that the regulatory subunit of protein phosphatase PP2a (PPP2R5C) plays an important role in the regulation of glucose and lipid metabolic homeostasis in hepatocytes, at least in part by regulating AMPK activation. In keeping with this possibility, PPP2R5C knockdown in rat hepatocytes has been shown to elevate AMPK phosphorylation [46]. In addition, we noticed that the *Ppp2r5c* gene was up-regulated in HDR islets and this might have diminished AMPK activation under the DIO conditions. Besides, it has been shown that glucose enhances PP2a activity, which activates ACC, probably by its dephosphorylation [47,48]. In fact a dissociation between phospho-AMPK and phospho-ACC levels has been noticed in renal tissue, in a recent study in high fat diet fed mice [49]. Thus an altered PP2a / PPP2R5C balance in DIO islets may be responsible for the dissociation between AMPK and ACC activity.

Even though glucose regulates AMPK activity, we did not notice a direct regulation of HMGCR phosphorylation / activity and cholesterol content by glucose in ND or DIO islets. However, the enhanced activity of HMGCR in HDR islets is possibly due to the inhibition of the AMPK pathway, contributing to the elevated cholesterol levels in these islets. Various studies have provided support for the view that cholesterol accumulation alters mitochondrial function and membrane fusion processes in various cell types [50]. In the β-cell, cholesterol accumulation has been implicated in the reduced exocytosis that occurs in T2D [51,52]. Also, a recent study showed that isoprenoid intermediates of cholesterol biosynthesis are important for GSIS [53]. Thus, increased cholesterol levels, such as are observed in HDR but not LDR islets, could contribute to the markedly defective GSIS and β-cell failure in these mice.

The marked reduction in AMPK activity in HDR islets could contribute to the β-cell dysfunction of DIO islets by altering its cholesterol metabolism as well as the enhanced β-cell mass seen in our earlier study [22]. Thus, AMPK via its interaction with mTOR and other growth control pathways [28] is a major player in cell proliferation, and its activity is inversely correlated with β-cell growth [39]. Interestingly, the major gene expression changes seen in HDR vs ND and in HDR vs LDR comparisons in the present study were related to cell cycle, cell proliferation and DNA replication and repair, all of which could relate to the much increased β-cell mass and proliferation seen in HDR mice [22].

Lipid metabolism in the pancreatic β-cell, in particular the GL/FFA cycle, potentiates GSIS through the production of lipid-signaling molecules (e.g., diacylglycerol, 1-monoacylglycerol) [7,14,54]. Sn1,2-diacylglycerol, generated either by phospholipid hydrolysis or via the lipogenic arm of the GL/FFA cycle can activate certain members of protein kinase C family, including PKCε, which has been identified as a negative regulator of lipolysis and GSIS in pancreatic β-cells [11]. We noticed a two-fold increase in PKCε total protein levels in LDR and HDR islets, and observed an increase of PKCε phosphorylation, a potential indicator of PKCε activation [55] only in HDR islets. These changes may contribute to the secretory dysfunction and altered lipid partitioning observed in both LDR and HDR islets [22].

A most surprising observation of this study was the similarity in gene expression profiles between LDR and ND islets (with only 17 genes being differentially expressed by more than ±1.2 fold) compared to the differences seen between HDR and ND islets (1508 differentially regulated genes) or HDR and LDR islets (1041 differentially regulated genes). Since HDR and LDR mice were fed the same high fat diet, it would be expected that a high fat diet shift would cause major changes in gene expression in all DIO mice, which in fact was not the case. The possibility should be considered that β-cell compensation in the prediabetic state (corresponding to the LDR group) is largely independent from transcriptional adaptive changes whereas the transition to early diabetes (HDR mice) results in part from major alterations in gene expression. The reason why some animals respond better to the obesogenic diet (HDR) than others (LDR) is not known. It could be related to subtle epigenetic differences that could impact in particular on appetite since HDR animals eat slightly more than LDR [22]. The compensation in the LDR group could be achieved via mechanisms independent of modifications in mRNA expression such as translational and posttranslational changes. For example the mRNA expression of AMPK subunits is unchanged in LDR islets, whereas the markedly reduced AMPK activity could drive compensatory effects such as β-cell growth.

## Conclusion

**Fig 7:**
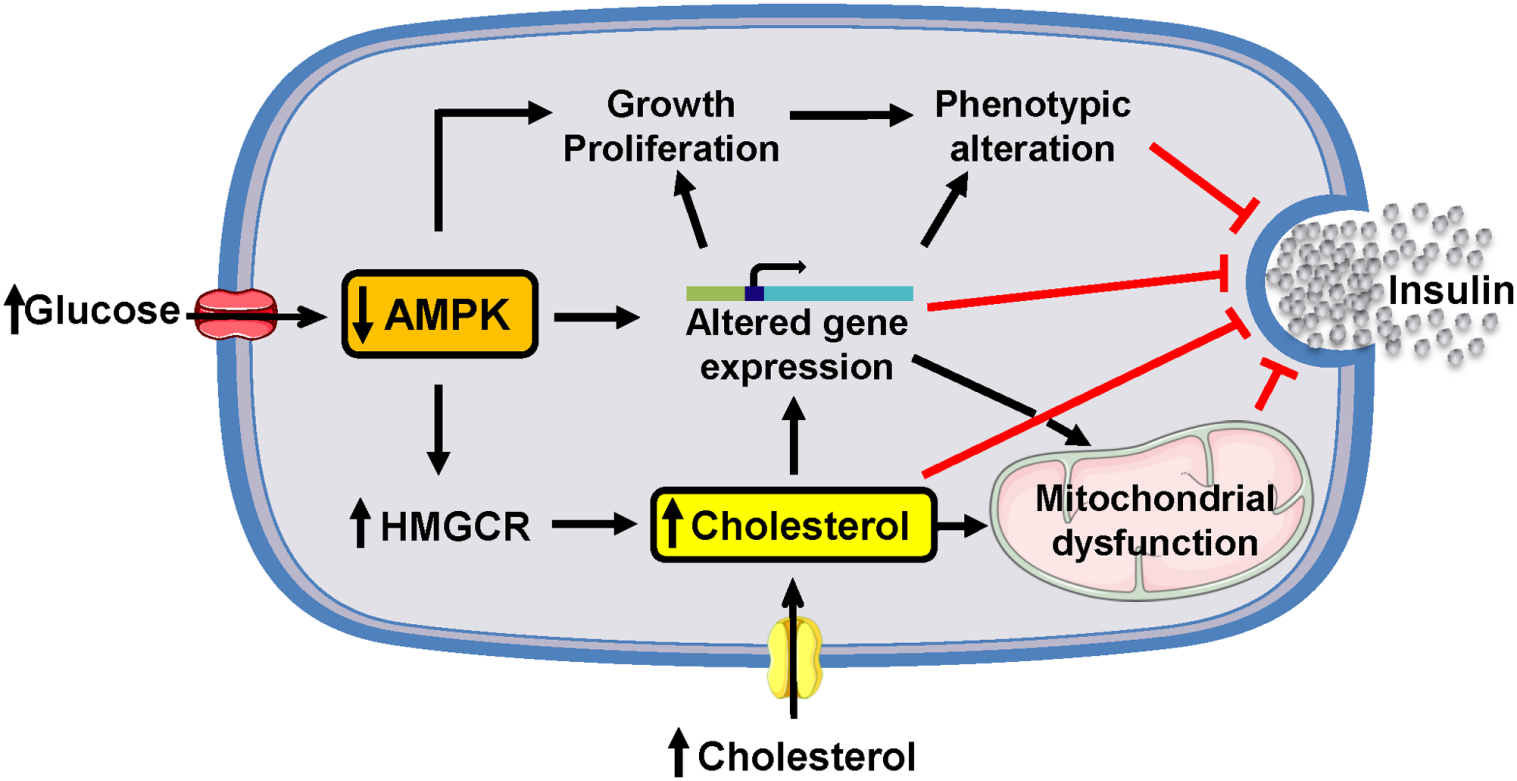
Model depicting the mechanisms contributing to β-cell dysfunction and failure in obese HDR mice. Hyperglycemic and hypercholesterolemic HDR mice are characterized by major changes in islet mRNA species expression, and suppression of AMPK activity that causes HMG-CoA reductase activation and increased cholesterol synthesis and deposition in islet β-cells. Cholesterol accumulation leads to mitochondrial dysfunction and reduced exocytotic release of insulin. Reduced AMPK activity may promote β-cell growth and proliferation as a compensatory mechanism, but can also lead to altered gene expression and modify various signaling pathways for insulin secretion, causing β-cell phenotypic alterations and defective insulin secretion.

This study provides insights into the biochemical basis of islet β-cell failure in HDR DIO mice (Fig 7). The results favor the view that the pre-eminent β-cell dysfunction of HDR mice is due at least in part to chronically reduced AMPK activity, mitochondrial dysfunction with decreased ATP production, elevated cholesterol uptake, biosynthesis and deposition, and vast alterations in gene expression. The latter could be related to compensatory changes in β-cell growth that modify its phenotype as well as GSIS.

## Supporting Information

S1 Table PCR primer sequences used for Quantitative Real Time PCR. (DOCX)

S2 Table Functional classification of differentially expressed genes in HDR *vs* ND islets. (DOCX)

S3 Table Functional classification of differentially expressed genes in LDR *vs* ND islets. (DOCX)

S4 Table Functional classification of differentially expressed genes in HDR *vs* LDR islets. (DOCX)
