# Supplementary material for: Pancreatic β-Cell Dysfunction in Diet-Induced Obese Mice: Roles of AMP-Kinase, Protein Kinase Cε, Mitochondrial and Cholesterol Metabolism, and Alterations in Gene Expression
Source: PLoS One. 2016 Apr 4;11(4):e0153017. doi: 10.1371/journal.pone.0153017 (PMC4820227; doi:10.1371/journal.pone.0153017)
Supplement: S1 Table — (DOCX) [file pone.0153017.s001.docx]

**S1 Table. PCR primer sequences used for Quantitative Real Time PCR.**

Primers: S, sense; AS, antisense.

| GENE | GenBank accession no | Primer sequences (5’-3’) |
| --- | --- | --- |
| Eef2k | NM_007908.4 | S: GTGAATCAGAGCACCAGGCT |
|  |  | AS: ATCCCCGGAGTTCTCTGACA |
| Ppargc1a | NM_008904.2 | S: TAGAGTGTGCTGCTCTGGTTG |
|  |  | AS: GATTGGTCGCTACACCACTTC |
| *Ppp2r2b* | NM_028392.3 | S: AATTCAACCACACGGGAGAG |
|  |  | AS: GGTTCATGGCTCTGGAATGT |
| Ppp2r5c | NM_012023.3 | S: TATATCACCCACAACCGGAAC |
|  |  | AS: ACGTTGGTTCATCCTCTTCTG |
| Prkab2 | NM_182997.2 | S: CATCTCTGGGTCCTTCAACAA |
|  |  | AS: TACTGATGCTCTCCCTCTGGA |
| Beta-Actin | NM_007393.5 | S: CATGGATGACGATATCGCTGC |
|  |  | AS:GTACGACCAGAGGCATACAGG |
