# Supplementary material for: Pancreatic β-Cell Dysfunction in Diet-Induced Obese Mice: Roles of AMP-Kinase, Protein Kinase Cε, Mitochondrial and Cholesterol Metabolism, and Alterations in Gene Expression
Source: PLoS One. 2016 Apr 4;11(4):e0153017. doi: 10.1371/journal.pone.0153017 (PMC4820227; doi:10.1371/journal.pone.0153017)
Supplement: S2 Table — (DOCX) [file pone.0153017.s002.docx]

S2 table. Functional classification of differentially expressed genes in HDR vs ND islets.

| **Gene Symbol** | **Gene description** | **FDR step up (p < 0.05 = 1508)** | **Fold-Change Increase: 883 Decrease: 625** |
| --- | --- | --- | --- |
| **Carbohydrate metabolism** | | | |
| Me3 | malic enzyme 3, NADP(+)-dependent, mitochondrial | 2,68E-06 | 1,786 |
| Gmds | GDP-mannose 4, 6-dehydratase | 8,13E-05 | 1,529 |
| Bpgm | 2,3-bisphosphoglycerate mutase | 2,25E-04 | 1,349 |
| Gla | galactosidase, alpha | 2,45E-04 | 1,316 |
| Mpdu1 | mannose-P-dolichol utilization defect 1 | 3,58E-04 | 1,307 |
| Pgk1 | phosphoglycerate kinase 1 | 3,29E-04 | 1,272 |
| Pgls | 6-phosphogluconolactonase | 4,94E-04 | 1,245 |
| Galns | galactosamine (N-acetyl)-6-sulfate sulfatase | 4,04E-03 | 1,217 |
| Mdh1 | malate dehydrogenase 1, NAD (soluble) | 3,38E-04 | 1,212 |
| Gale | galactose-4-epimerase, UDP | 3,10E-02 | 1,209 |
| Tsta3 | tissue specific transplantation antigen P35B | 1,32E-03 | 1,209 |
| Pgm5 | phosphoglucomutase 5 | 2,38E-02 | -1,215 |
| Ppp1r1a | protein phosphatase 1, regulatory (inhibitor) subunit 1A | 5,67E-04 | -1,227 |
| Rbp4 | retinol binding protein 4, plasma | 1,16E-02 | -1,235 |
| Slc2a2 | solute carrier family 2 (facilitated glucose transporter), member 2 | 1,20E-02 | -1,241 |
| Rpia | ribose 5-phosphate isomerase A | 4,31E-03 | -1,390 |
| Cryl1 | crystallin, lambda 1 | 6,60E-04 | -1,411 |
| Khk | ketohexokinase | 1,97E-03 | -1,461 |
| Aldoc | aldolase C, fructose-bisphosphate | 7,33E-04 | -1,511 |
| Hpse | heparanase | 1,06E-03 | -1,779 |
| **Glycan metabolism** | | | |
| Alg8 | asparagine-linked glycosylation 8 homolog (yeast, Alpha-1,3-Glucosyltransferase) | 1,92E-03 | 1,417 |
| Dpm3 | dolichyl-phosphate mannosyltransferase polypeptide 3 | 7,03E-04 | 1,381 |
| Alg3 | asparagine-linked glycosylation 3 homolog (yeast, Alpha-1,3-Mannosyltransferase) | 3,07E-03 | 1,357 |
| Pomgnt1 | protein O-linked mannose beta1,2-N-acetylglucosaminyltransferase | 9,03E-06 | 1,354 |
| Alg12 | asparagine-linked glycosylation 12 homolog (yeast, Alpha-1,6-Mannosyltransferase) | 1,18E-04 | 1,344 |
| Alg14 | asparagine-linked glycosylation 14 homolog (yeast) | 7,77E-03 | 1,342 |
| Glb1 | galactosidase, beta 1 | 9,30E-06 | 1,340 |
| Pomt1 | protein-O-mannosyltransferase 1 | 2,65E-04 | 1,316 |
| Alg5 | asparagine-linked glycosylation 5 homolog (yeast, Dolichyl-Phosphate Beta-Glucosyltransferase) | 4,23E-04 | 1,299 |
| Alg6 | asparagine-linked glycosylation 6 homolog (yeast, Alpha-1,3-Glucosyltransferase) | 3,06E-03 | 1,263 |
| Man2b1 | mannosidase 2, alpha B1 | 6,56E-06 | 1,247 |
| St3gal1 | ST3 beta-galactoside alpha-2,3-sialyltransferase | 1,39E-03 | 1,239 |
| Hs3st1 | heparan sulfate (glucosamine) 3-O-sulfotransferase 1 | 6,39E-05 | -1,250 |
| **Amino acid metabolism** | | | |
| Aass | aminoadipate-semialdehyde synthase | 2,35E-04 | 2,476 |
| Pycr1 | pyrroline-5-carboxylate reductase 1 | 1,37E-05 | 1,556 |
| Gls2 | glutaminase 2 (liver, mitochondrial) | 3,99E-03 | 1,511 |
| Psat1 | phosphoserine aminotransferase 1 | 7,99E-04 | 1,503 |
| Gatm | glycine amidinotransferase (L-arginine:glycine amidinotransferase) | 8,03E-04 | 1,472 |
| Bcat2 | branched chain aminotransferase 2, mitochondrial | 4,84E-04 | 1,429 |
| Gcdh | glutaryl-Coenzyme A dehydrogenase | 9,39E-05 | 1,398 |
| Odc1 | ornithine decarboxylase, structural 1 | 4,08E-03 | 1,358 |
| Bckdk | branched chain ketoacid dehydrogenase kinase | 6,08E-05 | 1,355 |
| Grhpr | glyoxylate reductase/hydroxypyruvate reductase | 2,92E-04 | 1,325 |
| Phgdh | 3-phosphoglycerate dehydrogenase | 1,69E-02 | 1,305 |
| Gamt | guanidinoacetate methyltransferase | 3,93E-02 | 1,303 |
| Pycr2 | pyrroline-5-carboxylate reductase family, member 2 | 6,27E-05 | 1,300 |
| Aadat | aminoadipate aminotransferase | 5,33E-03 | 1,277 |
| Got1 | glutamate oxaloacetate transaminase 1, soluble | 2,94E-04 | 1,267 |
| Ckb | creatine kinase, brain | 4,39E-03 | 1,266 |
| Cad | carbamoyl-phosphate synthetase 2, aspartate transcarbamylase | 1,26E-02 | 1,251 |
| Vars | valyl-tRNA synthetase | 7,44E-04 | 1,230 |
| Aoc3 | amine oxidase, copper containing 3 | 5,58E-03 | 1,224 |
| Aldh9a1 | aldehyde dehydrogenase 9, subfamily A1 | 2,34E-05 | 1,219 |
| Srm | spermidine synthase | 3,85E-02 | 1,213 |
| Agxt2l2 | alanine-glyoxylate aminotransferase 2-like 2 | 8,76E-03 | 1,210 |
| Bckdha | branched chain ketoacid dehydrogenase E1, alpha polypeptide | 1,24E-02 | 1,204 |
| Pycrl | pyrroline-5-carboxylate reductase-like | 1,56E-03 | 1,201 |
| Kynu | kynureninase (L-kynurenine hydrolase) | 4,99E-02 | -1,226 |
| Mat2a | methionine adenosyltransferase II, alpha | 1,46E-03 | -1,371 |
| Gad1 | glutamic acid decarboxylase 1 | 1,44E-02 | -1,487 |
| Ass1 | argininosuccinate synthetase 1 | 1,39E-03 | -1,530 |
| **Nucleotide/pyrophosphate metabolism** | | | |
| Gucy2c | guanylate cyclase 2c | 8,62E-07 | 3,598 |
| Rrm2 | ribonucleotide reductase M2 | 9,34E-05 | 2,071 |
| Tyms | thymidylate synthase | 3,32E-03 | 1,437 |
| Rasl10b | RAS-like, family 10, member B | 6,67E-04 | 1,415 |
| Ppa1 | pyrophosphatase (inorganic) 1 | 5,99E-04 | 1,387 |
| Dhfr | dihydrofolate reductase | 3,19E-03 | 1,354 |
| Impdh2 | inosine 5'-phosphate dehydrogenase 2 | 2,53E-03 | 1,302 |
| Adcy4 | adenylate cyclase 4 | 3,68E-03 | 1,291 |
| Nme2 | Nucleoside Diphosphate Kinase 2 | 1,38E-04 | 1,284 |
| Ctps | cytidine 5'-triphosphate synthase | 1,52E-02 | 1,274 |
| Guk1 | guanylate kinase 1 | 1,22E-04 | 1,230 |
| Gda | guanine deaminase | 2,75E-02 | 1,212 |
| Sult3a1 | sulfotransferase family 3A, member 1 | 3,74E-03 | 1,209 |
| Impdh1 | inosine 5'-phosphate dehydrogenase 1 | 1,66E-03 | 1,202 |
| Nme1 | Nucleoside Diphosphate Kinase 1 | 3,50E-03 | 1,202 |
| Nme5 | Nucleoside Diphosphate Kinase 5 | 4,50E-03 | -1,260 |
| Nme4 | Nucleoside Diphosphate Kinase 4 | 6,39E-05 | -1,278 |
| **Lipid metabolism** | | | |
| Pnliprp2 | pancreatic lipase-related protein 2 | 3,25E-02 | 2,417 |
| Acsf2 | acyl-CoA synthetase family member 2 | 2,52E-04 | 1,614 |
| Cel | carboxyl ester lipase | 4,56E-02 | 1,613 |
| Cpt1a | carnitine palmitoyltransferase 1a, liver | 5,29E-06 | 1,576 |
| Lmf1 | lipase maturation factor 1 | 7,13E-06 | 1,552 |
| Cd36 | CD36 antigen | 2,90E-02 | 1,505 |
| Pecr | peroxisomal trans-2-enoyl-CoA reductase | 2,43E-05 | 1,448 |
| Fabp4 | fatty acid binding protein 4, adipocyte | 4,16E-04 | 1,436 |
| Acer2 | alkaline ceramidase 2 | 1,28E-02 | 1,423 |
| Ces2g | carboxylesterase 2G | 9,26E-04 | 1,417 |
| Acot9 | acyl-CoA thioesterase 9 | 2,81E-03 | 1,371 |
| Slc25a20 | solute carrier family 25 (mitochondrial carnitine/acylcarnitine translocase) member 20 | 3,05E-04 | 1,345 |
| Ppapdc1b | phosphatidic acid phosphatase type 2 domain containing 1B | 2,89E-05 | 1,344 |
| Mgll | monoglyceride lipase | 5,52E-03 | 1,287 |
| Hsd3b7 | hydroxy-delta-5-steroid dehydrogenase, 3 beta- and steroid Delta isomerase-7 | 1,37E-03 | 1,284 |
| Acadvl | acyl-Coenzyme A dehydrogenase, very long chain | 7,35E-05 | 1,276 |
| Elovl7 | ELOVL family member 7, elongation of long chain fatty acid | 4,32E-04 | 1,270 |
| Cds1 | CDP-diacylglycerol synthase 1 | 7,17E-03 | 1,266 |
| Pemt | phosphatidylethanolamine N-methyltransferase | 6,01E-03 | 1,249 |
| Cyp2j6 | cytochrome P450, family 2, subfamily j, polypeptide 6 | 6,90E-04 | 1,248 |
| Pla2g12a | phospholipase A2, group XIIA | 8,63E-05 | 1,234 |
| Mboat1 | membrane bound O-acyltransferase domain containing 1 | 2,29E-02 | 1,232 |
| Hsd17b12 | hydroxysteroid (17-beta) dehydrogenase 12 | 2,36E-03 | 1,221 |
| Anxa3 | annexin A3 | 3,28E-03 | 1,221 |
| Gde1 | glycerophosphodiester phosphodiesterase 1 | 8,15E-05 | 1,216 |
| Lipa | lysosomal acid lipase A | 6,97E-03 | 1,210 |
| Acot11 | acyl-CoA thioesterase 11 | 3,88E-03 | 1,206 |
| Mecr | mitochondrial trans-2-enoyl-CoA reductase | 2,93E-03 | 1,200 |
| Scd3 | stearoyl-coenzyme A desaturase 3 | 1,77E-03 | -1,207 |
| Arv1 | ARV1 homolog (yeast) | 1,20E-03 | -1,207 |
| Asah1 | N-acylsphingosine amidohydrolase 1 | 1,14E-04 | -1,223 |
| Osbpl6 | oxysterol binding protein-like 6 | 5,12E-03 | -1,226 |
| Cyp2s1 | cytochrome P450, family 2, subfamily s, polypeptide 1 | 3,35E-03 | -1,270 |
| Apod | apolipoprotein D | 3,14E-02 | -1,272 |
| Ugt8a | UDP galactosyltransferase 8A | 1,96E-02 | -1,276 |
| Gpd2 | glycerol phosphate dehydrogenase 2, mitochondrial | 1,49E-02 | -1,326 |
| Scd2 | stearoyl-Coenzyme A desaturase 2 | 3,73E-06 | -1,390 |
| Gpr120 | G protein-coupled receptor 120 | 6,67E-04 | -1,408 |
| Lpl | lipoprotein lipase | 1,02E-04 | -1,675 |
| Scd1 | stearoyl-Coenzyme A desaturase 1 | 2,05E-04 | -2,115 |
| **Cholesterol metabolism and transport** | | | |
| Apoa2 | apolipoprotein A-II | 1,49E-03 | 1,469 |
| Dhcr7 | 7-dehydrocholesterol reductase | 7,44E-04 | 1,302 |
| Acaa2 | acetyl-Coenzyme A acyltransferase 2 | 8,93E-04 | 1,271 |
| Apof | apolipoprotein F | 9,32E-03 | 1,265 |
| Ebp | phenylalkylamine Ca2+ antagonist (emopamil) binding protein | 6,25E-04 | 1,260 |
| Stard3nl | STARD3 N-terminal like | 3,66E-05 | 1,239 |
| Pmvk | phosphomevalonate kinase | 7,22E-04 | 1,210 |
| Hmgcs1 | 3-hydroxy-3-methylglutaryl-Coenzyme A synthase 1 | 4,55E-02 | -1,211 |
| Klb | klotho beta | 2,21E-02 | -1,218 |
| Srebf2 | sterol regulatory element binding factor 2 | 2,51E-03 | -1,223 |
| Pcsk9 | proprotein convertase subtilisin/kexin type 9 | 5,32E-03 | -1,247 |
| Idi1 | isopentenyl-diphosphate delta isomerase | 2,82E-02 | -1,264 |
| Osbp2 | oxysterol binding protein 2 | 2,59E-04 | -1,297 |
| Lrp8 | low density lipoprotein receptor-related protein 8, apolipoprotein | 1,67E-04 | -1,332 |
| Dhcr24 | 24-dehydrocholesterol reductase | 1,20E-03 | -1,336 |
| **Metabolism-miscellaneous** | | | |
| Aldh1a3 | aldehyde dehydrogenase family 1, subfamily A3 | 2,25E-04 | 3,241 |
| Gc | group specific component | 8,62E-07 | 1,893 |
| Hsd17b13 | hydroxysteroid (17-beta) dehydrogenase 13 | 4,09E-02 | 1,711 |
| Gsto2 | glutathione S-transferase omega 2 | 1,83E-03 | 1,605 |
| Mosc2 | MOCO sulphurase C-terminal domain containing 2 | 2,68E-05 | 1,493 |
| Bcmo1 | beta-carotene 15,15'-monooxygenase | 9,04E-05 | 1,480 |
| Nans | N-acetylneuraminic acid synthase (sialic acid synthase) | 8,52E-05 | 1,471 |
| Gsto1 | glutathione S-transferase omega 1 | 2,08E-04 | 1,418 |
| Iyd | iodotyrosine deiodinase | 8,51E-04 | 1,414 |
| Cmas | cytidine monophospho-N-acetylneuraminic acid synthetase | 2,89E-05 | 1,411 |
| Dio1 | deiodinase, iodothyronine, type I | 8,28E-06 | 1,400 |
| Dhrs3 | dehydrogenase/reductase (SDR family) member 3 | 4,80E-03 | 1,345 |
| Fpgs | folylpolyglutamyl synthetase | 7,53E-03 | 1,310 |
| Ephx3 | epoxide hydrolase 3 | 1,77E-02 | 1,272 |
| Tmem14c | transmembrane protein 14C | 2,56E-03 | 1,258 |
| Art3 | ADP-ribosyltransferase 3 | 2,09E-03 | 1,257 |
| Retsat | retinol saturase (all trans retinol 13,14 reductase) | 1,94E-02 | 1,253 |
| Ggh | gamma-glutamyl hydrolase | 1,98E-02 | 1,228 |
| Coasy | Coenzyme A synthase | 8,93E-04 | 1,216 |
| Chpf2 | chondroitin polymerizing factor 2 | 3,41E-02 | 1,214 |
| Vkorc1 | vitamin K epoxide reductase complex, subunit 1 | 6,25E-03 | 1,211 |
| Pnpo | pyridoxine 5'-phosphate oxidase | 8,37E-04 | 1,206 |
| Gstm7 | glutathione S-transferase, mu 7 | 9,37E-03 | -1,225 |
| Gstt1 | glutathione S-transferase, theta 1 | 3,30E-03 | -1,226 |
| Nos1 | nitric oxide synthase 1, neuronal | 4,42E-02 | -1,238 |
| Fmo5 | flavin containing monooxygenase 5 | 1,79E-03 | -1,414 |
| Gstm3 | glutathione S-transferase, mu 3 | 3,91E-05 | -1,453 |
| Inmt | indolethylamine N-methyltransferase | 1,70E-02 | -1,459 |
| Gstm1 | glutathione S-transferase, mu 1 | 1,32E-04 | -1,615 |
| Ndst4 | N-deacetylase/N-sulfotransferase (heparin glucosaminyl) 4 | 8,71E-04 | -1,832 |
| **Mitochondrial respiration** | | | |
| Etfb | electron transferring flavoprotein, beta polypeptide | 3,34E-05 | 1,360 |
| Ndufa1 | NADH dehydrogenase (ubiquinone) 1 alpha subcomplex, 1 | 3,81E-04 | 1,360 |
| Ndufa4 | NADH dehydrogenase (ubiquinone) 1 alpha subcomplex, 4 | 1,19E-03 | 1,350 |
| Atp5o | ATP synthase, H+ transporting, mitochondrial F1 complex, O subunit | 8,13E-05 | 1,312 |
| Atp5g1 | ATP synthase, H+ transporting, mitochondrial F0 complex, subunitC1 (subunit 9) | 3,35E-03 | 1,286 |
| Uqcr10 | ubiquinol-cytochrome c reductase, complex III subunit X | 1,60E-03 | 1,283 |
| Coq9 | coenzyme Q9 homolog (yeast) | 1,54E-06 | 1,260 |
| Uqcrq | ubiquinol-cytochrome c reductase, complex III subunit VII | 5,73E-05 | 1,251 |
| Uqcr11 | ubiquinol-cytochrome c reductase, complex III subunit XI | 1,74E-03 | 1,231 |
| Uqcrfs1 | ubiquinol-cytochrome c reductase, Rieske iron-sulfur polypeptide 1 | 5,75E-04 | 1,217 |
| Uqcrc1 | ubiquinol-cytochrome c reductase core protein 1 | 4,23E-03 | 1,208 |
| Cox19 | COX19 cytochrome c oxidase assembly homolog (S. cerevisiae) | 1,55E-03 | 1,201 |
| Slc25a27 | solute carrier family 25, member 27 (UCP4) | 5,46E-03 | -1,286 |
| Cox6a2 | cytochrome c oxidase, subunit VI a, polypeptide 2 | 6,59E-03 | -1,422 |
| **Oxidation-reduction process** | | | |
| Dhrs7 | dehydrogenase/reductase (SDR family) member 7 | 1,02E-04 | 1,360 |
| Dhrs7b | dehydrogenase/reductase (SDR family) member 7B | 9,04E-05 | 1,287 |
| BC003331 | cDNA sequence BC003331 | 2,82E-04 | 1,250 |
| Dhrs1 | dehydrogenase/reductase (SDR family) member 1 | 9,80E-05 | 1,213 |
| Tmx3 | thioredoxin-related transmembrane protein 3 | 5,64E-04 | -1,231 |
| Akr1c12 | aldo-keto reductase family 1, member C12 | 8,53E-04 | -1,279 |
| Akr1c19 | aldo-keto reductase family 1, member C19 | 1,26E-04 | -1,503 |
| **Cell cycle** | | | |
| Ccnb1 | cyclin B1 | 3,73E-04 | 3,189 |
| Top2a | topoisomerase (DNA) II alpha | 4,65E-04 | 2,927 |
| Mki67 | antigen identified by monoclonal antibody Ki 67 | 1,90E-04 | 2,871 |
| Reg2 | regenerating islet-derived 2 | 1,10E-02 | 2,767 |
| Ccnb2 | cyclin B2 | 2,04E-04 | 2,736 |
| Anln | anillin, actin binding protein | 3,95E-04 | 2,683 |
| Kif11 | kinesin family member 11 | 2,08E-04 | 2,595 |
| Plk1 | polo-like kinase 1 (Drosophila) | 8,51E-04 | 2,585 |
| Bub1 | budding uninhibited by benzimidazoles 1 homolog (S. cerevisiae) | 9,80E-04 | 2,568 |
| Ect2 | ect2 oncogene | 2,95E-04 | 2,511 |
| Nek2 | NIMA (never in mitosis gene a)-related expressed kinase 2 | 4,09E-04 | 2,377 |
| Tpx2 | TPX2, microtubule-associated protein homolog (Xenopus laevis) | 5,81E-04 | 2,375 |
| Prc1 | protein regulator of cytokinesis 1 | 2,68E-04 | 2,372 |
| Ccna2 | cyclin A2 | 6,91E-04 | 2,343 |
| D2Ertd750e | DNA segment, Chr 2, ERATO Doi 750, expressed | 2,27E-04 | 2,331 |
| Casc5 | cancer susceptibility candidate 5 | 6,48E-04 | 2,272 |
| Stmn1 | stathmin 1 | 8,78E-04 | 2,228 |
| Cdk1 | cyclin-dependent kinase 1 | 2,66E-04 | 2,220 |
| Cdc20 | cell division cycle 20 homolog (S. cerevisiae) | 1,74E-04 | 2,207 |
| Dtl | denticleless homolog (Drosophila) | 2,48E-04 | 2,169 |
| Cks2 | CDC28 protein kinase regulatory subunit 2 | 6,82E-04 | 2,147 |
| Nusap1 | nucleolar and spindle associated protein 1 | 9,07E-04 | 2,086 |
| Dlgap5 | discs, large (Drosophila) homolog-associated protein 5 | 6,03E-04 | 2,034 |
| Sgol2 | shugoshin-like 2 (S. pombe) | 1,18E-04 | 2,011 |
| Ncaph | non-SMC condensin I complex, subunit H | 7,53E-04 | 2,000 |
| Cenpe | centromere protein E | 1,21E-03 | 1,981 |
| Cenpf | centromere protein F | 1,08E-03 | 1,949 |
| C79407 | expressed sequence C79407 | 7,80E-04 | 1,945 |
| Ckap2l | cytoskeleton associated protein 2-like | 8,31E-04 | 1,927 |
| Ckap2 | cytoskeleton associated protein 2 | 2,92E-04 | 1,915 |
| Nuf2 | NUF2, NDC80 kinetochore complex component, homolog (S. cerevisiae) | 2,96E-04 | 1,903 |
| Aurkb | aurora kinase B | 2,56E-03 | 1,900 |
| Kif23 | kinesin family member 23 | 7,16E-04 | 1,867 |
| Kntc1 | kinetochore associated 1 | 3,86E-04 | 1,855 |
| Rad51 | RAD51 homolog (S. cerevisiae) | 2,72E-03 | 1,846 |
| Kif20a | kinesin family member 20A | 1,62E-03 | 1,837 |
| Cenpm | centromere protein M | 1,35E-04 | 1,827 |
| Aspm | asp (abnormal spindle)-like, microcephaly associated (Drosophila) | 1,55E-03 | 1,823 |
| Mastl | microtubule associated serine/threonine kinase-like | 8,70E-04 | 1,815 |
| Reg3b | regenerating islet-derived 3 beta | 1,38E-02 | 1,811 |
| Cdkn3 | cyclin-dependent kinase inhibitor 3 | 4,37E-04 | 1,810 |
| Mcm5 | minichromosome maintenance deficient 5, cell division cycle | 7,22E-04 | 1,801 |
| Aurka | aurora kinase A | 1,17E-03 | 1,791 |
| Ncapg | non-SMC condensin I complex, subunit G | 7,13E-04 | 1,761 |
| Mcm2 | minichromosome maintenance deficient 2 mitotin (S. cerevisiae) | 4,52E-04 | 1,748 |
| Cenpn | centromere protein N | 3,38E-04 | 1,740 |
| Bub1b | BUB1 mitotic checkpoint serine/threonine kinase B | 5,69E-04 | 1,739 |
| Ncapg2 | non-SMC condensin II complex, subunit G2 | 1,12E-03 | 1,731 |
| Foxm1 | forkhead box M1 | 1,26E-03 | 1,708 |
| Zwilch | Zwilch, kinetochore associated, homolog (Drosophila) | 6,90E-04 | 1,694 |
| Cgref1 | cell growth regulator with EF hand domain 1 | 2,07E-04 | 1,693 |
| Cenpa | centromere protein A | 2,71E-03 | 1,693 |
| Cdca3 | cell division cycle associated 3 | 2,09E-03 | 1,682 |
| Cenpk | centromere protein K | 2,08E-04 | 1,677 |
| Spag5 | sperm associated antigen 5 | 3,03E-03 | 1,648 |
| E2f1 | E2F transcription factor 1 | 1,06E-05 | 1,637 |
| Smc2 | structural maintenance of chromosomes 2 | 1,07E-03 | 1,627 |
| Fam111a | family with sequence similarity 111, member A | 3,90E-03 | 1,622 |
| Cdca8 | cell division cycle associated 8 | 5,09E-03 | 1,616 |
| E2f8 | E2F transcription factor 8 | 2,40E-03 | 1,604 |
| Kif20b | kinesin family member 20B | 7,72E-04 | 1,604 |
| Spc24 | SPC24, NDC80 kinetochore complex component, homolog (S. cerevisiae) | 2,10E-04 | 1,598 |
| Mad2l1 | MAD2 mitotic arrest deficient-like 1 (yeast) | 3,03E-03 | 1,582 |
| Kif4 | kinesin family member 4 | 6,56E-03 | 1,578 |
| Oip5 | Opa interacting protein 5 | 3,01E-03 | 1,578 |
| Cenpi | centromere protein I | 1,92E-03 | 1,562 |
| Racgap1 | Rac GTPase-activating protein 1 | 6,67E-04 | 1,559 |
| Mcm6 | minichromosome maintenance deficient 6 (MIS5 homolog, S. pombe) | 3,93E-03 | 1,557 |
| Cdca2 | cell division cycle associated 2 | 1,52E-03 | 1,553 |
| Hells | helicase, lymphoid specific | 2,46E-03 | 1,549 |
| Cks1b | CDC28 protein kinase 1b | 7,85E-04 | 1,545 |
| Ccnf | cyclin F | 4,36E-03 | 1,533 |
| Kif2c | kinesin family member 2C | 3,82E-03 | 1,519 |
| Ttk | Ttk protein kinase | 1,38E-03 | 1,517 |
| Cdc25c | cell division cycle 25 homolog C (S. pombe) | 2,23E-03 | 1,512 |
| Ncapd2 | non-SMC condensin I complex, subunit D2 | 3,54E-03 | 1,498 |
| Mcm7 | minichromosome maintenance deficient 7 (S. cerevisiae) | 1,29E-03 | 1,483 |
| Melk | maternal embryonic leucine zipper kinase | 2,43E-03 | 1,482 |
| Kif18a | kinesin family member 18A | 4,97E-03 | 1,477 |
| Cdt1 | chromatin licensing and DNA replication factor 1 | 3,54E-03 | 1,475 |
| Kif22 | kinesin family member 22 | 3,07E-03 | 1,461 |
| Ndc80 | NDC80 homolog, kinetochore complex component (S. cerevisiae) | 1,08E-02 | 1,448 |
| Sgol1 | shugoshin-like 1 (S. pombe) | 3,37E-03 | 1,445 |
| Mcm3 | minichromosome maintenance deficient 3 (S. cerevisiae) | 1,61E-02 | 1,444 |
| Clspn | claspin homolog (Xenopus laevis) | 1,14E-03 | 1,435 |
| Mcm8 | minichromosome maintenance deficient 8 (S. cerevisiae) | 1,30E-03 | 1,433 |
| Dbf4 | DBF4 homolog (S. cerevisiae) | 2,40E-03 | 1,432 |
| Chaf1b | chromatin assembly factor 1, subunit B (p60) | 7,67E-03 | 1,424 |
| Cdca5 | cell division cycle associated 5 | 5,22E-03 | 1,416 |
| Cdc6 | cell division cycle 6 homolog (S. cerevisiae) | 3,58E-04 | 1,413 |
| Uhrf1 | ubiquitin-like, containing PHD and RING finger domains, 1 | 5,58E-03 | 1,404 |
| Aaas | achalasia, adrenocortical insufficiency, alacrimia | 2,29E-03 | 1,401 |
| Reg3g | regenerating islet-derived 3 gamma | 9,00E-04 | 1,397 |
| Kif15 | kinesin family member 15 | 1,39E-03 | 1,380 |
| Cdkn2c | cyclin-dependent kinase inhibitor 2C (p18, inhibits CDK4) | 1,34E-03 | 1,372 |
| Cenph | centromere protein H | 1,73E-02 | 1,371 |
| Tacc2 | transforming, acidic coiled-coil containing protein 2 | 4,75E-05 | 1,371 |
| Plk4 | polo-like kinase 4 (Drosophila) | 2,50E-03 | 1,364 |
| Fam64a | family with sequence similarity 64, member A | 1,35E-02 | 1,363 |
| Dsn1 | DSN1, MIND kinetochore complex component, homolog (S. cerevisiae) | 1,15E-02 | 1,338 |
| E2f7 | E2F transcription factor 7 | 2,60E-03 | 1,336 |
| Gins1 | GINS complex subunit 1 (Psf1 homolog) | 2,87E-03 | 1,332 |
| Mcm10 | minichromosome maintenance deficient 10 (S. cerevisiae) | 1,07E-02 | 1,327 |
| Mlf1ip | myeloid leukemia factor 1 interacting protein | 6,18E-03 | 1,314 |
| Fam83d | family with sequence similarity 83, member D | 3,64E-03 | 1,305 |
| Arhgef10 | Rho guanine nucleotide exchange factor (GEF) 10 | 8,18E-04 | 1,305 |
| Cdc45 | cell division cycle 45 homolog (S. cerevisiae) | 1,72E-03 | 1,305 |
| Leprel4 | leprecan-like 4 | 1,05E-04 | 1,301 |
| Pkmyt1 | protein kinase, membrane associated tyrosine/threonine 1 | 1,12E-03 | 1,298 |
| Mcm4 | minichromosome maintenance deficient 4 homolog (S. cerevisiae) | 2,35E-02 | 1,295 |
| Cep76 | centrosomal protein 76 | 1,37E-03 | 1,292 |
| Cdk5rap3 | CDK5 regulatory subunit associated protein 3 | 5,05E-05 | 1,284 |
| Espl1 | extra spindle poles-like 1 (S. cerevisiae) | 9,53E-03 | 1,268 |
| Haus1 | HAUS augmin-like complex, subunit 1 | 4,14E-02 | 1,264 |
| Cenpp | centromere protein P | 2,28E-02 | 1,261 |
| Gps1 | G protein pathway suppressor 1 | 3,58E-04 | 1,259 |
| Haus4 | HAUS augmin-like complex, subunit 4 | 9,29E-03 | 1,250 |
| Rbl1 | retinoblastoma-like 1 (p107) | 9,66E-03 | 1,246 |
| Cep55 | centrosomal protein 55 | 1,31E-02 | 1,242 |
| Rint1 | RAD50 interactor 1 | 3,69E-03 | 1,237 |
| Incenp | inner centromere protein | 2,70E-02 | 1,235 |
| Usp2 | ubiquitin specific peptidase 2 | 3,02E-04 | 1,234 |
| Nsl1 | NSL1, MIND kinetochore complex component, homolog (S. cerevisiae) | 5,69E-03 | 1,233 |
| Jtb | jumping translocation breakpoint | 6,52E-05 | 1,233 |
| E2f2 | E2F transcription factor 2 | 3,18E-03 | 1,228 |
| Pmf1 | polyamine-modulated factor 1 | 4,32E-03 | 1,226 |
| Sept11 | septin 11 | 8,07E-05 | 1,226 |
| Cdc25a | cell division cycle 25 homolog A (S. pombe) | 6,49E-03 | 1,219 |
| Smc4 | structural maintenance of chromosomes 4 | 4,04E-02 | 1,217 |
| Nudcd2 | NudC domain containing 2 | 5,56E-03 | 1,213 |
| Poc1a | POC1 centriolar protein homolog A (Chlamydomonas) | 3,76E-03 | 1,206 |
| Chaf1a | chromatin assembly factor 1, subunit A (p150) | 2,94E-02 | 1,205 |
| Btrc | beta-transducin repeat containing protein | 3,73E-06 | -1,203 |
| Syce2 | synaptonemal complex central element protein 2 | 1,26E-03 | -1,205 |
| Cspp1 | centrosome and spindle pole associated protein 1 | 6,02E-03 | -1,208 |
| Ep300 | E1A binding protein p300 | 1,77E-03 | -1,209 |
| Foxn3 | forkhead box N3 | 9,61E-04 | -1,219 |
| Scaper | S phase cyclin A-associated protein in the ER | 3,77E-03 | -1,219 |
| Ccnt2 | cyclin T2 | 4,53E-02 | -1,225 |
| Cdk19 | cyclin-dependent kinase 19 | 1,39E-02 | -1,280 |
| Chd7 | chromodomain helicase DNA binding protein 7 | 2,84E-03 | -1,301 |
| Ttc28 | tetratricopeptide repeat domain 28 | 1,85E-03 | -1,354 |
| Phf16 | PHD finger protein 16 | 4,22E-04 | -1,366 |
| Klhl13 | kelch-like 13 (Drosophila) | 5,42E-04 | -1,402 |
| **DNA repair/DNA recombination/DNA replication** | | | |
| Asf1b | ASF1 anti-silencing function 1 homolog B (S. cerevisiae) | 2,65E-04 | 2,496 |
| Pole | polymerase (DNA directed), epsilon | 5,42E-04 | 1,896 |
| Fignl1 | fidgetin-like 1 | 9,37E-05 | 1,846 |
| Fen1 | flap structure specific endonuclease 1 | 6,81E-04 | 1,680 |
| Neil3 | nei like 3 (E. coli) | 3,80E-03 | 1,627 |
| Rrm1 | ribonucleotide reductase M1 | 2,16E-03 | 1,465 |
| Gen1 | Gen homolog 1, endonuclease (Drosophila) | 1,08E-03 | 1,463 |
| Pole2 | polymerase (DNA directed), epsilon 2 (p59 subunit) | 7,74E-04 | 1,448 |
| Fancd2 | Fanconi anemia, complementation group D2 | 5,10E-03 | 1,442 |
| Fancb | Fanconi anemia, complementation group B | 2,60E-03 | 1,440 |
| Brca1 | breast cancer 1 | 2,57E-03 | 1,437 |
| Rad18 | RAD18 homolog (S. cerevisiae) | 6,90E-04 | 1,437 |
| Rpa2 | replication protein A2 | 3,57E-04 | 1,385 |
| Pola2 | polymerase (DNA directed), alpha 2 | 2,00E-03 | 1,384 |
| Rfc4 | replication factor C (activator 1) 4 | 3,85E-03 | 1,370 |
| Gins2 | GINS complex subunit 2 (Psf2 homolog) | 5,51E-03 | 1,361 |
| Brip1 | BRCA1 interacting protein C-terminal helicase 1 | 1,33E-03 | 1,351 |
| Exo1 | exonuclease 1 | 4,88E-03 | 1,340 |
| Rad51ap1 | RAD51 associated protein 1 | 8,53E-04 | 1,332 |
| Pold2 | polymerase (DNA directed), delta 2, regulatory subunit | 7,41E-03 | 1,315 |
| Fanci | Fanconi anemia, complementation group I | 8,17E-03 | 1,314 |
| Hmgb2 | high mobility group box 2 | 3,13E-02 | 1,299 |
| Rexo2 | REX2, RNA exonuclease 2 homolog (S. cerevisiae) | 6,67E-04 | 1,282 |
| Rfc5 | replication factor C (activator 1) 5 | 3,47E-03 | 1,281 |
| Rad54b | RAD54 homolog B (S. cerevisiae) | 6,81E-03 | 1,280 |
| Fanca | Fanconi anemia, complementation group A | 1,53E-02 | 1,272 |
| Topbp1 | topoisomerase (DNA) II binding protein 1 | 3,91E-03 | 1,271 |
| Dna2 | DNA replication helicase 2 homolog (yeast) | 1,20E-02 | 1,236 |
| Lig1 | ligase I, DNA, ATP-dependent | 2,74E-02 | 1,230 |
| Brca2 | breast cancer 2 | 7,71E-03 | 1,222 |
| Poll | polymerase (DNA directed), lambda | 1,34E-02 | 1,207 |
| Nfkbil2 | nuclear factor of kappa light polypeptide gene enhancer In B-Cells Inhibitor-Like 2 | 7,75E-03 | 1,207 |
| Ung | uracil DNA glycosylase | 4,30E-03 | 1,207 |
| Rad9b | RAD9 homolog B (S. cerevisiae) | 1,30E-03 | -1,215 |
| Mns1 | meiosis-specific nuclear structural protein 1 | 9,08E-04 | -1,239 |
| Rdm1 | RAD52 motif 1 | 9,41E-03 | -1,243 |
| Dclre1c | DNA cross-link repair 1C, PSO2 homolog (S. cerevisiae) | 3,25E-03 | -1,246 |
| Rev1 | REV1 homolog (S. cerevisiae) | 9,45E-04 | -1,249 |
| Cdc14b | CDC14 cell division cycle 14 homolog B (S. cerevisiae) | 1,51E-04 | -1,276 |
| **Nucleosome assembly** | | | |
| Hist1h2bb | histone cluster 1, H2bb | 3,03E-02 | 1,430 |
| Hist1h1b | histone cluster 1, H1b | 1,15E-02 | 1,409 |
| Hirip3 | HIRA interacting protein 3 | 3,70E-03 | 1,338 |
| Hist2h3b | histone cluster 2, H3b | 3,78E-04 | 1,332 |
| Hist1h3c | histone cluster 1, H3c | 1,81E-04 | 1,328 |
| Hist1h3b | histone cluster 1, H3b | 1,57E-04 | 1,323 |
| Hist1h3i | histone cluster 1, H3i | 3,21E-04 | 1,323 |
| Hist1h3e | histone cluster 1, H3e | 2,05E-04 | 1,321 |
| Hist1h3g | histone cluster 1, H3g | 2,21E-04 | 1,320 |
| Hist1h3d | histone cluster 1, H3d | 2,45E-04 | 1,314 |
| Hist1h3h | histone cluster 1, H3h | 2,00E-04 | 1,311 |
| Hist2h2bb | histone cluster 2, H2bb | 7,66E-03 | 1,272 |
| Hist1h1a | histone cluster 1, H1a | 2,98E-02 | 1,271 |
| Hist1h2an | histone cluster 1, H2an | 5,12E-03 | 1,224 |
| Hist1h2ao | histone cluster 1, H2ao | 2,72E-03 | 1,223 |
| Hist1h2af | histone cluster 1, H2af | 3,49E-03 | 1,220 |
| Hist1h2ai | histone cluster 1, H2ai | 2,57E-03 | 1,215 |
| Hist1h2ah | histone cluster 1, H2ah | 3,35E-03 | 1,214 |
| Hist1h2ae | histone cluster 1, H2ae | 8,91E-03 | 1,208 |
| Hist1h4j | histone cluster 1, H4j | 1,60E-02 | -1,200 |
| Hist2h2be | histone cluster 2, H2be | 3,00E-04 | -1,250 |
| **Epigenic regulation** | | | |
| Esco2 | establishment of cohesion 1 homolog 2 (S. cerevisiae) | 2,05E-03 | 1,908 |
| Smyd2 | SET and MYND domain containing 2 | 9,34E-05 | 1,208 |
| Mll5 | myeloid/lymphoid or mixed-lineage leukemia 5 | 8,79E-04 | -1,205 |
| Phc3 | polyhomeotic-like 3 (Drosophila) | 8,08E-03 | -1,211 |
| Tet3 | tet oncogene family member 3 | 2,24E-02 | -1,218 |
| Kdm6b | KDM1 lysine (K)-specific demethylase 6B | 9,64E-04 | -1,218 |
| Dnmt3a | DNA methyltransferase 3A | 5,35E-03 | -1,224 |
| Mll3 | myeloid/lymphoid or mixed-lineage leukemia 3 | 2,56E-02 | -1,248 |
| Apobec3 | apolipoprotein B mRNA editing enzyme, catalytic polypeptide like-3 | 6,72E-04 | -1,259 |
| Cbx7 | chromobox homolog 7 | 3,58E-03 | -1,302 |
| A1cf | APOBEC1 complementation factor | 2,46E-03 | -1,315 |
| Tet2 | tet oncogene family member 2 | 4,75E-03 | -1,340 |
| Phf15 | PHD finger protein 15 | 2,67E-03 | -1,376 |
| Tet1 | tet oncogene 1 | 6,11E-03 | -1,465 |
| **Apoptosis** | | | |
| Dapl1 | death associated protein-like 1 | 2,88E-06 | 2,186 |
| Myc | myelocytomatosis oncogene | 6,25E-03 | 1,973 |
| Nupr1 | nuclear protein 1 | 1,25E-03 | 1,709 |
| Fam167a | family with sequence similarity 167, member A | 1,86E-03 | 1,551 |
| Stk17b | serine/threonine kinase 17b (apoptosis-inducing) | 3,59E-04 | 1,418 |
| Birc5 | baculoviral IAP repeat-containing 5 | 2,86E-03 | 1,404 |
| Clptm1l | CLPTM1-like | 1,51E-04 | 1,396 |
| Tpd52l1 | tumor protein D52-like 1 | 4,64E-04 | 1,352 |
| Plekhf1 | pleckstrin homology domain containing, family F (with FYVE domain) member 1 | 5,26E-04 | 1,298 |
| Perp | PERP, TP53 apoptosis effector | 5,65E-03 | 1,281 |
| Lgals12 | lectin, galactose binding, soluble 12 | 4,20E-03 | 1,273 |
| Atp6v1g2 | ATPase, H+ transporting, lysosomal V1 subunit G2 | 1,79E-04 | 1,271 |
| Cd38 | CD38 antigen | 6,77E-03 | 1,265 |
| Krt18 | keratin 18 | 9,37E-05 | 1,256 |
| Tmbim4 | transmembrane BAX inhibitor motif containing 4 | 1,13E-04 | 1,253 |
| Clu | clusterin | 1,49E-03 | 1,253 |
| Ptrh2 | peptidyl-tRNA hydrolase 2 | 4,00E-03 | 1,237 |
| Irak3 | interleukin-1 receptor-associated kinase 3 | 8,51E-03 | 1,235 |
| Chek2 | CHK2 checkpoint homolog (S. pombe) | 5,30E-04 | 1,233 |
| Fam82a2 | family with sequence similarity 82, member A2 | 4,82E-05 | 1,218 |
| Aven | apoptosis, caspase activation inhibitor | 8,67E-04 | 1,216 |
| Hyou1 | hypoxia up-regulated 1 | 2,09E-05 | 1,215 |
| Unc13b | unc-13 homolog B (C. elegans) | 2,53E-04 | 1,214 |
| Parl | presenilin associated, rhomboid-like | 1,70E-03 | 1,203 |
| Faim2 | Fas apoptotic inhibitory molecule 2 | 4,41E-02 | -1,202 |
| Pdcd7 | programmed cell death 7 | 2,28E-02 | -1,210 |
| Plagl2 | pleiomorphic adenoma gene-like 2 | 1,08E-03 | -1,218 |
| Robo1 | roundabout homolog 1 (Drosophila) | 2,46E-03 | -1,232 |
| Ank2 | ankyrin 2, brain | 7,87E-03 | -1,236 |
| Rnf122 | ring finger protein 122 | 1,69E-02 | -1,245 |
| Syngap1 | synaptic Ras GTPase activating protein 1 homolog (rat) | 1,32E-03 | -1,252 |
| Olfm4 | olfactomedin 4 | 1,09E-02 | -1,269 |
| Robo2 | roundabout homolog 2 (Drosophila) | 4,73E-03 | -1,291 |
| Bcl2l11 | BCL2-like 11 (apoptosis facilitator) | 9,71E-04 | -1,321 |
| Amigo2 | adhesion molecule with Ig like domain 2 | 5,71E-03 | -1,322 |
| Serpinb9 | serine (or cysteine) peptidase inhibitor, clade B, member 9 | 9,39E-03 | -1,332 |
| Aatk | apoptosis-associated tyrosine kinase | 6,82E-04 | -1,333 |
| Pycard | PYD and CARD domain containing | 3,13E-04 | -1,336 |
| Eef1a2 | eukaryotic translation elongation factor 1 alpha 2 | 7,31E-03 | -1,341 |
| Higd1a | HIG1 domain family, member 1A | 2,24E-02 | -1,344 |
| Trim35 | tripartite motif-containing 35 | 1,92E-05 | -1,349 |
| Unc5c | unc-5 homolog C (C. elegans) | 9,24E-03 | -1,402 |
| Kcnip3 | Kv channel interacting protein 3, calsenilin | 1,01E-02 | -1,419 |
| Peg10 | paternally expressed 10 | 5,84E-03 | -1,674 |
| Vip | vasoactive intestinal polypeptide | 2,05E-02 | -1,828 |
| **Oxidative stress/DNA damage response** | | | |
| Gpx2 | glutathione peroxidase 2 | 1,79E-05 | 2,070 |
| Osgin1 | oxidative stress induced growth inhibitor 1 | 5,21E-04 | 1,501 |
| Chek1 | checkpoint kinase 1 homolog (S. pombe) | 6,72E-04 | 1,475 |
| Prdx4 | peroxiredoxin 4 | 1,34E-03 | 1,469 |
| Tacc3 | transforming, acidic coiled-coil containing protein 3 | 1,57E-03 | 1,407 |
| Gtse1 | G two S phase expressed protein 1 | 8,59E-03 | 1,319 |
| Pon2 | paraoxonase 2 | 5,80E-05 | 1,284 |
| Tiparp | TCDD-inducible poly(ADP-ribose) polymerase | 8,02E-04 | 1,269 |
| Ehd2 | EH-domain containing 2 | 1,47E-02 | 1,256 |
| Atox1 | ATX1 (antioxidant protein 1) homolog 1 (yeast) | 5,42E-04 | 1,253 |
| Sp100 | nuclear antigen Sp100 | 1,97E-02 | 1,234 |
| Gpx1 | glutathione peroxidase 1 | 3,97E-03 | 1,230 |
| Ppp2r5c | protein phosphatase 2, regulatory subunit B (B56), gamma | 1,73E-06 | 1,230 |
| Phlda3 | pleckstrin homology-like domain, family A, member 3 | 1,45E-02 | 1,203 |
| Gpx8 | glutathione peroxidase 8 (putative) | 2,55E-02 | 1,201 |
| Txndc11 | thioredoxin domain containing 11 | 1,26E-03 | 1,200 |
| Pxdn | peroxidasin homolog (Drosophila) | 2,06E-02 | -1,235 |
| Pot1b | protection of telomeres 1B | 5,95E-03 | -1,239 |
| Nfe2l2 | nuclear factor, erythroid derived 2, like 2 | 8,84E-05 | -1,250 |
| **Transcription factors** | | | |
| Tcf19 | transcription factor 19 | 1,75E-04 | 1,956 |
| Mybl1 | myeloblastosis oncogene-like 1 | 3,96E-03 | 1,472 |
| Etv5 | ets variant gene 5 | 1,24E-02 | 1,335 |
| Tcf7l1 | transcription factor 7-like 1 (T-cell specific, HMG box) | 8,87E-03 | 1,264 |
| Bach2 | BTB and CNC homology 2 | 1,58E-03 | 1,250 |
| Stat5b | signal transducer and activator of transcription 5B | 4,74E-03 | -1,204 |
| Smad2 | MAD homolog 2 (Drosophila) | 1,75E-05 | -1,209 |
| Hsf4 | heat shock transcription factor 4 | 4,36E-03 | -1,216 |
| Nfat5 | nuclear factor of activated T-cells 5 | 7,93E-03 | -1,233 |
| Erf | Ets2 repressor factor | 1,41E-03 | -1,238 |
| Cux2 | cut-like homeobox 2 | 4,76E-03 | -1,242 |
| Arid3b | AT rich interactive domain 3B (BRIGHT-like) | 3,40E-03 | -1,243 |
| Prdm5 | PR domain containing 5 | 3,35E-03 | -1,245 |
| Prdm2 | PR domain containing 2, with ZNF domain | 1,86E-03 | -1,268 |
| Dach1 | dachshund 1 (Drosophila) | 9,45E-04 | -1,269 |
| Mnx1 | motor neuron and pancreas homeobox 1 | 1,79E-02 | -1,274 |
| Egr1 | early growth response 1 | 4,36E-02 | -1,287 |
| Arnt2 | aryl hydrocarbon receptor nuclear translocator 2 | 1,40E-03 | -1,308 |
| Elf4 | E74-like factor 4 (ets domain transcription factor) | 3,92E-03 | -1,314 |
| Hivep3 | human immunodeficiency virus type I enhancer binding protein 3 | 5,66E-04 | -1,335 |
| Per3 | period homolog 3 (Drosophila) | 9,22E-03 | -1,403 |
| Arx | aristaless related homeobox | 9,34E-05 | -1,405 |
| Neurog3 | neurogenin 3 | 6,24E-03 | -1,411 |
| Gtf3c2 | general transcription factor IIIC, polypeptide 2, beta 110 kDa | 3,57E-02 | -1,417 |
| Hhex | hematopoietically expressed homeobox | 1,48E-03 | -1,450 |
| Fos | FBJ osteosarcoma oncogene | 4,68E-02 | -1,481 |
| Plag1 | pleiomorphic adenoma gene 1 | 1,97E-03 | -1,498 |
| Pou3f4 | POU domain, class 3, transcription factor 4 | 5,30E-04 | -1,537 |
| Mafb | v-maf musculoaponeurotic fibrosarcoma oncogene homolog B | 4,58E-03 | -1,607 |
| Fosb | FBJ osteosarcoma oncogene B | 2,94E-02 | -1,789 |
| **Nuclear receptor and related proteins** | | | |
| Nr4a2 | nuclear receptor subfamily 4, group A, member 2 | 3,57E-04 | 1,450 |
| Ncoa1 | nuclear receptor coactivator 1 | 1,33E-03 | -1,202 |
| Nr6a1 | nuclear receptor subfamily 6, group A, member 1 | 3,58E-03 | -1,252 |
| Nr3c2 | nuclear receptor subfamily 3, group C, member 2 | 5,60E-03 | -1,268 |
| Thra | thyroid hormone receptor alpha | 4,52E-04 | -1,365 |
| Ccdc62 | coiled-coil domain containing 62 | 1,31E-03 | -1,385 |
| **Transcription regulation/alternative splicing** | | | |
| Rnase1 | ribonuclease, RNase A family, 1 (pancreatic) | 4,05E-02 | 1,725 |
| Atad2 | ATPase family, AAA domain containing 2 | 9,39E-05 | 1,543 |
| Lmo1 | LIM domain only 1 | 8,49E-05 | 1,536 |
| Zcchc12 | zinc finger, CCHC domain containing 12 | 3,52E-04 | 1,534 |
| Zfp367 | zinc finger protein 367 | 1,09E-03 | 1,507 |
| Ldb2 | LIM domain binding 2 | 1,40E-03 | 1,374 |
| Carhsp1 | calcium regulated heat stable protein 1 | 1,27E-03 | 1,360 |
| Ezh2 | enhancer of zeste homolog 2 (Drosophila) | 7,11E-04 | 1,354 |
| Bhlha15 | basic helix-loop-helix family, member a15 | 1,35E-02 | 1,325 |
| Wdhd1 | WD repeat and HMG-box DNA binding protein 1 | 1,33E-02 | 1,310 |
| Parn | poly(A)-specific ribonuclease (deadenylation nuclease) | 2,69E-04 | 1,309 |
| Dom3z | DOM-3 homolog Z (C. elegans) | 3,35E-03 | 1,303 |
| Gemin6 | gem (nuclear organelle) associated protein 6 | 1,20E-03 | 1,301 |
| Mbnl3 | muscleblind-like 3 (Drosophila) | 3,87E-03 | 1,296 |
| Srp9 | signal recognition particle 9 | 5,77E-05 | 1,282 |
| Rnaseh2c | ribonuclease H2, subunit C | 2,12E-03 | 1,280 |
| Lmcd1 | LIM and cysteine-rich domains 1 | 3,33E-03 | 1,264 |
| Armcx3 | armadillo repeat containing, X-linked 3 | 2,01E-03 | 1,217 |
| Polr1d | polymerase (RNA) I polypeptide D | 1,32E-02 | 1,201 |
| Taf1a | TATA box binding protein (Tbp)-associated factor, RNA polymerase I, A, 48kDa | 9,83E-04 | -1,202 |
| Ppargc1b | peroxisome proliferative activated receptor, gamma, coactivator 1 beta | 2,84E-03 | -1,203 |
| Zfml | zinc finger, matrin-like | 2,40E-03 | -1,203 |
| Zfp192 | zinc finger protein 192 | 1,62E-02 | -1,203 |
| Cnot6 | CCR4-NOT transcription complex, subunit 6 | 2,05E-04 | -1,204 |
| Zbtb3 | zinc finger and BTB domain containing 3 | 3,57E-03 | -1,204 |
| Ssbp2 | single-stranded DNA binding protein 2 | 1,65E-03 | -1,204 |
| Zfp160 | zinc finger protein 160 | 1,61E-03 | -1,207 |
| Ebf4 | early B-cell factor 4 | 5,75E-04 | -1,207 |
| Zfp873 | zinc finger protein 873 | 8,10E-03 | -1,208 |
| Zfp553 | zinc finger protein 553 | 6,09E-04 | -1,212 |
| Elp4 | elongation protein 4 homolog (S. cerevisiae) | 2,60E-03 | -1,212 |
| Zfp28 | zinc finger protein 28 | 5,80E-03 | -1,212 |
| Mov10 | Moloney leukemia virus 10 | 1,79E-03 | -1,214 |
| Luc7l3 | LUC7-like 3 (S. cerevisiae) | 8,93E-03 | -1,217 |
| Fryl | furry homolog-like (Drosophila) | 1,07E-03 | -1,218 |
| Ewsr1 | Ewing sarcoma breakpoint region 1 | 4,29E-03 | -1,219 |
| Chd3 | chromodomain helicase DNA binding protein 3 | 1,18E-03 | -1,221 |
| Zfp420 | zinc finger protein 420 | 7,40E-03 | -1,222 |
| Zfp454 | zinc finger protein 454 | 1,13E-03 | -1,222 |
| Zfp287 | zinc finger protein 287 | 8,03E-03 | -1,225 |
| Zfp26 | zinc finger protein 26 | 4,23E-03 | -1,225 |
| Basp1 | brain abundant, membrane attached signal protein 1 | 3,89E-02 | -1,227 |
| Patz1 | POZ (BTB) and AT hook containing zinc finger 1 | 2,25E-04 | -1,228 |
| Mcts2 | malignant T cell amplified sequence 2 | 4,26E-02 | -1,231 |
| Rbm27 | RNA binding motif protein 27 | 8,28E-06 | -1,236 |
| Gcfc1 | GC-rich sequence DNA-binding factor 1 | 3,69E-04 | -1,237 |
| Tef | thyrotroph embryonic factor | 1,57E-02 | -1,240 |
| Zfp264 | zinc finger protein 264 | 4,34E-04 | -1,240 |
| Zfp300 | zinc finger protein 300 | 6,05E-03 | -1,243 |
| Gm14420 | predicted gene 14420 | 4,71E-02 | -1,245 |
| Zfp709 | zinc finger protein 709 | 1,21E-02 | -1,248 |
| Zfp2 | zinc finger protein 2 | 5,70E-04 | -1,252 |
| Gm4979 | predicted gene 4979 | 6,18E-04 | -1,252 |
| Zfp788 | zinc finger protein 788 | 1,45E-03 | -1,254 |
| Smarca1 | SWI/SNF related, matrix associated, actin dependent regulator of chromatin, subfamily a, member 1 | 1,36E-02 | -1,257 |
| Ankrd12 | ankyrin repeat domain 12 | 2,72E-03 | -1,261 |
| Chd6 | chromodomain helicase DNA binding protein 6 | 3,68E-03 | -1,262 |
| Zfp595 | zinc finger protein 595 | 4,50E-03 | -1,267 |
| Zfp467 | zinc finger protein 467 | 2,83E-03 | -1,272 |
| Gm13051 | predicted gene 13051 | 8,23E-04 | -1,272 |
| Zfp618 | zinc fingerprotein 618 | 1,71E-03 | -1,277 |
| Zfp455 | zinc finger protein 455 | 8,80E-04 | -1,277 |
| Ikzf4 | IKAROS family zinc finger 4 | 7,82E-03 | -1,281 |
| Prpf39 | PRP39 pre-mRNA processing factor 39 homolog (yeast) | 6,03E-03 | -1,282 |
| Zfp72 | zinc finger protein 72 | 5,92E-03 | -1,284 |
| Pou6f2 | POU domain, class 6, transcription factor 2 | 3,51E-02 | -1,287 |
| Sp4 | trans-acting transcription factor 4 | 2,75E-03 | -1,294 |
| Srsf7 | serine/arginine-rich splicing factor 7 | 4,15E-05 | -1,296 |
| Zfp317 | zinc finger protein 317 | 1,45E-03 | -1,301 |
| Clk1 | CDC-like kinase 1 | 2,82E-02 | -1,314 |
| Zkscan16 | zinc finger with KRAB and SCAN domains 16 | 1,43E-02 | -1,324 |
| Irx1 | Iroquois related homeobox 1 (Drosophila) | 1,56E-02 | -1,330 |
| Mll1 | myeloid/lymphoid or mixed-lineage leukemia 1 | 5,64E-03 | -1,342 |
| Zbtb20 | zinc finger and BTB domain containing 20 | 1,01E-03 | -1,342 |
| Camta1 | calmodulin binding transcription activator 1 | 1,04E-05 | -1,343 |
| Klhl3 | kelch-like 3 (Drosophila) | 1,13E-03 | -1,345 |
| Etohi1 | ethanol induced 1 | 2,19E-02 | -1,361 |
| Tra2a | transformer 2 alpha homolog (Drosophila) | 4,98E-03 | -1,362 |
| Zfp583 | zinc finger protein 583 | 5,82E-05 | -1,389 |
| Zfp398 | zinc finger protein 398 | 2,48E-04 | -1,394 |
| Bhlhe41 | basic helix-loop-helix family, member e41 | 1,61E-04 | -1,395 |
| Rcor2 | REST corepressor 2 | 1,39E-03 | -1,448 |
| Gas5 | growth arrest specific 5 | 9,65E-03 | -1,460 |
| Mamld1 | mastermind-like domain containing 1 | 3,90E-04 | -1,529 |
| Dbp | D site albumin promoter binding protein | 9,03E-03 | -1,547 |
| Zim1 | zinc finger, imprinted 1 | 3,09E-04 | -1,603 |
| Msi1 | Musashi homolog 1(Drosophila) | 4,34E-04 | -1,628 |
| **Protein synthesis/translation regulation/protein folding/endoplasmic reticulum stress** | | | |
| Derl3 | Der1-like domain family, member 3 | 1,00E-05 | 2,508 |
| Kdelr3 | KDEL (Lys-Asp-Glu-Leu) endoplasmic reticulum protein retention receptor 3 | 5,93E-05 | 2,387 |
| Erp27 | endoplasmic reticulum protein 27 | 4,94E-02 | 1,939 |
| Fkbp11 | FK506 binding protein 11 | 1,37E-05 | 1,549 |
| Sec11c | SEC11 homolog C (S. cerevisiae) | 6,27E-05 | 1,464 |
| Dnajb11 | DnaJ (Hsp40) homolog, subfamily B, member 11 | 4,64E-04 | 1,455 |
| Spcs1 | signal peptidase complex subunit 1 homolog | 7,08E-04 | 1,436 |
| Prkcsh | protein kinase C substrate 80K-H | 5,53E-05 | 1,414 |
| Qsox1 | quiescin Q6 sulfhydryl oxidase 1 | 1,04E-04 | 1,410 |
| Pdrg1 | p53 and DNA damage regulated 1 | 1,03E-04 | 1,382 |
| Mrps12 | mitochondrial ribosomal protein S12 | 4,52E-04 | 1,350 |
| Sel1l | sel-1 suppressor of lin-12-like (C. elegans) | 1,61E-04 | 1,344 |
| Edem2 | ER degradation enhancer, mannosidase alpha-like 2 | 1,13E-04 | 1,341 |
| Golm1 | golgi membrane protein 1 | 2,27E-04 | 1,328 |
| Ppib | peptidylprolyl isomerase B | 3,02E-04 | 1,325 |
| Pacrg | PARK2 co-regulated | 1,24E-03 | 1,324 |
| Syvn1 | synovial apoptosis inhibitor 1, synoviolin | 1,66E-03 | 1,318 |
| Hars | histidyl-tRNA synthetase | 5,41E-04 | 1,315 |
| Edem1 | ER degradation enhancer, mannosidase alpha-like 1 | 5,72E-05 | 1,314 |
| Paip2b | poly(A) binding protein interacting protein 2B | 9,30E-06 | 1,311 |
| Tmem48 | transmembrane protein 48 | 9,92E-03 | 1,307 |
| Ppic | peptidylprolyl isomerase C | 3,30E-02 | 1,303 |
| Tor2a | torsin family 2, member A | 1,93E-04 | 1,299 |
| Hspb6 | heat shock protein, alpha-crystallin-related, B6 | 1,18E-02 | 1,299 |
| Nucb1 | nucleobindin 1 | 2,11E-05 | 1,297 |
| Pdia6 | protein disulfide isomerase associated 6 | 1,81E-04 | 1,296 |
| Dnajc3 | DnaJ (Hsp40) homolog, subfamily C, member 3 | 3,54E-05 | 1,286 |
| Os9 | amplified in osteosarcoma | 1,75E-05 | 1,280 |
| Pdia4 | protein disulfide isomerase associated 4 | 6,11E-03 | 1,264 |
| Fkbp2 | FK506 binding protein 2 | 3,99E-05 | 1,261 |
| Ptrh1 | peptidyl-tRNA hydrolase 1 homolog (S. cerevisiae) | 4,25E-03 | 1,259 |
| Yif1a | Yip1 interacting factor homolog A (S. cerevisiae) | 4,18E-03 | 1,256 |
| Eif3i | eukaryotic translation initiation factor 3, subunit I | 1,57E-04 | 1,256 |
| Selm | selenoprotein M | 4,66E-04 | 1,255 |
| Pdia5 | protein disulfide isomerase associated 5 | 6,27E-05 | 1,253 |
| Kars | lysyl-tRNA synthetase | 1,74E-04 | 1,250 |
| Prorsd1 | prolyl-tRNA synthetase domain containing 1 | 7,90E-04 | 1,240 |
| Ier3ip1 | immediate early response 3 interacting protein 1 | 1,49E-04 | 1,240 |
| Rps8 | ribosomal protein S8 | 1,23E-03 | 1,236 |
| Eef1g | eukaryotic translation elongation factor 1 gamma | 3,36E-02 | 1,229 |
| Qtrt1 | queuine tRNA-ribosyltransferase 1 | 1,70E-04 | 1,228 |
| Rps14 | ribosomal protein S14 | 2,10E-03 | 1,227 |
| H47 | histocompatibility 47 | 1,62E-04 | 1,226 |
| Arfgap3 | ADP-ribosylation factor GTPase activating protein 3 | 1,18E-04 | 1,225 |
| Eef2k | eukaryotic elongation factor-2 kinase | 3,70E-03 | 1,225 |
| Manf | mesencephalic astrocyte-derived neurotrophic factor | 1,40E-03 | 1,223 |
| Hbs1l | Hbs1-like (S. cerevisiae) | 1,22E-04 | 1,223 |
| Tyw1 | tRNA-yW synthesizing protein 1 homolog (S. cerevisiae) | 2,09E-05 | 1,219 |
| Rps29 | ribosomal protein S29 | 4,48E-04 | 1,219 |
| Rplp1 | ribosomal protein, large, P1 | 1,98E-03 | 1,219 |
| Dnajc10 | DnaJ (Hsp40) homolog, subfamily C, member 10 | 8,10E-04 | 1,217 |
| BB287469 | expressed sequence BB287469 | 2,73E-02 | 1,216 |
| Rnf5 | ring finger protein 5 | 6,20E-03 | 1,215 |
| Ftsj1 | FtsJ homolog 1 (E. coli) | 2,86E-03 | 1,212 |
| Sil1 | endoplasmic reticulum chaperone SIL1 homolog (S. cerevisiae) | 3,41E-04 | 1,212 |
| Dars2 | aspartyl-tRNA synthetase 2 (mitochondrial) | 5,65E-04 | 1,209 |
| Uba52 | ubiquitin A-52 residue ribosomal protein fusion product 1 | 1,26E-04 | 1,207 |
| Ssr3 | signal sequence receptor, gamma | 8,77E-05 | 1,207 |
| Zc3h3 | zinc finger CCCH type containing 3 | 1,14E-02 | -1,219 |
| Rpp25 | ribonuclease P 25 subunit (human) | 2,60E-02 | -1,243 |
| C1ql2 | complement component 1, q subcomponent-like 2 | 1,51E-02 | -1,304 |
| Hspa12a | heat shock protein 12A | 2,90E-03 | -1,313 |
| Rpl23a | ribosomal protein L23a | 7,30E-04 | -1,335 |
| Macrod2 | MACRO domain containing 2 | 7,77E-03 | -1,363 |
| Igf2bp2 | insulin-like growth factor 2 mRNA binding protein 2 | 4,08E-05 | -1,510 |
| Ppil6 | peptidylprolyl isomerase (cyclophilin)-like 6 | 3,34E-05 | -1,779 |
| **Vesicle transport/Protein trafficking** | | | |
| Rab18 | RAB18, member RAS oncogene family | 3,82E-03 | 1,511 |
| Copz2 | coatomer protein complex, subunit zeta 2 | 8,28E-06 | 1,491 |
| Tmed3 | transmembrane emp24 domain containing 3 | 1,02E-05 | 1,453 |
| Sytl1 | synaptotagmin-like 1 | 6,74E-04 | 1,408 |
| Slc18a1 | solute carrier family 18 (vesicular monoamine), member 1 | 4,75E-05 | 1,349 |
| Mcfd2 | multiple coagulation factor deficiency 2 | 2,74E-04 | 1,335 |
| Sec16b | SEC16 homolog B (S. cerevisiae) | 2,15E-04 | 1,319 |
| Ssr4 | signal sequence receptor, delta | 1,71E-05 | 1,314 |
| Srprb | signal recognition particle receptor, B subunit | 1,22E-04 | 1,293 |
| Rab26 | RAB26, member RAS oncogene family | 2,60E-04 | 1,292 |
| Timm17b | translocase of inner mitochondrial membrane 17b | 1,45E-03 | 1,285 |
| Cpne4 | copine IV | 1,16E-02 | 1,272 |
| Gorasp1 | golgi reassembly stacking protein 1 | 3,02E-04 | 1,269 |
| Bloc1s1 | biogenesis of lysosome-related organelles complex-1, subunit 1 | 2,92E-04 | 1,255 |
| Erp29 | endoplasmic reticulum protein 29 | 2,43E-03 | 1,255 |
| Nbas | neuroblastoma amplified sequence | 9,86E-04 | 1,243 |
| Scfd2 | Sec1 family domain containing 2 | 7,89E-04 | 1,233 |
| Rab39b | RAB39B, member RAS oncogene family | 8,17E-03 | 1,232 |
| Ubl4 | ubiquitin-like 4 | 6,29E-05 | 1,229 |
| Dnajc19 | DnaJ (Hsp40) homolog, subfamily C, member 19 | 7,13E-03 | 1,218 |
| Rrbp1 | ribosome binding protein 1 | 7,05E-05 | 1,211 |
| Tmed9 | transmembrane emp24 protein transport domain containing 9 | 5,42E-04 | 1,206 |
| Cog6 | component of oligomeric golgi complex 6 | 1,67E-04 | 1,204 |
| Bet1 | blocked early in transport 1 homolog (S. cerevisiae) | 9,45E-03 | 1,204 |
| Kdelc1 | KDEL (Lys-Asp-Glu-Leu) containing 1 | 7,11E-03 | -1,205 |
| Syt14 | synaptotagmin XIV | 5,16E-04 | -1,210 |
| Cabp7 | calcium binding protein 7 | 8,05E-03 | -1,223 |
| Pclo | piccolo (presynaptic cytomatrix protein) | 2,57E-02 | -1,230 |
| Stx11 | syntaxin 11 | 2,96E-02 | -1,264 |
| Rab3b | RAB3B, member RAS oncogene family | 1,42E-03 | -1,331 |
| Kalrn | kalirin, RhoGEF kinase | 2,01E-04 | -1,515 |
| **Posttranslational modification/ubiquination/glycosylation** | | | |
| Ube2c | ubiquitin-conjugating enzyme E2C | 1,76E-04 | 2,247 |
| Zdhhc22 | zinc finger, DHHC-type containing 22 | 7,74E-03 | 1,873 |
| Otud7a | OTU domain containing 7A | 4,17E-05 | 1,588 |
| Trim59 | tripartite motif-containing 59 | 1,45E-02 | 1,565 |
| Man1a | mannosidase 1, alpha | 2,80E-06 | 1,456 |
| Mogs | mannosyl-oligosaccharide glucosidase | 6,51E-04 | 1,432 |
| Qpctl | glutaminyl-peptide cyclotransferase-like | 8,28E-06 | 1,428 |
| Gyltl1b | glycosyltransferase-like 1B | 1,21E-03 | 1,372 |
| Rnf215 | ring finger protein 215 | 2,69E-05 | 1,363 |
| Bard1 | BRCA1 associated RING domain 1 | 2,15E-03 | 1,346 |
| Gmppb | GDP-mannose pyrophosphorylase B | 5,39E-03 | 1,336 |
| Ube2t | ubiquitin-conjugating enzyme E2T (putative) | 9,96E-04 | 1,302 |
| Uba5 | ubiquitin-like modifier activating enzyme 5 | 2,69E-04 | 1,301 |
| Krtcap2 | keratinocyte associated protein 2 | 1,01E-03 | 1,260 |
| Rpn2 | ribophorin II | 8,28E-06 | 1,256 |
| Trim68 | tripartite motif-containing 68 | 4,42E-03 | 1,252 |
| Asb9 | ankyrin repeat and SOCS box-containing 9 | 1,55E-02 | 1,248 |
| Dhdds | dehydrodolichyl diphosphate synthase | 9,37E-04 | 1,236 |
| Dpagt1 | dolichyl-phosphate (UDP-N-acetylglucosamine) N-acetylglucosaminephosphotransferase 1 (GlcNAc-1-P transferase) | 6,54E-03 | 1,221 |
| Wdyhv1 | WDYHV motif containing 1 | 2,02E-06 | 1,220 |
| Dolk | dolichol kinase | 5,01E-05 | 1,219 |
| Neurl1B | neuralized homolog 1B (Drosophila) | 2,06E-02 | 1,213 |
| Aga | aspartylglucosaminidase | 6,27E-05 | 1,209 |
| Gpaa1 | GPI anchor attachment protein 1 | 3,99E-03 | 1,208 |
| Mpi | mannose phosphate isomerase | 1,20E-03 | 1,208 |
| Ube2s | ubiquitin-conjugating enzyme E2S | 7,00E-04 | 1,205 |
| Zdhhc23 | zinc finger, DHHC domain containing 23 | 2,75E-02 | -1,203 |
| B3gnt8 | UDP-GlcNAc:betaGal beta-1,3-N-acetylglucosaminyltransferase 8 | 8,77E-04 | -1,204 |
| Ttll3 | tubulin tyrosine ligase-like family, member 3 | 3,35E-03 | -1,205 |
| March4 | membrane-associated ring finger (C3HC4) 4 | 7,66E-04 | -1,216 |
| Ttll13 | tubulin tyrosine ligase-like family, member 13 | 4,24E-03 | -1,217 |
| Setd2 | SET domain containing 2 | 2,96E-03 | -1,219 |
| Rnf34 | ring finger protein 34 | 2,04E-03 | -1,224 |
| Pdzrn4 | PDZ domain containing RING finger 4 | 1,03E-02 | -1,230 |
| Unkl | unkempt-like (Drosophila) | 6,25E-03 | -1,230 |
| Uba7 | ubiquitin-like modifier activating enzyme 7 | 2,49E-04 | -1,268 |
| Rimklb | ribosomal modification protein rimK-like family member B | 2,93E-03 | -1,277 |
| Rnf208 | ring finger protein 208 | 1,44E-02 | -1,292 |
| Ube2ql1 | ubiquitin-conjugating enzyme E2Q family-like 1 | 9,50E-04 | -1,316 |
| Mdm4 | transformed mouse 3T3 cell double minute 4 | 2,82E-04 | -1,323 |
| Galnt13 | UDP-N-acetyl-alpha-D-galactosamine: Polypeptide  N-Acetylgalactosaminyltransferase 13 | 2,75E-03 | -1,331 |
| Galntl4 | UDP-N-acetyl-alpha-D-galactosamine: Polypeptide  N-Acetylgalactosaminyltransferase-Like 4 | 5,71E-03 | -1,333 |
| Galnt14 | UDP-N-acetyl-alpha-D-galactosamine: Polypeptide  N-Acetylgalactosaminyltransferase 14 | 1,92E-04 | -1,361 |
| Rbbp6 | retinoblastoma binding protein 6 | 2,80E-06 | -1,423 |
| **Proteosome/lysosome/autophagy/phagocytosis** | | | |
| Wipi1 | WD repeat domain, phosphoinositide interacting 1 | 4,17E-05 | 1,364 |
| Tmem49 | transmembrane protein 49 | 3,85E-04 | 1,334 |
| Atg4a | autophagy-related 4A (yeast) | 3,04E-05 | 1,286 |
| Snca | synuclein, alpha | 2,65E-03 | 1,276 |
| Ctsh | cathepsin H | 3,45E-03 | 1,254 |
| Mfge8 | milk fat globule-EGF factor 8 protein | 2,96E-03 | 1,251 |
| Elmo1 | engulfment and cell motility 1, ced-12 homolog (C. elegans) | 1,13E-04 | 1,244 |
| Tmem9 | transmembrane protein 9 | 1,06E-03 | 1,244 |
| Sh3kbp1 | SH3-domain kinase binding protein 1 | 3,80E-04 | 1,223 |
| Ift88 | intraflagellar transport 88 homolog (Chlamydomonas) | 2,67E-03 | -1,203 |
| Mcoln3 | mucolipin 3 | 4,30E-03 | -1,498 |
| Dnajc13 | DnaJ (Hsp40) homolog, subfamily C, member 13 | 2,72E-03 | -1,260 |
| **Peptidase/protease and related inhibitors** | | | |
| Spink3 | serine peptidase inhibitor, Kazal type 3 | 2,47E-02 | 2,874 |
| Serpina7 | serine (or cysteine) peptidase inhibitor, Clade A (Alpha-1 Antiproteinase, Antitrypsin), Member 7 | 1,54E-04 | 2,635 |
| Prss1 | protease, serine, 1 (trypsin 1) | 3,46E-02 | 1,764 |
| Cela3b | chymotrypsin-like elastase family, member 3B | 2,36E-02 | 1,718 |
| Serpinb1a | serine (or cysteine) peptidase inhibitor, clade B, member 1a | 4,30E-02 | 1,557 |
| Prss2 | protease, serine, 2 | 4,05E-02 | 1,532 |
| Pappa2 | pappalysin 2 | 1,97E-03 | 1,460 |
| Prss23 | protease, serine, 23 | 3,73E-04 | 1,429 |
| Serpini1 | serine (or cysteine) peptidase inhibitor, clade I, member 1 | 1,54E-06 | 1,405 |
| Pamr1 | peptidase domain containing associated with muscle regeneration 1 | 1,73E-06 | 1,386 |
| Ctrb1 | chymotrypsinogen B1 | 1,90E-02 | 1,358 |
| Tmprss4 | transmembrane protease, serine 4 | 1,04E-03 | 1,344 |
| Dpp3 | dipeptidylpeptidase 3 | 8,03E-04 | 1,279 |
| Serpinb8 | serine (or cysteine) peptidase inhibitor, clade B, member 8 | 2,37E-03 | 1,267 |
| Adamts9 | a disintegrin-like and metallopeptidase (reprolysin type) | 5,16E-03 | 1,264 |
| Tmprss2 | transmembrane protease, serine 2 | 3,96E-02 | 1,263 |
| Prss8 | protease, serine, 8 (prostasin) | 4,47E-03 | 1,251 |
| Spg7 | spastic paraplegia 7 homolog (human) | 2,05E-04 | 1,239 |
| Cpa1 | carboxypeptidase A1 | 4,49E-02 | 1,237 |
| C1rl | complement component 1, r subcomponent-like | 1,42E-02 | 1,231 |
| Lxn | latexin | 3,01E-02 | 1,224 |
| Npepl1 | aminopeptidase-like 1 | 2,51E-03 | 1,221 |
| Timp3 | tissue inhibitor of metalloproteinase 3 | 2,51E-02 | 1,215 |
| Hgfac | hepatocyte growth factor activator | 3,19E-03 | 1,213 |
| Tasp1 | taspase, threonine aspartase 1 | 1,57E-03 | 1,210 |
| Tysnd1 | trypsin domain containing 1 | 4,66E-03 | 1,207 |
| Afg3l1 | AFG3(ATPase family gene 3)-like 1 (yeast) | 3,32E-03 | 1,202 |
| Capn9 | calpain 9 | 4,11E-02 | -1,201 |
| Serpind1 | serine (or cysteine) peptidase inhibitor, clade D, (heparin cofactor), member 1 | 1,73E-02 | -1,206 |
| Uspl1 | ubiquitin specific peptidase like 1 | 2,10E-02 | -1,209 |
| Adamts18 | a disintegrin-like and metallopeptidase (reprolysin type 1, motif 18 | 1,85E-02 | -1,220 |
| Agbl4 | ATP/GTP binding protein-like 4 | 1,94E-02 | -1,222 |
| Usp29 | ubiquitin specific peptidase 29 | 1,08E-02 | -1,228 |
| Dpp6 | dipeptidylpeptidase 6 | 7,50E-03 | -1,246 |
| Lonrf1 | LON peptidase N-terminal domain and ring finger 1 | 9,53E-03 | -1,269 |
| Pcsk6 | proprotein convertase subtilisin/kexin type 6 | 5,64E-03 | -1,293 |
| Cpm | carboxypeptidase M | 4,79E-02 | -1,363 |
| Usp11 | ubiquitin specific peptidase 11 | 1,64E-03 | -1,418 |
| Thsd4 | thrombospondin, type I, domain containing 4 | 7,53E-04 | -1,432 |
| Reln | reelin | 1,38E-03 | -1,918 |
| **Cytoskeleton and related proteins** | | | |
| Gas2l3 | growth arrest-specific 2 like 3 | 1,68E-03 | 1,910 |
| Frmd5 | FERM domain containing 5 | 1,35E-04 | 1,636 |
| Kif18b | kinesin family member 18B | 3,96E-03 | 1,599 |
| Diap3 | diaphanous homolog 3 (Drosophila) | 9,38E-04 | 1,428 |
| Acta2 | actin, alpha 2, smooth muscle, aorta | 2,14E-02 | 1,375 |
| Dnahc9 | dynein, axonemal, heavy chain 9 | 5,50E-04 | 1,367 |
| Kif14 | kinesin family member 14 | 5,33E-03 | 1,352 |
| Cd93 | CD93 antigen | 9,32E-04 | 1,326 |
| AI428936 | expressed sequence AI428936 | 4,00E-03 | 1,305 |
| Pak3 | p21 protein (Cdc42/Rac)-activated kinase 3 | 1,48E-04 | 1,294 |
| Sepx1 | selenoprotein X 1 | 1,38E-03 | 1,274 |
| Pdlim1 | PDZ and LIM domain 1 (elfin) | 9,24E-03 | 1,254 |
| Msn | moesin | 3,69E-03 | 1,245 |
| Smtn | smoothelin | 1,45E-03 | 1,242 |
| Trdn | triadin | 5,50E-04 | 1,234 |
| Iqsec2 | IQ motif and Sec7 domain 2 | 1,54E-04 | 1,233 |
| Osbpl3 | oxysterol binding protein-like 3 | 5,07E-03 | 1,228 |
| Csrp1 | cysteine and glycine-rich protein 1 | 6,17E-03 | 1,216 |
| Ckap4 | cytoskeleton-associated protein 4 | 4,32E-03 | 1,209 |
| Coro7 | coronin 7 | 5,84E-03 | 1,208 |
| Tmsb10 | thymosin, beta 10 | 3,10E-02 | 1,200 |
| Atxn7 | ataxin 7 | 1,05E-03 | -1,221 |
| Ccdc141 | coiled-coil domain containing 141 | 4,47E-02 | -1,202 |
| Cep350 | centrosomal protein 350 | 1,88E-02 | -1,205 |
| Cnn3 | calponin 3, acidic // 3 G1\|3 | 1,81E-02 | -1,248 |
| Cobl | cordon-bleu | 1,81E-03 | -1,274 |
| Dnahc2 | dynein, axonemal, heavy chain 2 | 1,87E-04 | -1,237 |
| Dnalc1 | Dnalc1 | 4,52E-04 | -1,232 |
| Dpysl2 | dihydropyrimidinase-like 2 | 1,71E-03 | -1,223 |
| Edn3 | endothelin 3 | 4,30E-03 | -1,570 |
| Eml5 | echinoderm microtubule associated protein like 5 | 3,05E-02 | -1,231 |
| Epb4.1 | erythrocyte protein band 4.1 | 1,70E-03 | -1,207 |
| Epb4.1l3 | erythrocyte protein band 4.1-like 3 | 8,12E-04 | -1,247 |
| Epb4.1l4a | erythrocyte protein band 4.1-like 4a | 1,02E-03 | -1,571 |
| Epb4.9 | erythrocyte protein band 4.9 | 2,72E-03 | -1,207 |
| Ezr | ezrin | 4,03E-04 | -1,227 |
| Fmn2 | formin 2 | 4,19E-04 | -1,253 |
| Kif5a | kinesin family member 5A | 3,87E-03 | -1,239 |
| Kifc3 | kinesin family member C3 | 1,93E-04 | -1,243 |
| Mtap2 | microtubule-associated protein 2 | 5,33E-04 | -1,208 |
| Mtss1 | metastasis suppressor 1 | 1,77E-04 | -1,275 |
| Myo3a | myosin IIIA | 8,05E-05 | -1,646 |
| Myo9a | myosin Ixa | 2,02E-03 | -1,501 |
| Ppp1r12b | protein phosphatase 1, regulatory (inhibitor) subunit 12B | 1,29E-02 | -1,216 |
| Scin | scinderin | 1,43E-03 | -1,224 |
| Sgce | sarcoglycan, epsilon | 4,38E-03 | -1,386 |
| Sntg1 | syntrophin, gamma 1 | 1,68E-02 | -1,213 |
| Syne2 | synaptic nuclear envelope 2 | 4,18E-02 | -1,325 |
| Tenc1 | tensin like C1 domain-containing phosphatase | 3,72E-03 | -1,211 |
| Thsd7a | thrombospondin, type I, domain containing 7A | 8,71E-03 | -1,246 |
| Tmsb15l | thymosin beta 15b like | 1,62E-03 | -1,415 |
| Tnik | TRAF2 and NCK interacting kinase | 1,12E-03 | -1,352 |
| Tppp3 | tubulin polymerization-promoting protein family member 3 | 2,47E-03 | -1,530 |
| Tubb3 | tubulin, beta 3 | 2,21E-02 | -1,234 |
| Wipf2 | WAS/WASL interacting protein family, member 2 | 8,12E-04 | -1,209 |
| **Channels and transporters** | | | |
| Ttyh1 | tweety homolog 1 (Drosophila) | 4,03E-04 | 2,143 |
| Slc2a6 | solute carrier family 2 (facilitated glucose transporter), member 6 | 3,50E-05 | 2,142 |
| Slc17a9 | solute carrier family 17, member 9 | 6,14E-05 | 1,929 |
| Cngb3 | cyclic nucleotide gated channel beta 3 | 3,92E-05 | 1,833 |
| Slc38a10 | solute carrier family 38, member 10 | 5,75E-05 | 1,728 |
| Rhd | Rh blood group, D antigen | 8,15E-05 | 1,703 |
| Car4 | carbonic anhydrase 4 | 1,02E-04 | 1,694 |
| Slco1a5 | solute carrier organic anion transporter family, member 5 | 1,01E-02 | 1,660 |
| Abcb6 | ATP-binding cassette, sub-family B (MDR/TAP), member 6 | 1,37E-05 | 1,624 |
| Trpc4 | transient receptor potential cation channel, subfamily C, member 4 | 1,37E-04 | 1,624 |
| Rbp7 | retinol binding protein 7, cellular | 1,36E-04 | 1,561 |
| Slc39a11 | solute carrier family 39 (metal ion transporter), member 11 | 4,37E-04 | 1,521 |
| Kcnab3 | potassium voltage-gated channel, shaker-related subfamily | 2,17E-04 | 1,472 |
| Kcnk10 | potassium channel, subfamily K, member 10 | 4,18E-04 | 1,448 |
| Slc4a10 | solute carrier family 4, sodium bicarbonate cotransporter, member 10 | 7,30E-06 | 1,420 |
| Slc26a2 | solute carrier family 26 (sulfate transporter), member 2 | 1,43E-04 | 1,411 |
| Kcnh1 | potassium voltage-gated channel, subfamily H (eag-related) | 6,35E-04 | 1,396 |
| Slc46a1 | solute carrier family 46, member 1 | 1,45E-04 | 1,392 |
| Kcnip2 | Kv channel-interacting protein 2 | 3,99E-03 | 1,390 |
| Cacng3 | calcium channel, voltage-dependent, gamma subunit 3 | 2,49E-03 | 1,383 |
| Slc1a5 | solute carrier family 1 (neutral amino acid transporter), member 5 | 1,06E-02 | 1,378 |
| Pex5l | peroxisomal biogenesis factor 5-like | 6,39E-05 | 1,371 |
| Abcc9 | ATP-binding cassette, sub-family C (CFTR/MRP), member 9 | 1,70E-03 | 1,346 |
| Slc35c2 | solute carrier family 35, member C2 | 2,43E-05 | 1,336 |
| Tomm40l | translocase of outer mitochondrial membrane 40 homolog (yeast)-like | 2,24E-05 | 1,321 |
| Kcnk1 | potassium channel, subfamily K, member 1 | 9,14E-05 | 1,313 |
| Slc35f4 | solute carrier family 35, member F4 | 3,69E-03 | 1,311 |
| Slc35b1 | solute carrier family 35, member B1 | 6,27E-05 | 1,301 |
| Slco1a6 | solute carrier organic anion transporter family, member 6 | 4,82E-02 | 1,294 |
| Atp13a2 | ATPase type 13A2 | 4,75E-05 | 1,288 |
| Slc39a10 | solute carrier family 39 (zinc transporter), member 10 | 7,10E-05 | 1,288 |
| Slc12a2 | solute carrier family 12, member 2 | 4,18E-04 | 1,278 |
| Slc39a13 | solute carrier family 39 (metal ion transporter), member 13 | 6,29E-04 | 1,276 |
| Slc6a9 | solute carrier family 6 (neurotransmitter transporter, glycine), member 9 | 2,94E-02 | 1,274 |
| Abca4 | ATP-binding cassette, sub-family A (ABC1), member 4 | 1,38E-03 | 1,272 |
| Slc31a1 | solute carrier family 31, member 1 | 2,84E-04 | 1,264 |
| Tmc6 | transmembrane channel-like gene family 6 | 1,31E-03 | 1,263 |
| Kcnk6 | potassium inwardly-rectifying channel, subfamily K, member 6 | 9,04E-03 | 1,262 |
| Slc25a10 | solute carrier family 25 (mitochondrial carrier; dicarboxylate transporter), member 10 | 7,72E-04 | 1,259 |
| Atp6v0e2 | ATPase, H+ transporting, lysosomal V0 subunit E2 | 1,84E-04 | 1,257 |
| Slc12a4 | solute carrier family 12, member 4 | 7,83E-05 | 1,248 |
| Slc22a2 | solute carrier family 22 (organic cation transporter), member 2 | 1,03E-03 | 1,246 |
| Ano1 | anoctamin 1, calcium activated chloride channel | 2,20E-02 | 1,245 |
| Slc35f1 | solute carrier family 35, member F1 | 4,79E-04 | 1,243 |
| Slc39a7 | solute carrier family 39 (zinc transporter), member 7 | 1,24E-03 | 1,233 |
| Slc30a7 | solute carrier family 30 (zinc transporter), member 7 | 4,52E-04 | 1,231 |
| Slc7a4 | solute carrier family 7 (cationic amino acid transporter), member 4 | 6,96E-03 | 1,226 |
| Slc29a3 | solute carrier family 29 (nucleoside transporters), member 3 | 9,56E-04 | 1,224 |
| Slc41a2 | solute carrier family 41, member 2 | 5,30E-03 | 1,223 |
| Kcnk13 | potassium channel, subfamily K, member 13 | 2,80E-02 | 1,219 |
| Slc27a4 | solute carrier family 27 (fatty acid transporter), member 4 | 1,35E-04 | 1,211 |
| Ipo4 | importin 4 | 7,08E-04 | 1,210 |
| Slc2a13 | solute carrier family 2 (facilitated glucose transporter), member 13 | 7,26E-03 | 1,209 |
| Rag1ap1 | recombination activating gene 1 activating protein 1 | 1,32E-03 | 1,204 |
| Slc7a1 | solute carrier family 7 (cationic amino acid transporter), member 1 | 1,81E-02 | 1,202 |
| Slc6a17 | solute carrier family 6 (neurotransmitter transporter), member 17 | 3,58E-04 | 1,200 |
| Slc41a1 | solute carrier family 41, member 1 | 1,64E-03 | 1,200 |
| Sfxn4 | sideroflexin 4 | 7,85E-03 | -1,209 |
| Unc80 | unc-80 homolog (C. elegans) | 7,44E-03 | -1,210 |
| Atp1b2 | ATPase, Na+/K+ transporting, beta 2 polypeptide | 3,03E-03 | -1,212 |
| Cacna2d2 | calcium channel, voltage-dependent, alpha 2/delta subunit | 4,40E-03 | -1,216 |
| Kcna1 | potassium voltage-gated channel, shaker-related subfamily, member 1 | 4,36E-02 | -1,216 |
| Tmed8 | transmembrane emp24 domain containing 8 | 3,54E-03 | -1,218 |
| Slc44a1 | solute carrier family 44, member 1 | 7,52E-04 | -1,220 |
| Tmem30b | transmembrane protein 30B | 9,38E-04 | -1,221 |
| Cacna1c | calcium channel, voltage-dependent, L type, alpha 1C | 1,01E-02 | -1,222 |
| Rangrf | RAN guanine nucleotide release factor | 1,20E-02 | -1,222 |
| Atp8a2 | ATPase, aminophospholipid transporter-like, class I, type 8A, member 2 | 1,81E-02 | -1,224 |
| Aqp4 | aquaporin 4 | 5,35E-03 | -1,225 |
| Slc12a6 | solute carrier family 12, member 6 | 2,15E-02 | -1,226 |
| Kpna3 | karyopherin (importin) alpha 3 | 9,03E-06 | -1,226 |
| Cacna1a | calcium channel, voltage-dependent, P/Q type, alpha 1A subunit | 3,34E-03 | -1,228 |
| Slc16a7 | solute carrier family 16 (monocarboxylic acid transporters), member 7 | 2,35E-02 | -1,242 |
| Kcnk3 | potassium channel, subfamily K, member 3 | 4,94E-04 | -1,246 |
| Abca8b | ATP-binding cassette, sub-family A (ABC1), member 8b | 2,21E-02 | -1,247 |
| Scnn1a | sodium channel, nonvoltage-gated 1 alpha | 3,34E-02 | -1,250 |
| Gpm6a | glycoprotein m6a | 3,56E-02 | -1,260 |
| Syngr3 | synaptogyrin 3 | 3,22E-03 | -1,264 |
| Atp7a | ATPase, Cu++ transporting, alpha polypeptide | 8,86E-04 | -1,279 |
| Cacng4 | calcium channel, voltage-dependent, gamma subunit 4 | 4,38E-02 | -1,287 |
| Ap1s2 | adaptor-related protein complex 1, sigma 2 subunit | 9,44E-03 | -1,314 |
| Psd | pleckstrin and Sec7 domain containing | 1,35E-03 | -1,314 |
| Trpm5 | transient receptor potential cation channel, subfamily M, member 5 | 2,06E-03 | -1,338 |
| Slc7a2 | solute carrier family 7 (cationic amino acid transporter, Y+ System),Member 2 | 9,34E-04 | -1,351 |
| Kcnj3 | potassium inwardly-rectifying channel, subfamily J, member 3 | 1,26E-02 | -1,371 |
| Sv2b | synaptic vesicle glycoprotein 2 b | 1,39E-03 | -1,395 |
| Nlgn2 | neuroligin 2 | 5,80E-05 | -1,396 |
| Jph3 | junctophilin 3 | 2,05E-04 | -1,406 |
| Kcnj12 | potassium inwardly-rectifying channel, subfamily J, member 12 | 5,51E-03 | -1,409 |
| Kcng3 | potassium voltage-gated channel, subfamily G, member 3 | 2,67E-03 | -1,415 |
| Slc29a4 | solute carrier family 29 (nucleoside transporters), member 4 | 4,75E-05 | -1,450 |
| Hhatl | hedgehog acyltransferase-like | 4,24E-04 | -1,456 |
| Slc30a1 | solute carrier family 30 (zinc transporter), member 1 | 5,35E-04 | -1,464 |
| Tmem106a | transmembrane protein 106A | 6,52E-05 | -1,485 |
| Mt1 | metallothionein 1 | 1,70E-04 | -1,689 |
| Aqp7 | aquaporin 7 | 1,12E-04 | -1,775 |
| **Hormones/growth factors/receptors/neuropeptides and exocytosis** | | | |
| Mc5r | melanocortin 5 receptor | 1,49E-03 | 2,629 |
| Gabra4 | gamma-aminobutyric acid (GABA) A receptor, subunit alpha | 1,52E-04 | 2,479 |
| Tnfrsf23 | tumor necrosis factor receptor superfamily, member 23 | 3,52E-04 | 1,937 |
| Sycn | syncollin | 1,32E-02 | 1,781 |
| Inhba | inhibin beta-A | 6,49E-06 | 1,709 |
| Tgfb3 | transforming growth factor, beta 3 | 2,55E-05 | 1,553 |
| Egf | epidermal growth factor | 7,13E-06 | 1,533 |
| Nucb2 | nucleobindin 2 | 6,08E-06 | 1,525 |
| Rab3d | RAB3D, member RAS oncogene family | 2,47E-06 | 1,469 |
| Oxtr | oxytocin receptor | 8,43E-03 | 1,435 |
| Ldlrad3 | low density lipoprotein receptor class A domain containing 3 | 5,01E-03 | 1,382 |
| Stc1 | stanniocalcin 1 | 4,62E-03 | 1,381 |
| Gabbr2 | gamma-aminobutyric acid (GABA) B receptor, 2 | 2,68E-06 | 1,340 |
| Ffar2 | free fatty acid receptor 2 | 1,31E-02 | 1,333 |
| Cadps2 | Ca2+-dependent activator protein for secretion 2 | 6,52E-05 | 1,319 |
| Olfr558 | olfactory receptor 558 | 1,29E-02 | 1,319 |
| Sidt2 | SID1 transmembrane family, member 2 | 7,46E-06 | 1,318 |
| Ednrb | endothelin receptor type B | 6,12E-03 | 1,316 |
| Vgf | VGF nerve growth factor inducible | 6,67E-04 | 1,296 |
| Gprc5b | G protein-coupled receptor, family C, group 5, member B | 9,43E-03 | 1,293 |
| Olfr115 | olfactory receptor 115 | 4,33E-03 | 1,276 |
| Gabrq | gamma-aminobutyric acid (GABA) A receptor, subunit theta | 3,36E-03 | 1,269 |
| Gpr180 | G protein-coupled receptor 180 | 7,45E-06 | 1,265 |
| Olfr1015 | olfactory receptor 1015 | 3,85E-02 | 1,255 |
| Olfr857 | olfactory receptor 857 | 2,11E-02 | 1,245 |
| Ror1 | receptor tyrosine kinase-like orphan receptor 1 | 3,61E-02 | 1,236 |
| Olfr203 | olfactory receptor 203 | 1,29E-02 | 1,236 |
| Ros1 | Ros1 proto-oncogene | 1,51E-02 | 1,234 |
| Olfr804 | olfactory receptor 804 | 5,44E-04 | 1,234 |
| Ednra | endothelin receptor type A | 4,94E-02 | 1,233 |
| Olfr1295 | olfactory receptor 1295 | 5,94E-03 | 1,229 |
| Olfr174 | olfactory receptor 174 | 4,64E-04 | 1,219 |
| Olfr361 | olfactory receptor 361 | 1,12E-03 | 1,219 |
| Lin7a | lin-7 homolog A (C. elegans) | 2,61E-02 | 1,215 |
| Pdgfrb | platelet derived growth factor receptor, beta polypeptide | 1,63E-02 | 1,212 |
| Vegfc | vascular endothelial growth factor C | 8,40E-04 | 1,210 |
| Shank1 | SH3/ankyrin domain gene 1 | 1,64E-04 | -1,209 |
| Trpm2 | transient receptor potential cation channel, subfamily M, member 2 | 2,23E-02 | -1,210 |
| Fgfr4 | fibroblast growth factor receptor 4 | 1,88E-03 | -1,210 |
| Rara | retinoic acid receptor, alpha | 7,30E-03 | -1,213 |
| Stxbp3a | syntaxin binding protein 3A | 3,48E-03 | -1,219 |
| Maob | monoamine oxidase B | 1,07E-03 | -1,226 |
| Ephb2 | Eph receptor B2 | 2,38E-03 | -1,226 |
| Olfr814 | olfactory receptor 814 | 6,51E-04 | -1,237 |
| Avpr1b | arginine vasopressin receptor 1B | 6,32E-03 | -1,239 |
| Tmem123 | transmembrane protein 123 | 6,08E-06 | -1,245 |
| Fgf14 | fibroblast growth factor 14 | 2,60E-03 | -1,250 |
| Tfrc | transferrin receptor | 1,00E-02 | -1,250 |
| Sorcs2 | sortilin-related VPS10 domain containing receptor 2 | 4,10E-03 | -1,251 |
| Hgf | hepatocyte growth factor | 1,74E-02 | -1,252 |
| Grik5 | glutamate receptor, ionotropic, kainate 5 (gamma 2) | 1,76E-04 | -1,256 |
| Grin2c | glutamate receptor, ionotropic, NMDA2C (epsilon 3) | 3,47E-02 | -1,258 |
| Cacna1b | calcium channel, voltage-dependent, N type, alpha 1B | 9,19E-03 | -1,271 |
| S1pr2 | sphingosine-1-phosphate receptor 2 | 6,68E-03 | -1,276 |
| Cd79a | CD79A antigen (immunoglobulin-associated alpha) | 4,68E-03 | -1,285 |
| Rnf213 | ring finger protein 213 | 1,69E-02 | -1,290 |
| Sorcs1 | VPS10 domain receptor protein SORCS 1 | 9,54E-04 | -1,304 |
| Mrap2 | melanocortin 2 receptor accessory protein 2 | 1,25E-02 | -1,326 |
| Gpr137b | G protein-coupled receptor 137B | 1,31E-03 | -1,361 |
| Rab3c | RAB3C, member RAS oncogene family | 9,19E-05 | -1,372 |
| Gria2 | glutamate receptor, ionotropic, AMPA2 (alpha 2) | 5,35E-03 | -1,375 |
| Tac1 | tachykinin 1 | 4,59E-02 | -1,376 |
| Olfm2 | olfactomedin 2 | 6,08E-06 | -1,417 |
| Gipr | gastric inhibitory polypeptide receptor | 4,04E-03 | -1,437 |
| Gria3 | glutamate receptor, ionotropic, AMPA3 (alpha 3) | 8,85E-04 | -1,459 |
| Ghrl | ghrelin | 2,85E-02 | -1,469 |
| Sult1c2 | sulfotransferase family, cytosolic, 1C, member 2 | 6,65E-03 | -1,477 |
| Cntfr | ciliary neurotrophic factor receptor | 2,41E-04 | -1,541 |
| Glra1 | glycine receptor, alpha 1 subunit | 2,71E-03 | -1,557 |
| Itpr1 | inositol 1,4,5-triphosphate receptor 1 | 9,01E-04 | -1,621 |
| Agt | angiotensinogen (serpin peptidase inhibitor, clade A, member 8) | 1,01E-03 | -1,661 |
| Chrna4 | cholinergic receptor, nicotinic, alpha polypeptide 4 | 1,06E-05 | -1,766 |
| Olfr1322 | olfactory receptor 1322 | 2,51E-02 | -2,026 |
| Stxbp5l | syntaxin binding protein 5-like | 8,28E-06 | -2,098 |
| **Signal transduction** | | | |
| C1qtnf1 | C1q and tumor necrosis factor related protein 1 | 2,16E-03 | 1,751 |
| Cthrc1 | collagen triple helix repeat containing 1 | 9,39E-05 | 1,746 |
| Apcdd1 | adenomatosis polyposis coli down-regulated 1 | 2,42E-04 | 1,485 |
| Shcbp1 | Shc SH2-domain binding protein 1 | 6,54E-03 | 1,422 |
| Tspan6 | tetraspanin 6 | 8,04E-04 | 1,409 |
| Clps | colipase, pancreatic | 3,23E-02 | 1,409 |
| Cdk18 | cyclin-dependent kinase 18 | 1,54E-04 | 1,367 |
| Mctp1 | multiple C2 domains, transmembrane 1 | 2,79E-02 | 1,352 |
| Cyb561 | cytochrome b-561 | 2,05E-04 | 1,350 |
| Ell2 | elongation factor RNA polymerase II 2 | 1,02E-05 | 1,290 |
| Steap4 | STEAP family member 4 | 8,24E-03 | 1,279 |
| Taar1 | trace amine-associated receptor 1 | 4,52E-04 | 1,273 |
| Tspan15 | tetraspanin 15 | 5,58E-03 | 1,257 |
| Taar4 | trace amine-associated receptor 4 | 2,30E-02 | 1,256 |
| Rgs5 | regulator of G-protein signaling 5 | 1,79E-03 | 1,254 |
| Gpr135 | G protein-coupled receptor 135 | 2,50E-02 | 1,230 |
| Gna14 | guanine nucleotide binding protein, alpha 14 | 2,51E-03 | 1,228 |
| Igfbp3 | insulin-like growth factor binding protein 3 | 1,33E-02 | 1,228 |
| Dusp6 | dual specificity phosphatase 6 | 4,91E-02 | 1,218 |
| Pcp4 | Purkinje cell protein 4 | 4,54E-03 | 1,212 |
| Rasa3 | RAS p21 protein activator 3 | 3,39E-02 | 1,209 |
| Crb1 | crumbs homolog 1 (Drosophila) | 1,85E-03 | 1,208 |
| Ms4a7 | membrane-spanning 4-domains, subfamily A, member 7 | 1,95E-02 | 1,203 |
| Tbl3 | transducin (beta)-like 3 | 2,20E-02 | 1,200 |
| Axin2 | axin2 | 2,93E-02 | -1,200 |
| Dos | downstream of Stk11 | 2,89E-03 | -1,203 |
| Strn | striatin, calmodulin binding protein | 1,55E-02 | -1,205 |
| Cdon | cell adhesion molecule-related/down-regulated by oncogenes | 5,13E-03 | -1,205 |
| Bid | BH3 interacting domain death agonist | 1,05E-02 | -1,207 |
| Il6st | interleukin 6 signal transducer | 2,79E-03 | -1,207 |
| Gabbr1 | gamma-aminobutyric acid (GABA) B receptor, 1 | 1,04E-02 | -1,208 |
| Cntnap1 | contactin associated protein-like 1 | 9,37E-05 | -1,209 |
| Notch1 | Notch gene homolog 1 (Drosophila) | 1,92E-03 | -1,211 |
| Dnaja1 | DnaJ (Hsp40) homolog, subfamily A, member 1 | 2,05E-02 | -1,220 |
| Arhgef9 | CDC42 guanine nucleotide exchange factor (GEF) 9 | 2,22E-02 | -1,223 |
| Dusp10 | dual specificity phosphatase 10 | 4,76E-03 | -1,227 |
| Adrbk2 | adrenergic receptor kinase, beta 2 | 8,72E-03 | -1,233 |
| Inpp5e | inositol polyphosphate-5-phosphatase E | 7,10E-03 | -1,236 |
| Sema3e | sema domain, immunoglobulin domain (Ig), short basic Domain, Secreted,(Semaphorin) 3E | 6,78E-03 | -1,239 |
| Gpr21 | G protein-coupled receptor 21 | 3,58E-02 | -1,242 |
| Magi2 | membrane associated guanylate kinase, WW and PDZ domain | 2,00E-02 | -1,249 |
| Gpr179 | G protein-coupled receptor 179 | 2,31E-03 | -1,255 |
| Fam126a | family with sequence similarity 126, member A | 3,00E-04 | -1,265 |
| Odz2 | odd Oz/ten-m homolog 2 (Drosophila) | 4,08E-03 | -1,284 |
| Lrp1 | low density lipoprotein receptor-related protein 1 | 2,85E-02 | -1,293 |
| Dact1 | dapper homolog 1, antagonist of beta-catenin (xenopus) | 2,10E-02 | -1,306 |
| Crhr1 | corticotropin releasing hormone receptor 1 | 9,42E-03 | -1,307 |
| Pik3c2g | phosphatidylinositol 3-kinase, C2 domain containing, gamma | 2,96E-02 | -1,308 |
| Arhgap5 | Rho GTPase activating protein 5 | 1,25E-03 | -1,311 |
| Lpp | LIM domain containing preferred translocation partner in lipoma | 1,07E-03 | -1,314 |
| Mapk15 | mitogen-activated protein kinase 15 | 3,37E-03 | -1,321 |
| Gprc5c | G protein-coupled receptor, family C, group 5, member C | 7,00E-03 | -1,335 |
| Baiap3 | BAI1-associated protein 3 | 7,78E-03 | -1,358 |
| Gpr98 | G protein-coupled receptor 98 | 3,09E-03 | -1,373 |
| Psd4 | pleckstrin and Sec7 domain containing 4 | 3,37E-03 | -1,417 |
| Gpr75 | G protein-coupled receptor 75 | 1,01E-03 | -1,422 |
| Nrcam | neuron-glia-CAM-related cell adhesion molecule | 1,36E-02 | -1,425 |
| Sfrp5 | secreted frizzled-related sequence protein 5 | 9,32E-04 | -1,455 |
| Dpp10 | dipeptidylpeptidase 10 | 7,62E-04 | -1,629 |
| Npy | neuropeptide Y | 6,97E-05 | -2,110 |
| **AMPK and mTOR pathways** | | | |
| Ppargc1a | peroxisome proliferative activated receptor, gamma, coactivator 1 alpha | 1,09E-03 | 1,612 |
| Akt1s1 | AKT1 substrate 1 (proline-rich) | 2,17E-04 | 1,316 |
| Akt3 | thymoma viral proto-oncogene 3 | 4,09E-04 | -1,208 |
| Prkab2 | protein kinase, AMP-activated, beta 2 non-catalytic subunit | 4,66E-03 | -1,226 |
| Rps6ka5 | ribosomal protein S6 kinase, polypeptide 5 | 2,93E-02 | -1,237 |
| Rictor | RPTOR independent companion of MTOR, complex 2 | 1,09E-03 | -1,266 |
| Pten | phosphatase and tensin homolog | 3,81E-02 | -1,411 |
| Nptx1 | neuronal pentraxin 1 | 1,38E-03 | -1,430 |
| **Insulin signaling pathway** | | | |
| Shc4 | SHC (Src homology 2 domain containing) family, member 4 | 2,68E-04 | 1,514 |
| Dok5 | docking protein 5 | 1,91E-03 | 1,306 |
| Doc2b | double C2, beta | 1,40E-02 | -1,245 |
| Grb10 | growth factor receptor bound protein 10 | 6,83E-03 | -1,250 |
| Shc2 | SHC (Src homology 2 domain containing) transforming prot | 1,90E-03 | -1,284 |
| Srsf3 | arginine-rich splicing factor 3 | 1,07E-03 | -1,421 |
| Nnat | neuronatin | 9,36E-04 | -1,530 |
| Igfbp5 | insulin-like growth factor binding protein 5 | 2,08E-04 | -1,608 |
| **GTPase activity and regulation** | | | |
| Depdc1a | DEP domain containing 1a | 1,38E-03 | 1,874 |
| Arhgap11a | Rho GTPase activating protein 11A | 1,09E-03 | 1,831 |
| Arhgdig | Rho GDP dissociation inhibitor (GDI) gamma | 5,34E-05 | 1,622 |
| Rasd2 | RASD family, member 2 | 2,59E-03 | 1,477 |
| Rasgrf2 | RAS protein-specific guanine nucleotide-releasing factor | 3,00E-04 | 1,472 |
| Arhgef19 | Rho guanine nucleotide exchange factor (GEF) 19 | 5,44E-03 | 1,468 |
| Ralgapa2 | Ral GTPase activating protein, alpha subunit 2 (catalytic) | 3,73E-06 | 1,447 |
| Arhgap19 | Rho GTPase activating protein 19 | 1,27E-02 | 1,439 |
| Rapgef5 | Rap guanine nucleotide exchange factor (GEF) 5 | 3,60E-03 | 1,377 |
| Iqgap3 | IQ motif containing GTPase activating protein 3 | 3,27E-02 | 1,343 |
| Dennd2d | DENN/MADD domain containing 2D | 7,97E-05 | 1,285 |
| Arhgef37 | Rho guanine nucleotide exchange factor (GEF) 37 | 3,74E-03 | 1,269 |
| Limk1 | LIM-domain containing, protein kinase | 1,63E-03 | 1,264 |
| Vav3 | vav 3 oncogene | 4,89E-04 | 1,239 |
| Arhgap18 | Rho GTPase activating protein 18 | 7,02E-04 | 1,227 |
| Rhoc | ras homolog gene family, member C | 6,76E-03 | 1,211 |
| Gng12 | guanine nucleotide binding protein (G protein), gamma 1 | 1,22E-02 | 1,202 |
| Rasgrf1 | RAS protein-specific guanine nucleotide-releasing factor | 4,91E-02 | -1,200 |
| Rreb1 | ras responsive element binding protein 1 | 8,01E-04 | -1,200 |
| Dab2ip | disabled homolog 2 (Drosophila) interacting protein | 1,82E-04 | -1,209 |
| Dennd4c | DENN/MADD domain containing 4C | 7,51E-03 | -1,217 |
| Gmip | Gem-interacting protein | 2,05E-04 | -1,219 |
| Radil | Ras association and DIL domains | 2,81E-03 | -1,220 |
| Srgap3 | SLIT-ROBO Rho GTPase activating protein 3 | 9,03E-04 | -1,223 |
| Sept3 | septin 3 | 4,05E-03 | -1,225 |
| Tbc1d2b | TBC1 domain family, member 2B | 2,27E-03 | -1,225 |
| Rem2 | rad and gem related GTP binding protein 2 | 3,56E-02 | -1,239 |
| Rragb | Ras-related GTP binding B | 7,44E-04 | -1,241 |
| Spata13 | spermatogenesis associated 13 | 2,83E-02 | -1,242 |
| Agap2 | ArfGAP with GTPase domain, ankyrin repeat and PH domain | 1,72E-02 | -1,245 |
| Psd3 | pleckstrin and Sec7 domain containing 3 | 1,16E-02 | -1,253 |
| Rasgef1a | RasGEF domain family, member 1A | 5,61E-03 | -1,289 |
| Rab12 | RAB12, member RAS oncogene family | 7,66E-04 | -1,321 |
| Dock6 | dedicator of cytokinesis 6 | 2,44E-03 | -1,329 |
| Rit2 | Ras-like without CAAX 2 | 6,25E-03 | -1,368 |
| Srgap1 | SLIT-ROBO Rho GTPase activating protein 1 | 2,03E-03 | -1,405 |
| Mlph | melanophilin | 1,10E-03 | -1,488 |
| **Kinases/phosphatases and related proteins** | | | |
| Pbk | PDZ binding kinase | 8,40E-04 | 2,426 |
| Akap6 | A kinase (PRKA) anchor protein 6 | 2,02E-06 | 1,993 |
| Dusp23 | dual specificity phosphatase 23 | 6,67E-05 | 1,436 |
| Ckmt1 | creatine kinase, mitochondrial 1, ubiquitous | 8,00E-06 | 1,373 |
| Mapkapk3 | mitogen-activated protein kinase-activated protein kinase | 7,22E-04 | 1,347 |
| Pi4k2b | phosphatidylinositol 4-kinase type 2 beta | 4,36E-04 | 1,287 |
| Dak | dihydroxyacetone kinase 2 homolog (yeast) | 5,69E-05 | 1,284 |
| Ppapdc3 | phosphatidic acid phosphatase type 2 domain containing 3 | 2,38E-03 | 1,260 |
| Ak3 | adenylate kinase 3 | 8,28E-06 | 1,259 |
| Stk32b | serine/threonine kinase 32B | 2,31E-02 | 1,246 |
| Met | met proto-oncogene | 8,36E-03 | 1,243 |
| Mtmr11 | myotubularin related protein 11 | 1,01E-03 | 1,238 |
| Ppp2r3c | protein phosphatase 2, regulatory subunit B'', gamma | 8,29E-04 | 1,227 |
| Lrrc67 | leucine rich repeat containing 67 | 1,61E-02 | 1,223 |
| Tek | endothelial-specific receptor tyrosine kinase | 2,27E-02 | 1,216 |
| Ibtk | inhibitor of Bruton agammaglobulinemia tyrosine kinase | 2,05E-03 | 1,209 |
| Camk1d | calcium/calmodulin-dependent protein kinase ID | 3,06E-02 | 1,208 |
| Prkce | protein kinase C, epsilon | 5,35E-04 | -1,202 |
| Agphd1 | aminoglycoside phosphotransferase domain containing 1 | 2,08E-02 | -1,203 |
| Pank1 | pantothenate kinase 1 | 1,54E-02 | -1,209 |
| Ulk4 | unc-51-like kinase 4 (C. elegans) | 1,55E-03 | -1,218 |
| Inpp4a | inositol polyphosphate-4-phosphatase, type I | 5,94E-03 | -1,235 |
| Phlpp2 | PH domain and leucine rich repeat protein phosphatase | 2,17E-04 | -1,238 |
| Dusp18 | dual specificity phosphatase 18 | 2,86E-03 | -1,240 |
| Camk4 | calcium/calmodulin-dependent protein kinase IV | 2,80E-03 | -1,241 |
| Rad54l2 | RAD54 like 2 (S. cerevisiae) | 5,26E-03 | -1,244 |
| Cdkl1 | cyclin-dependent kinase-like 1 (CDC2-related kinase) | 5,64E-03 | -1,257 |
| Itpkb | inositol 1,4,5-trisphosphate 3-kinase B | 3,67E-04 | -1,269 |
| Ccnl1 | cyclin L1 | 1,40E-04 | -1,275 |
| Dmpk | dystrophia myotonica-protein kinase | 9,84E-04 | -1,277 |
| Dbn1 | drebrin 1 | 7,62E-04 | -1,286 |
| Ppp2r2b | protein phosphatase 2 (formerly 2A), regulatory subunit | 1,21E-04 | -1,288 |
| Ptprd | protein tyrosine phosphatase, receptor type, D | 2,83E-02 | -1,291 |
| Wnk1 | WNK lysine deficient protein kinase 1 | 2,82E-02 | -1,421 |
| Nudt11 | nudix (nucleoside diphosphate linked moiety X)-type motif 11 | 4,30E-03 | -1,423 |
| Ncs1 | neuronal calcium sensor 1 | 4,23E-04 | -1,465 |
| Upp1 | uridine phosphorylase 1 | 4,36E-05 | -1,517 |
| Mast1 | microtubule associated serine/threonine kinase 1 | 2,65E-04 | -1,587 |
| **Cell-cell signaling** | | | |
| Hmmr | hyaluronan mediated motility receptor (RHAMM) | 2,84E-04 | 2,501 |
| Vipr2 | vasoactive intestinal peptide receptor 2 | 2,04E-03 | 1,215 |
| Dll1 | delta-like 1 (Drosophila) | 6,45E-04 | -1,343 |
| **Extracellular matrix/collagen formation** | | | |
| Leprel1 | leprecan-like 1 | 2,22E-04 | 2,021 |
| F13a1 | coagulation factor XIII, A1 subunit | 2,88E-06 | 1,861 |
| Sgcd | sarcoglycan, delta (dystrophin-associated glycoprotein) | 1,12E-04 | 1,608 |
| Smoc1 | SPARC related modular calcium binding 1 | 1,57E-04 | 1,566 |
| Hapln4 | hyaluronan and proteoglycan link protein 4 | 4,48E-05 | 1,519 |
| Spon2 | spondin 2, extracellular matrix protein | 1,41E-03 | 1,497 |
| Frem2 | Fras1 related extracellular matrix protein 2 | 2,89E-05 | 1,493 |
| Lepre1 | leprecan 1 | 8,77E-04 | 1,339 |
| Naglu | alpha-N-acetylglucosaminidase (Sanfilippo disease IIIB) | 2,27E-04 | 1,327 |
| Lama5 | laminin, alpha 5 | 4,06E-03 | 1,269 |
| Plod3 | procollagen-lysine, 2-oxoglutarate 5-dioxygenase 3 | 2,55E-04 | 1,244 |
| P4ha2 | Prolyl 4-Hydroxylase, Alpha Polypeptide II | 1,69E-02 | 1,210 |
| Col6a6 | collagen, type VI, alpha 6 | 3,57E-02 | -1,232 |
| Ltbp1 | latent transforming growth factor beta binding protein 1 | 1,99E-03 | -1,271 |
| Mfap1a | microfibrillar-associated protein 1A | 1,35E-04 | -1,290 |
| Egflam | EGF-like, fibronectin type III and laminin G domains | 5,58E-04 | -1,472 |
| **Chemokines/cytokines/adhesion molecules/innate immunity and related proteins** | | | |
| Cd44 | CD44 antigen | 8,28E-06 | 1,729 |
| Susd2 | sushi domain containing 2 | 8,77E-05 | 1,638 |
| Tubb6 | tubulin, beta 6 | 8,08E-03 | 1,547 |
| Emilin1 | elastin microfibril interfacer 1 | 8,13E-05 | 1,533 |
| Troap | trophinin associated protein | 8,25E-04 | 1,495 |
| Thbs1 | thrombospondin 1 | 1,44E-02 | 1,466 |
| Il1r2 | interleukin 1 receptor, type II | 3,81E-03 | 1,450 |
| Cldn1 | claudin 1 | 2,72E-04 | 1,421 |
| Pcdh18 | protocadherin 18 | 4,19E-03 | 1,419 |
| Nid2 | nidogen 2 | 8,99E-05 | 1,407 |
| Otoa | otoancorin | 1,64E-03 | 1,390 |
| Clec14a | C-type lectin domain family 14, member a | 6,48E-04 | 1,382 |
| Vstm2a | V-set and transmembrane domain containing 2A | 5,39E-04 | 1,365 |
| Fam19a1 | family with sequence similarity 19, member A1 | 4,25E-03 | 1,335 |
| Cd34 | CD34 antigen | 1,68E-03 | 1,335 |
| Igsf5 | immunoglobulin superfamily, member 5 | 1,05E-03 | 1,335 |
| Gja1 | gap junction protein, alpha 1 | 2,77E-02 | 1,329 |
| Jam2 | junction adhesion molecule 2 | 1,81E-04 | 1,325 |
| Muc4 | mucin 4 | 4,89E-04 | 1,324 |
| Cldn3 | claudin 3 | 9,94E-04 | 1,290 |
| Eng | endoglin | 7,52E-04 | 1,280 |
| Gucy1b3 | guanylate cyclase 1, soluble, beta 3 | 2,12E-03 | 1,275 |
| Mif | macrophage migration inhibitory factor | 4,68E-03 | 1,272 |
| Lama4 | laminin, alpha 4 | 1,11E-02 | 1,266 |
| Amigo3 | adhesion molecule with Ig like domain 3 | 6,81E-03 | 1,263 |
| Lgals9 | lectin, galactose binding, soluble 9 | 2,96E-02 | 1,259 |
| Rpsa | ribosomal protein SA | 3,94E-05 | 1,257 |
| Cd200 | CD200 antigen | 3,02E-04 | 1,251 |
| Gp5 | glycoprotein 5 (platelet) | 3,37E-03 | 1,249 |
| Clec1a | C-type lectin domain family 1, member a | 4,00E-02 | 1,246 |
| Lpar1 | lysophosphatidic acid receptor 1 | 3,03E-03 | 1,245 |
| Dsc2 | desmocollin 2 | 3,97E-03 | 1,233 |
| Crlf1 | cytokine receptor-like factor 1 | 4,03E-03 | 1,226 |
| Crisp2 | cysteine-rich secretory protein 2 | 6,64E-03 | 1,222 |
| Dock4 | dedicator of cytokinesis 4 | 2,77E-02 | 1,218 |
| Crlf2 | cytokine receptor-like factor 2 | 5,30E-03 | 1,217 |
| Actr1b | ARP1 actin-related protein 1 homolog B, centractin beta (Yeast) | 8,12E-04 | 1,210 |
| Igsf11 | immunoglobulin superfamily, member 11 | 8,04E-03 | 1,206 |
| Ccrl2 | chemokine (C-C motif) receptor-like 2 | 1,85E-02 | 1,205 |
| Tjp2 | tight junction protein 2 | 1,57E-03 | -1,204 |
| Zfr2 | zinc finger RNA binding protein 2 | 2,12E-03 | -1,209 |
| Sned1 | sushi, nidogen and EGF-like domains 1 | 4,30E-04 | -1,215 |
| Iglon5 | IgLON family member 5 | 1,09E-02 | -1,215 |
| Pcdh9 | protocadherin 9 | 3,51E-02 | -1,218 |
| Cd24a | CD24a antigen | 2,88E-02 | -1,219 |
| Igsf9b | immunoglobulin superfamily, member 9B | 4,31E-03 | -1,232 |
| Il18 | interleukin 18 | 1,10E-02 | -1,234 |
| Cxcl13 | chemokine (C-X-C motif) ligand 13 | 2,81E-02 | -1,235 |
| Ier2 | immediate early response 2 | 6,72E-03 | -1,239 |
| Cldn11 | claudin 11 | 1,21E-03 | -1,247 |
| Pkhd1 | polycystic kidney and hepatic disease 1 | 1,38E-02 | -1,263 |
| Csf1 | colony stimulating factor 1 (macrophage) | 2,68E-03 | -1,273 |
| Cntn3 | contactin 3 | 2,08E-04 | -1,290 |
| Ppfia2 | protein tyrosine phosphatase, receptor type, f polypeptide (PTPRF), interacting protein (liprin), alpha 2 | 5,12E-03 | -1,293 |
| Dscam | Down syndrome cell adhesion molecule | 5,09E-03 | -1,298 |
| Tnr | tenascin R | 1,43E-02 | -1,300 |
| Ppl | periplakin | 2,97E-02 | -1,313 |
| Cer1 | cerberus 1 homolog (Xenopus laevis) | 2,77E-03 | -1,324 |
| Hmgb1 | high mobility group box 1 | 1,80E-02 | -1,340 |
| Ntm | neurotrimin | 8,79E-03 | -1,346 |
| C8b | complement component 8, beta polypeptide | 2,41E-04 | -1,363 |
| Syt1 | synaptotagmin I | 1,24E-02 | -1,369 |
| Ncam2 | neural cell adhesion molecule 2 | 1,41E-03 | -1,388 |
| L1cam | L1 cell adhesion molecule | 2,12E-03 | -1,389 |
| Cdh22 | cadherin 22 | 6,71E-04 | -1,397 |
| Cdh7 | cadherin 7, type 2 | 1,06E-03 | -1,482 |
| Dnm1 | dynamin 1 | 9,79E-06 | -1,577 |
| Igsf21 | immunoglobin superfamily, member 21 | 8,81E-04 | -1,587 |
| Flrt1 | fibronectin leucine rich transmembrane protein 1 | 1,37E-03 | -1,616 |
| Pcdh15 | protocadherin 15 | 1,39E-04 | -1,682 |
| **HLA-related** | | | |
| H2-T22 | histocompatibility 2, T region locus 22 | 6,14E-05 | 1,314 |
| Tapbpl | TAP binding protein-like | 1,48E-04 | 1,259 |
| H13 | histocompatibility 13 | 2,17E-04 | 1,243 |
| **Other functions** | | | |
| Cuzd1 | CUB and zona pellucida-like domains 1 | 2,06E-02 | 2,735 |
| Pdyn | prodynorphin | 3,63E-04 | 2,585 |
| Sema3c | sema domain, immunoglobulin domain (Ig), short basic domain | 7,05E-05 | 2,474 |
| Necab2 | N-terminal EF-hand calcium binding protein 2 | 6,50E-07 | 2,060 |
| Mfi2 | antigen p97 (melanoma associated) identified by monoclonal antibodies 133.2 and 96.5 | 1,48E-04 | 1,962 |
| S100z | S100 calcium binding protein, zeta | 2,70E-03 | 1,818 |
| Creld2 | cysteine-rich with EGF-like domains 2 | 4,82E-05 | 1,631 |
| Stil | Scl/Tal1 interrupting locus | 1,79E-03 | 1,628 |
| Tmem160 | transmembrane protein 160 | 1,79E-05 | 1,524 |
| Gnat2 | guanine nucleotide binding protein, alpha transducing 2 | 7,69E-04 | 1,515 |
| Nrm | nurim (nuclear envelope membrane protein) | 1,74E-03 | 1,485 |
| Car5b | carbonic anhydrase 5b, mitochondrial | 1,12E-03 | 1,483 |
| Yif1b | Yip1 interacting factor homolog B (S. cerevisiae) | 9,19E-05 | 1,475 |
| Mum1l1 | melanoma associated antigen (mutated) 1-like 1 | 4,49E-02 | 1,440 |
| St7 | suppression of tumorigenicity 7 | 4,71E-04 | 1,381 |
| Csn3 | casein kappa | 4,05E-03 | 1,377 |
| Pon3 | paraoxonase 3 | 2,49E-04 | 1,376 |
| Rassf4 | Ras association (RalGDS/AF-6) domain family member 4 | 1,51E-04 | 1,371 |
| Tmem150a | transmembrane protein 150A | 1,51E-04 | 1,366 |
| Nomo1 | nodal modulator 1 | 1,75E-05 | 1,358 |
| Fez1 | fasciculation and elongation protein zeta 1 (zygin I) | 2,89E-05 | 1,358 |
| Morc1 | microrchidia 1 | 5,50E-04 | 1,342 |
| Lrrtm2 | leucine rich repeat transmembrane neuronal 2 | 2,68E-03 | 1,334 |
| Tmem66 | transmembrane protein 66 | 7,45E-06 | 1,332 |
| Atp13a1 | ATPase type 13A1 | 1,87E-04 | 1,309 |
| Gmfg | glia maturation factor, gamma | 9,64E-03 | 1,297 |
| Prom1 | prominin 1 | 1,64E-02 | 1,290 |
| Zdhhc24 | zinc finger, DHHC domain containing 24 | 8,88E-05 | 1,284 |
| Arfip2 | ADP-ribosylation factor interacting protein 2 | 5,95E-04 | 1,276 |
| Dcx | doublecortin | 2,06E-02 | 1,270 |
| Pxmp4 | peroxisomal membrane protein 4 | 6,39E-05 | 1,270 |
| Sprr1a | small proline-rich protein 1A | 4,73E-02 | 1,242 |
| Tal2 | T-cell acute lymphocytic leukemia 2 | 2,56E-03 | 1,241 |
| Cc2d2a | coiled-coil and C2 domain containing 2A | 5,93E-05 | 1,239 |
| Sez6l2 | seizure related 6 homolog like 2 | 1,73E-04 | 1,238 |
| Shisa2 | shisa homolog 2 (Xenopus laevis) | 7,27E-03 | 1,223 |
| Chid1 | chitinase domain containing 1 | 6,03E-04 | 1,220 |
| Pex11c | peroxisomal biogenesis factor 11 gamma | 2,97E-03 | 1,215 |
| Wdr62 | WD repeat domain 62 | 9,26E-03 | 1,213 |
| Ogfod2 | 2-oxoglutarate and iron-dependent oxygenase domain containing 2 | 5,71E-04 | 1,212 |
| Mgp | matrix Gla protein | 2,86E-02 | 1,209 |
| Timm50 | translocase of inner mitochondrial membrane 50 homolog (yeast) | 7,59E-04 | 1,206 |
| Pdzd11 | PDZ domain containing 11 | 1,19E-02 | 1,205 |
| Tmem43 | transmembrane protein 43 | 2,24E-04 | 1,204 |
| Fam114a1 | family with sequence similarity 114, member A1 | 2,81E-04 | 1,204 |
| Dpysl5 | dihydropyrimidinase-like 5 | 9,50E-03 | -1,208 |
| Evpl | envoplakin | 3,15E-04 | -1,210 |
| Zcchc11 | zinc finger, CCHC domain containing 11 | 6,66E-04 | -1,215 |
| Sp140 | Sp140 nuclear body protein | 2,62E-02 | -1,217 |
| D5Ertd579e | DNA segment, Chr 5, ERATO Doi 579, expressed | 2,72E-02 | -1,220 |
| Olfml3 | olfactomedin-like 3 | 3,16E-02 | -1,230 |
| Hpca | hippocalcin | 4,84E-04 | -1,233 |
| Tnrc6c | trinucleotide repeat containing 6C | 2,32E-04 | -1,234 |
| Plp1 | proteolipid protein (myelin) 1 | 3,63E-02 | -1,234 |
| Fam113b | family with sequence similarity 113, member B | 5,79E-03 | -1,234 |
| Sobp | sine oculis-binding protein homolog (Drosophila) | 5,93E-05 | -1,236 |
| Sftpa1 | surfactant associated protein A1 | 3,65E-02 | -1,242 |
| Zswim6 | zinc finger, SWIM domain containing 6 | 4,13E-03 | -1,242 |
| Fcho1 | FCH domain only 1 | 6,35E-05 | -1,250 |
| Cryba2 | crystallin, beta A2 | 1,64E-02 | -1,252 |
| Iqcb1 | IQ calmodulin-binding motif containing 1 | 1,39E-04 | -1,256 |
| Phldb2 | pleckstrin homology-like domain, family B, member 2 | 4,88E-03 | -1,258 |
| Acrbp | proacrosin binding protein | 1,16E-02 | -1,260 |
| Nsg1 | neuron specific gene family member 1 | 5,33E-04 | -1,268 |
| Mrfap1 | Morf4 family associated protein 1 | 6,60E-03 | -1,280 |
| Dlgap1 | discs, large (Drosophila) homolog-associated protein 1 | 4,75E-03 | -1,285 |
| Mreg | melanoregulin | 1,59E-02 | -1,292 |
| Tcp11 | t-complex protein 11 | 7,92E-03 | -1,301 |
| Edaradd | EDAR (ectodysplasin-A receptor)-associated death domain | 2,60E-03 | -1,306 |
| Dab1 | disabled homolog 1 (Drosophila) | 2,54E-04 | -1,314 |
| Gap43 | growth associated protein 43 | 1,62E-03 | -1,325 |
| AW551984 | expressed sequence AW551984 | 1,49E-03 | -1,329 |
| Olfm3 | olfactomedin 3 | 6,87E-03 | -1,330 |
| Dzip1 | DAZ interacting protein 1 | 1,06E-03 | -1,335 |
| Astn2 | astrotactin 2 | 1,34E-03 | -1,335 |
| Zbtb7c | zinc finger and BTB domain containing 7C | 7,22E-04 | -1,350 |
| Tekt2 | tektin 2 | 2,55E-04 | -1,357 |
| Nav3 | neuron navigator 3 | 9,88E-04 | -1,368 |
| Zfp62 | zinc finger protein 62 | 1,95E-04 | -1,374 |
| Car15 | carbonic anhydrase 15 | 8,16E-04 | -1,403 |
| F3 | coagulation factor III | 7,45E-06 | -1,505 |
| Edil3 | EGF-like repeats and discoidin I-like domains 3 | 1,00E-03 | -1,524 |
| Mfap2 | microfibrillar-associated protein 2 | 1,04E-04 | -1,561 |
| Gfra3 | glial cell line derived neurotrophic factor family recepto | 2,07E-03 | -1,690 |
| Mt2 | metallothionein 2 | 3,85E-05 | -1,877 |
| **Unknown functions** | | | |
| Try5 | trypsin 5 | 8,45E-03 | 2,093 |
| Tmem179 | transmembrane protein 179 | 2,75E-04 | 1,876 |
| Svopl | SV2 related protein homolog (rat)-like | 6,85E-05 | 1,865 |
| Myo15b | myosin XVB | 1,89E-05 | 1,864 |
| Try10 | trypsin 10 | 2,41E-02 | 1,837 |
| Prr11 | proline rich 11 | 1,79E-03 | 1,800 |
| Gm5409 | predicted pseudogene 5409 | 1,67E-02 | 1,677 |
| Slfn9 | schlafen 9 | 2,23E-03 | 1,676 |
| Tmed6 | transmembrane emp24 protein transport domain containing 6 | 2,84E-03 | 1,645 |
| Sdf2l1 | stromal cell-derived factor 2-like 1 | 6,39E-05 | 1,530 |
| Fam70a | family with sequence similarity 70, member A | 6,27E-05 | 1,518 |
| Ankrd34b | ankyrin repeat domain 34B | 2,21E-02 | 1,496 |
| Gpr165 | G protein-coupled receptor 165 | 2,18E-02 | 1,492 |
| Ccdc85a | coiled-coil domain containing 85A | 2,82E-04 | 1,481 |
| Btnl9 | butyrophilin-like 9 | 1,47E-05 | 1,440 |
| Sel1l3 | sel-1 suppressor of lin-12-like 3 (C. elegans) | 9,23E-05 | 1,428 |
| Ttc13 | tetratricopeptide repeat domain 13 / | 1,45E-05 | 1,412 |
| Wdr90 | WD repeat domain 90 | 5,29E-04 | 1,405 |
| Fam46a | family with sequence similarity 46, member A | 1,26E-03 | 1,388 |
| Rell1 | RELT-like 1 | 1,02E-05 | 1,356 |
| Tmem125 | transmembrane protein 125 | 8,57E-03 | 1,350 |
| Maged2 | melanoma antigen, family D, 2 | 1,15E-03 | 1,348 |
| Spt1 | salivary protein 1 | 2,62E-02 | 1,347 |
| Tmem212 | transmembrane protein 212 | 7,13E-04 | 1,330 |
| Ccdc107 | coiled-coil domain containing 107 | 9,19E-05 | 1,313 |
| Heatr5b | HEAT repeat containing 5B | 3,58E-04 | 1,312 |
| Gm2897 | predicted gene 2897 | 1,68E-03 | 1,304 |
| Fam194a | family with sequence similarity 194, member A | 3,09E-03 | 1,299 |
| Sval2 | seminal vesicle antigen-like 2 | 8,63E-05 | 1,299 |
| Ttc39c | tetratricopeptide repeat domain 39C | 5,95E-04 | 1,298 |
| Gpr108 | G protein-coupled receptor 108 | 2,06E-04 | 1,295 |
| Pter | phosphotriesterase related | 7,97E-03 | 1,295 |
| Gm5465 | predicted gene 5465 | 1,42E-02 | 1,290 |
| Tmem238 | transmembrane protein 238 | 3,43E-04 | 1,290 |
| D17Wsu104e | DNA segment, Chr 17, Wayne State University 104, expression | 5,93E-05 | 1,287 |
| Reep5 | receptor accessory protein 5 | 7,96E-05 | 1,279 |
| Tmem176a | transmembrane protein 176A | 1,05E-03 | 1,278 |
| Trabd | TraB domain containing | 6,55E-05 | 1,276 |
| Cyyr1 | cysteine and tyrosine-rich protein 1 | 3,24E-04 | 1,276 |
| Lrrc39 | leucine rich repeat containing 39 | 2,89E-03 | 1,274 |
| Ddrgk1 | DDRGK domain containing 1 | 1,76E-04 | 1,273 |
| Vmn1r103 | vomeronasal 1 receptor 103 | 6,51E-03 | 1,268 |
| Fam173a | family with sequence similarity 173, member A | 1,19E-03 | 1,268 |
| Mfap3l | microfibrillar-associated protein 3-like | 1,38E-03 | 1,267 |
| Gm3696 | predicted gene 3696 | 6,87E-04 | 1,265 |
| Tmem223 | transmembrane protein 223 | 9,30E-04 | 1,260 |
| Yipf2 | Yip1 domain family, member 2 | 8,49E-04 | 1,260 |
| Tmem117 | transmembrane protein 117 | 4,84E-04 | 1,255 |
| Tc2n | tandem C2 domains, nuclear | 1,80E-02 | 1,253 |
| Tmem218 | transmembrane protein 218 | 9,06E-04 | 1,252 |
| Ccdc18 | coiled-coil domain containing 18 | 8,12E-04 | 1,251 |
| Vmn1r4 | vomeronasal 1 receptor 4 | 1,10E-03 | 1,249 |
| Ifrd2 | interferon-related developmental regulator 2 | 4,52E-04 | 1,246 |
| Tmem205 | transmembrane protein 205 | 9,03E-04 | 1,245 |
| Gm447 | predicted gene 447 | 1,23E-03 | 1,244 |
| Fbxo48 | F-box protein 48 | 1,26E-02 | 1,243 |
| Tmem214 | transmembrane protein 214 | 2,66E-04 | 1,240 |
| Npdc1 | neural proliferation, differentiation and control gene 1 | 1,45E-04 | 1,231 |
| Nlrp4f | NLR family, pyrin domain containing 4F | 9,04E-03 | 1,230 |
| R3hcc1 | R3H domain and coiled-coil containing 1 | 1,51E-03 | 1,228 |
| Gm561 | predicted gene 561 | 6,56E-03 | 1,228 |
| Tmem120b | transmembrane protein 120B | 3,60E-03 | 1,224 |
| Tmem144 | transmembrane protein 144 | 3,85E-04 | 1,223 |
| Dnajc28 | DnaJ (Hsp40) homolog, subfamily C, member 28 | 5,37E-03 | 1,220 |
| D10Jhu81e | DNA segment, Chr 10, Johns Hopkins University 81 expression | 2,41E-04 | 1,219 |
| Serf2 | small EDRK-rich factor 2 | 1,74E-03 | 1,217 |
| Gm10406 | predicted gene 10406 | 8,18E-03 | 1,211 |
| Gm5105 | predicted gene 5105 | 1,70E-02 | 1,211 |
| Armcx6 | armadillo repeat containing, X-linked 6 | 2,67E-03 | 1,210 |
| Ccdc134 | coiled-coil domain containing 134 | 3,75E-02 | 1,209 |
| Gm6590 | predicted gene 6590 | 5,36E-03 | 1,207 |
| Smyd5 | SET and MYND domain containing 5 | 9,19E-03 | 1,207 |
| Cxx1a | CAAX box 1 homolog A (human) | 4,74E-04 | 1,204 |
| Gm996 | predicted gene 996 | 3,47E-02 | 1,204 |
| Gm10267 | predicted gene 10267 | 4,15E-03 | 1,203 |
| Ifi27l1 | interferon, alpha-inducible protein 27 like 1 | 5,73E-03 | 1,202 |
| Nol4 | nucleolar protein 4 | 4,27E-03 | -1,201 |
| Zcchc7 | zinc finger, CCHC domain containing 7 | 7,68E-03 | -1,202 |
| Zc3h13 | zinc finger CCCH type containing 13 | 4,57E-03 | -1,203 |
| Phxr1 | per-hexamer repeat gene 1 | 4,75E-03 | -1,204 |
| Gm7241 | predicted pseudogene 7241 | 1,37E-02 | -1,205 |
| Zfp125 | zinc finger protein 125 | 1,49E-02 | -1,206 |
| Fnbp4 | formin binding protein 4 | 1,09E-03 | -1,206 |
| Vwa5b2 | von Willebrand factor A domain containing 5B2 | 1,51E-02 | -1,208 |
| H2-K2 | histocompatibility 2, K region locus 2 | 3,45E-02 | -1,209 |
| Zfp760 | zinc finger protein 760 | 1,14E-02 | -1,210 |
| Gm10033 | predicted gene 10033 | 1,84E-02 | -1,210 |
| Mxra7 | matrix-remodelling associated 7 | 6,13E-03 | -1,212 |
| Zfp619 | zinc finger protein 619 | 3,03E-03 | -1,212 |
| Fam172a | family with sequence similarity 172, member A | 8,18E-04 | -1,214 |
| Emid1 | EMI domain containing 1 | 4,30E-03 | -1,214 |
| Ahdc1 | AT hook, DNA binding motif, containing 1 | 5,71E-04 | -1,216 |
| Gm10786 | predicted gene 10786 | 2,82E-02 | -1,216 |
| Gm10589 | predicted gene 10589 | 1,82E-02 | -1,217 |
| Rnf157 | ring finger protein 157 | 5,42E-03 | -1,218 |
| Zfp871 | zinc finger protein 871 | 1,25E-02 | -1,218 |
| Sfrs18 | serine/arginine-rich splicing factor 18 | 1,70E-02 | -1,220 |
| Bod1l | biorientation of chromosomes in cell division 1-like | 1,41E-02 | -1,220 |
| Wdr47 | WD repeat domain 47 | 4,65E-04 | -1,225 |
| Aard | alanine and arginine rich domain containing protein | 3,43E-02 | -1,230 |
| Fam36a | family with sequence similarity 36, member A | 2,28E-02 | -1,233 |
| Nbeal1 | neurobeachin like 1 | 4,80E-02 | -1,236 |
| Gm10838 | predicted gene 10838 | 9,13E-03 | -1,238 |
| Zfp781 | zinc finger protein 781 | 1,22E-03 | -1,240 |
| AU019823 | expressed sequence AU019823 | 8,28E-06 | -1,240 |
| Klhl32 | kelch-like 32 (Drosophila) | 2,62E-03 | -1,240 |
| Plekha5 | pleckstrin homology domain containing, family A member 5 | 2,72E-03 | -1,241 |
| Fam82b | family with sequence similarity 82, member B | 3,60E-03 | -1,242 |
| Trp53i11 | transformation related protein 53 inducible protein | 6,90E-04 | -1,247 |
| Gm13139 | predicted gene 13139 | 1,92E-02 | -1,250 |
| Zfp758 | zinc finger protein 758 | 2,81E-04 | -1,250 |
| Nynrin | NYN domain and retroviral integrase containing | 1,78E-02 | -1,251 |
| Gm1043 | predicted gene 1043 | 4,13E-04 | -1,251 |
| Fam78b | family with sequence similarity 78, member B | 1,82E-04 | -1,253 |
| Zfp229 | zinc finger protein | 1,85E-02 | -1,253 |
| Ceacam10 | carcinoembryonic antigen-related cell adhesion molecule 10 | 4,26E-02 | -1,255 |
| Phf20l1 | PHD finger protein 20-like 1 | 6,14E-04 | -1,255 |
| Gm1027 | predicted gene 1027 | 8,14E-04 | -1,260 |
| Bend7 | BEN domain containing 7 | 9,38E-04 | -1,261 |
| Gm13251 | predicted gene 13251 | 9,03E-03 | -1,266 |
| Gm6999 | predicted gene 6999 | 1,93E-02 | -1,267 |
| Anp32-ps | acidic (leucine-rich) nuclear phosphoprotein 32 family, pseudogene | 8,30E-03 | -1,269 |
| DXBay18 | DNA segment, Chr X, Baylor 18 | 5,47E-03 | -1,272 |
| Zcchc2 | zinc finger, CCHC domain containing 2 | 1,31E-03 | -1,273 |
| Ankrd33b | ankyrin repeat domain 33B | 2,61E-02 | -1,274 |
| Fhdc1 | FH2 domain containing 1 | 6,08E-06 | -1,280 |
| Zfp568 | zinc finger protein 568 | 9,22E-03 | -1,287 |
| Gm6712 | predicted gene 6712 | 3,77E-03 | -1,288 |
| BC068157 | cDNA sequence BC068157 | 1,38E-03 | -1,290 |
| Gm13235 | predicted gene 13235 | 1,49E-02 | -1,290 |
| Gpr137b-ps | G protein-coupled receptor 137B, pseudogene | 2,74E-03 | -1,290 |
| Ociad2 | OCIA domain containing 2 | 1,16E-02 | -1,292 |
| Csmd2 | CUB and Sushi multiple domains 2 | 1,19E-02 | -1,293 |
| 3110048L19Rik | zinc finger pseudogene | 2,80E-04 | -1,296 |
| Tmem229b | transmembrane protein 229B | 2,17E-04 | -1,300 |
| Zmym5 | zinc finger, MYM-type 5 | 5,82E-04 | -1,313 |
| Gm3365 | predicted gene 3365 | 2,36E-03 | -1,314 |
| Pisd-ps2 | phosphatidylserine decarboxylase, pseudogene 2 | 7,00E-04 | -1,315 |
| Lancl3 | LanC lantibiotic synthetase component C-like 3 (bacterial) | 1,53E-02 | -1,317 |
| Xkr6 | X Kell blood group precursor related family member 6 | 2,74E-04 | -1,318 |
| Wdr78 | WD repeat domain 78 | 1,17E-04 | -1,321 |
| Rbm33 | RNA binding motif protein 33 | 3,45E-02 | -1,327 |
| Gm9958 | predicted gene 9958 | 1,22E-04 | -1,327 |
| Ccdc48 | coiled-coil domain containing 48 | 2,04E-05 | -1,330 |
| Gm5595 | predicted gene 5595 | 5,68E-03 | -1,330 |
| Gm7125 | high mobility group nucleosomal binding domain 2 | 1,61E-03 | -1,333 |
| Zfp872 | zinc finger protein 872 | 3,77E-02 | -1,334 |
| Ctxn2 | cortexin 2 | 4,40E-02 | -1,334 |
| Dbpht2 | DNA binding protein with his-thr domain | 8,30E-04 | -1,335 |
| Ankrd45 | ankyrin repeat domain 45 | 2,24E-04 | -1,344 |
| Fam159b | family with sequence similarity 159, member B | 1,22E-04 | -1,354 |
| Gipc2 | GIPC PDZ domain containing family, member 2 | 1,06E-03 | -1,360 |
| Auts2 | autism susceptibility candidate 2 | 9,08E-04 | -1,368 |
| Mctp2 | multiple C2 domains, transmembrane 2 | 7,30E-03 | -1,368 |
| Tmem215 | transmembrane protein 215 | 3,16E-04 | -1,389 |
| Fam196a | family with sequence similarity 196, member A | 8,40E-04 | -1,454 |
| Lrrc36 | leucine rich repeat containing 36 | 2,01E-04 | -1,455 |
| Samd14 | sterile alpha motif domain containing 14 | 4,84E-04 | -1,460 |
| T2 | brachyury 2 | 5,44E-04 | -1,475 |
| Clip4 | CAP-GLY domain containing linker protein family, member 4 | 2,69E-02 | -1,477 |
| Fam166a | family with sequence similarity 166, member A | 4,25E-03 | -1,485 |
| Pisd-ps1 | phosphatidylserine decarboxylase, pseudogene 1 | 2,41E-04 | -1,487 |
| Mest | mesoderm specific transcript | 1,46E-02 | -1,516 |
| Gm10796 | predicted gene 10796 | 2,32E-02 | -1,518 |
| Vwa5b1 | von Willebrand factor A domain containing 5B1 | 5,52E-04 | -1,519 |
| Lrrc16b | leucine rich repeat containing 16B | 1,11E-03 | -1,524 |
| Pnet-ps | prenatal ethanol induced mRNA, pseudogene | 1,79E-02 | -1,524 |
| Wdr49 | WD repeat domain 49 | 2,09E-03 | -1,534 |
| Gm609 | predicted gene 609 | 6,82E-04 | -1,544 |
| Fam46d | family with sequence similarity 46, member D | 1,03E-02 | -1,546 |
| Gm11992 | predicted gene 11992 | 1,11E-03 | -1,607 |
| Gm10632 | predicted gene 10632 | 8,18E-04 | -1,651 |
| Gm281 | predicted gene 281 | 1,25E-04 | -1,946 |
