# Supplementary material for: Pancreatic β-Cell Dysfunction in Diet-Induced Obese Mice: Roles of AMP-Kinase, Protein Kinase Cε, Mitochondrial and Cholesterol Metabolism, and Alterations in Gene Expression
Source: PLoS One. 2016 Apr 4;11(4):e0153017. doi: 10.1371/journal.pone.0153017 (PMC4820227; doi:10.1371/journal.pone.0153017)
Supplement: S3 Table — (DOCX) [file pone.0153017.s003.docx]

S3 table. Functional classification of differentially expressed genes in LDR vs ND islets.

| **Gene Symbol** | **Gene description** | **FDR step up (p < 0.05 = 17)** | **Fold-Change Increase: 14 Decrease: 3** |
| --- | --- | --- | --- |
| **Carbohydrate metabolism** | | | |
| Pfkp | phosphofructokinase, platelet | 2,89E-02 | 1,275 |
| **Nucleotide/pyrophosphate metabolism** | | | |
| Gucy2c | guanylate cyclase 2c | 2,89E-02 | 1,812 |
| **Lipid metabolism** | | | |
| Cpt1a | carnitine palmitoyltransferase 1a, liver | 5,29E-04 | 1,521 |
| **Posttranslational modification/ubiquitination/glycosylation** | | | |
| Man1a | mannosidase 1, alpha | 4,61E-02 | 1,200 |
| **Channels and transporters** | | | |
| Kcnh8 | potassium voltage-gated channel, subfamily H (eag-related) | 3,75E-02 | 1,389 |
| Slc4a10 | solute carrier family 4, sodium bicarbonate cotransporter | 1,50E-02 | 1,269 |
| Slc35f1 | solute carrier family 35, member F1 | 3,76E-02 | 1,217 |
| **Hormones/growth factor/Receptors/neuropeptides and exocytosis** | | | |
| Plxna3 | plexin A3 | 1,50E-02 | 1,322 |
| Apln | apelin | 3,00E-02 | -1,211 |
| Npy | neuropeptide Y | 1,50E-02 | -1,972 |
| **AMPK and mTOR pathways** | | | |
| Ppargc1a | peroxisome proliferative activated receptor, gamma, coa | 1,50E-02 | 1,706 |
| **GTPase activity and regulation** | | | |
| Gng4 | guanine nucleotide binding protein (G protein),gamma 4 | 1,50E-02 | 1,212 |
| **Kinases/Phosphatatase and related proteins** | | | |
| Akap6 | A kinase (PRKA) anchor protein 6 | 4,16E-02 | 1,377 |
| **Chemokines/cytokines/adhesion molecules/innate immunity and related proteins** | | | |
| C8b | complement component 8, beta polypeptide | 4,16E-02 | -1,286 |
| **Other functions** | | | |
| Necab2 | N-terminal EF-hand calcium binding protein 2 | 1,90E-02 | 1,397 |
| **Unknown functions** | | | |
| Myo15b | myosin XVB | 3,75E-02 | 1,487 |
| Btnl9 | butyrophilin-like 9 | 3,32E-02 | 1,261 |
