# Supplementary material for: Pancreatic β-Cell Dysfunction in Diet-Induced Obese Mice: Roles of AMP-Kinase, Protein Kinase Cε, Mitochondrial and Cholesterol Metabolism, and Alterations in Gene Expression
Source: PLoS One. 2016 Apr 4;11(4):e0153017. doi: 10.1371/journal.pone.0153017 (PMC4820227; doi:10.1371/journal.pone.0153017)
Supplement: S4 Table — (DOCX) [file pone.0153017.s004.docx]

S4 table. Functional classification of differentially expressed genes in HDR vs LDR islets.

| **Gene symbol** | **Gene description** | **FDR step up (p< 0.05=1041)** | **Fold-Change Increase: 523 Decrease: 518** |
| --- | --- | --- | --- |
| **Carbohydrate metabolism** | | | |
| Gmds | GDP-mannose 4, 6-dehydratase | 1,35E-03 | 1,417 |
| Me3 | malic enzyme 3, NADP(+)-dependent, mitochondrial | 1,27E-03 | 1,397 |
| Mpdu1 | mannose-P-dolichol utilization defect 1 | 3,48E-03 | 1,245 |
| Gla | galactosidase, alpha | 4,54E-03 | 1,229 |
| Bpgm | 2,3-bisphosphoglycerate mutase | 8,97E-03 | 1,221 |
| Pgk1 | phosphoglycerate kinase 1 | 3,51E-03 | 1,215 |
| Galnt9 | UDP-N-acetyl-alpha-D-galactosamine:polypeptide N- Acetylgalactosaminyltransferase 9 | 2,26E-02 | -1,211 |
| Pklr | pyruvate kinase liver and red blood cell | 1,75E-03 | -1,214 |
| Slc2a2 | solute carrier family 2 (facilitated glucose transporter), member 2 | 2,52E-02 | -1,227 |
| Slc2a3 | solute carrier family 2 (facilitated glucose transporter), member 3 | 2,91E-02 | -1,238 |
| Ppp1r1a | protein phosphatase 1, regulatory (inhibitor) subunit 1A | 1,13E-03 | -1,247 |
| Rbp4 | retinol binding protein 4, plasma | 1,24E-02 | -1,253 |
| Eno2 | enolase 2, gamma neuronal | 1,16E-02 | -1,258 |
| Gcnt4 | glucosaminyl (N-acetyl) transferase 4, core 2 (beta-1,6 Acetylglucosaminyltransferase) | 4,45E-03 | -1,274 |
| Khk | ketohexokinase | 4,77E-02 | -1,274 |
| Rpia | ribose 5-phosphate isomerase A | 2,01E-02 | -1,320 |
| Cryl1 | crystallin, lambda 1 | 3,48E-03 | -1,354 |
| Aldoc | aldolase C, fructose-bisphosphate | 2,63E-03 | -1,471 |
| Hpse | heparanase | 2,03E-03 | -1,809 |
| **Glycan metabolism** | | | |
| Alg8 | asparagine-linked glycosylation 8 homolog (yeast, Alpha-1,3-Glucosyltransferase) | 1,05E-02 | 1,342 |
| Dpm3 | dolichyl-phosphate mannosyltransferase polypeptide 3 | 3,78E-03 | 1,328 |
| Alg3 | asparagine-linked glycosylation 3 homolog (yeast, Alpha-1,3-Mannosyltransferase) | 1,14E-02 | 1,310 |
| Glb1 | galactosidase, beta 1 | 2,26E-04 | 1,278 |
| Alg5 | asparagine-linked glycosylation 5 homolog (yeast, Dolichyl-Phosphate Beta-Glucosyltransferase) | 1,84E-03 | 1,277 |
| Alg12 | asparagine-linked glycosylation 12 homolog (yeast, Alpha-1,6-Mannosyltransferase) | 3,08E-03 | 1,241 |
| Pomt1 | protein-O-mannosyltransferase 1 | 4,58E-03 | 1,231 |
| Pomgnt1 | protein O-linked mannose beta1,2-N-acetylglucosaminyltransferase | 1,29E-03 | 1,223 |
| **Amino acid metabolism** | | | |
| Aass | aminoadipate-semialdehyde synthase | 1,46E-03 | 2,296 |
| Pycr1 | pyrroline-5-carboxylate reductase 1 | 1,15E-04 | 1,533 |
| Bcat2 | branched chain aminotransferase 2, mitochondrial | 6,94E-03 | 1,312 |
| Gcdh | glutaryl-Coenzyme A dehydrogenase | 2,11E-03 | 1,292 |
| Bckdhb | branched chain ketoacid dehydrogenase E1, beta polypeptide | 1,15E-04 | 1,263 |
| Ckb | creatine kinase, brain | 1,12E-02 | 1,247 |
| Bckdk | branched chain ketoacid dehydrogenase kinase | 2,18E-03 | 1,242 |
| Ccbl2 | cysteine conjugate-beta lyase 2 | 3,73E-02 | 1,221 |
| Th | tyrosine hydroxylase | 2,38E-02 | -1,347 |
| Gad1 | glutamic acid decarboxylase 1 | 6,05E-03 | -1,641 |
| **Nucleotide/pyrophosphate metabolism** | | | |
| Gucy2c | guanylate cyclase 2c | 5,96E-04 | 1,986 |
| Rrm2 | ribonucleotide reductase M2 | 1,39E-03 | 1,831 |
| Rasl10b | RAS-like, family 10, member B | 1,34E-03 | 1,447 |
| Tyms | thymidylate synthase | 6,07E-03 | 1,434 |
| Dhfr | dihydrofolate reductase | 1,18E-02 | 1,307 |
| Ppa1 | pyrophosphatase (inorganic) 1 | 5,44E-03 | 1,305 |
| Adcy4 | adenylate cyclase 4 | 1,26E-02 | 1,256 |
| Nme2 | Nucleoside Diphosphate Kinase 2 | 1,73E-03 | 1,230 |
| Nme1 | Nucleoside Diphosphate Kinase 1 | 5,02E-03 | 1,209 |
| Dck | deoxycytidine kinase | 3,38E-02 | 1,205 |
| Nme4 | Nucleoside Diphosphate Kinase 4 | 6,53E-04 | -1,242 |
| Nme5 | Nucleoside Diphosphate Kinase 5 | 8,14E-03 | -1,257 |
| **Lipid metabolism** | | | |
| Acsf2 | acyl-CoA synthetase family member 2 | 3,42E-03 | 1,460 |
| Lmf1 | lipase maturation factor 1 | 2,23E-04 | 1,432 |
| Pecr | peroxisomal trans-2-enoyl-CoA reductase | 1,35E-03 | 1,306 |
| Elovl7 | ELOVL family member 7, elongation of long chain fatty acid | 1,35E-03 | 1,271 |
| Thrsp | thyroid hormone responsive SPOT14 homolog (Rattus) | 9,60E-03 | 1,265 |
| Ppapdc1b | phosphatidic acid phosphatase type 2 domain containing 1B | 1,13E-03 | 1,252 |
| Mboat1 | membrane bound O-acyltransferase domain containing 1 | 2,55E-02 | 1,248 |
| Pla2g12a | phospholipase A2, group XIIA | 6,53E-04 | 1,213 |
| Plcd1 | phospholipase C, delta 1 | 7,80E-03 | 1,205 |
| Cyp2j6 | cytochrome P450, family 2, subfamily j, polypeptide 6 | 5,02E-03 | 1,204 |
| Pla2g6 | phospholipase A2, group VI | 1,15E-04 | 1,201 |
| Hsd3b7 | hydroxy-delta-5-steroid dehydrogenase, 3 beta- and steroid Delta isomerase-7 | 1,91E-02 | 1,201 |
| Pikfyve | phosphoinositide kinase, FYVE finger containing | 1,73E-02 | -1,212 |
| Dgat2 | diacylglycerol O-acyltransferase 2 | 3,41E-03 | -1,213 |
| Arv1 | ARV1 homolog (yeast) | 2,25E-03 | -1,214 |
| Pid1 | phosphotyrosine interaction domain containing 1 | 1,05E-02 | -1,217 |
| Aloxe3 | arachidonate lipoxygenase 3 | 2,39E-02 | -1,219 |
| Tmem86b | transmembrane protein 86B | 1,72E-02 | -1,220 |
| Scd2 | stearoyl-Coenzyme A desaturase 2 | 1,02E-03 | -1,227 |
| Acsl3 | acyl-CoA synthetase long-chain family member 3 | 2,96E-03 | -1,243 |
| Acot11 | acyl-CoA thioesterase 11 | 6,11E-03 | -1,258 |
| Vldlr | very low density lipoprotein receptor | 3,30E-03 | -1,268 |
| Sc4mol | sterol-C4-methyl oxidase-like | 4,54E-03 | -1,287 |
| Gpr120 | G protein-coupled receptor 120 | 8,70E-03 | -1,298 |
| Gpd2 | glycerol phosphate dehydrogenase 2, mitochondrial | 1,65E-02 | -1,350 |
| Lpl | lipoprotein lipase | 3,07E-03 | -1,451 |
| Scd1 | stearoyl-Coenzyme A desaturase 1 | 1,54E-02 | -1,569 |
| **Cholesterol metabolism and transport** | | | |
| Apoa2 | apolipoprotein A-II | 1,609E-03 | 1,543 |
| Klb | klotho beta | 2,232E-02 | -1,238 |
| Pcsk9 | proprotein convertase subtilisin/kexin type 9 | 3,319E-03 | -1,295 |
| Srebf2 | sterol regulatory element binding factor 2 | 1,241E-03 | -1,296 |
| Hmgcs1 | 3-hydroxy-3-methylglutaryl-Coenzyme A synthase 1 | 1,19E-02 | -1,305 |
| Idi1 | isopentenyl-diphosphate delta isomerase | 2,042E-02 | -1,310 |
| Stard4 | StAR-related lipid transfer (START) domain containing 4 | 6,261E-04 | -1,330 |
| Dhcr24 | 24-dehydrocholesterol reductase | 1,412E-03 | -1,386 |
| **Metabolism-miscellaneous** | | | |
| Aldh1a3 | aldehyde dehydrogenase family 1, subfamily A3 | 5,49E-04 | 3,493 |
| Gc | group specific component | 5,92E-05 | 1,636 |
| Gsto2 | glutathione S-transferase omega 2 | 4,49E-03 | 1,579 |
| Iyd | iodotyrosine deiodinase | 3,46E-03 | 1,372 |
| Nans | N-acetylneuraminic acid synthase (sialic acid synthase) | 2,09E-03 | 1,338 |
| Bcmo1 | beta-carotene 15,15'-monooxygenase | 3,08E-03 | 1,321 |
| Cmas | cytidine monophospho-N-acetylneuraminic acid synthetase | 1,33E-03 | 1,290 |
| Mosc2 | MOCO sulphurase C-terminal domain containing 2 | 2,62E-03 | 1,289 |
| Dio1 | deiodinase, iodothyronine, type I | 1,13E-03 | 1,254 |
| Gsto1 | glutathione S-transferase omega 1 | 1,28E-02 | 1,243 |
| Nampt | nicotinamide phosphoribosyltransferase | 2,31E-03 | -1,205 |
| Gstm3 | glutathione S-transferase, mu 3 | 1,33E-03 | -1,327 |
| Gstm1 | glutathione S-transferase, mu 1 | 3,05E-03 | -1,427 |
| Ndst4 | N-deacetylase/N-sulfotransferase (heparin glucosaminyl) 4 | 3,30E-03 | -1,747 |
| **Mitochondrial respiration** | | | |
| Ndufa1 | NADH dehydrogenase (ubiquinone) 1 alpha subcomplex, 1 | 5,89E-03 | 1,263 |
| Ndufa4 | NADH dehydrogenase (ubiquinone) 1 alpha subcomplex, 4 | 3,62E-02 | 1,210 |
| **Oxidation-reduction process** | | | |
| Dhrs7 | dehydrogenase/reductase (SDR family) member 7 | 1,84E-03 | 1,275 |
| Dhrs7b | dehydrogenase/reductase (SDR family) member 7B | 7,42E-04 | 1,258 |
| Hsd17b7 | hydroxysteroid (17-beta) dehydrogenase 7 | 8,24E-03 | -1,220 |
| Akr1c19 | aldo-keto reductase family 1, member C19 | 1,35E-02 | -1,267 |
| **Cell cycle** | | | |
| Ccnb1 | cyclin B1 | 1,39E-03 | 3,109 |
| Ccnb2 | cyclin B2 | 5,88E-04 | 2,848 |
| Top2a | topoisomerase (DNA) II alpha | 1,62E-03 | 2,826 |
| Mki67 | antigen identified by monoclonal antibody Ki 67 | 1,13E-03 | 2,727 |
| Plk1 | polo-like kinase 1 (Drosophila) | 2,20E-03 | 2,551 |
| Anln | anillin, actin binding protein | 1,62E-03 | 2,551 |
| Bub1 | budding uninhibited by benzimidazoles 1 homolog (S. cerevisiae) | 2,75E-03 | 2,485 |
| Kif11 | kinesin family member 11 | 1,33E-03 | 2,445 |
| Cks2 | CDC28 protein kinase regulatory subunit 2 | 2,03E-03 | 2,416 |
| Ect2 | ect2 oncogene | 1,45E-03 | 2,383 |
| Ccna2 | cyclin A2 | 1,68E-03 | 2,363 |
| Nek2 | NIMA (never in mitosis gene a)-related expressed kinase 2 | 1,39E-03 | 2,349 |
| Tpx2 | TPX2, microtubule-associated protein homolog (Xenopus laevis) | 1,74E-03 | 2,337 |
| Cdk1 | cyclin-dependent kinase 1 | 6,26E-04 | 2,336 |
| Casc5 | cancer susceptibility candidate 5 | 1,48E-03 | 2,328 |
| D2Ertd750e | DNA segment, Chr 2, ERATO Doi 750, expressed | 9,01E-04 | 2,324 |
| Stmn1 | stathmin 1 | 1,80E-03 | 2,277 |
| Cdc20 | cell division cycle 20 homolog (S. cerevisiae) | 5,96E-04 | 2,234 |
| Nusap1 | nucleolar and spindle associated protein 1 | 1,39E-03 | 2,227 |
| Dtl | denticleless homolog (Drosophila) | 8,88E-04 | 2,181 |
| Prc1 | protein regulator of cytokinesis 1 | 1,93E-03 | 2,138 |
| Dlgap5 | discs, large (Drosophila) homolog-associated protein 5 | 1,44E-03 | 2,073 |
| Cenpf | centromere protein F | 2,20E-03 | 1,970 |
| Aurkb | aurora kinase B | 3,77E-03 | 1,941 |
| Cenpe | centromere protein E | 3,44E-03 | 1,922 |
| C79407 | expressed sequence C79407 | 2,26E-03 | 1,907 |
| Ckap2l | cytoskeleton associated protein 2-like | 2,26E-03 | 1,899 |
| Ckap2 | cytoskeleton associated protein 2 | 1,29E-03 | 1,894 |
| Ncaph | non-SMC condensin I complex, subunit H | 3,10E-03 | 1,883 |
| Nuf2 | NUF2, NDC80 kinetochore complex component, homolog (S. cerevisiae) | 1,33E-03 | 1,869 |
| Rad51 | RAD51 homolog (S. cerevisiae) | 5,65E-03 | 1,824 |
| Sgol2 | shugoshin-like 2 (S. pombe) | 1,35E-03 | 1,821 |
| Kntc1 | kinetochore associated 1 | 1,53E-03 | 1,813 |
| Kif20a | kinesin family member 20A | 4,14E-03 | 1,794 |
| Ncapg | non-SMC condensin I complex, subunit G | 1,61E-03 | 1,785 |
| Aspm | asp (abnormal spindle)-like, microcephaly associated (Drosophila) | 4,49E-03 | 1,764 |
| Cenpm | centromere protein M | 8,86E-04 | 1,756 |
| Kif23 | kinesin family member 23 | 3,51E-03 | 1,742 |
| Aurka | aurora kinase A | 3,51E-03 | 1,735 |
| Cdca3 | cell division cycle associated 3 | 3,19E-03 | 1,713 |
| Cdkn3 | cyclin-dependent kinase inhibitor 3 | 2,26E-03 | 1,708 |
| Spag5 | sperm associated antigen 5 | 3,51E-03 | 1,707 |
| Mcm5 | minichromosome maintenance deficient 5, cell division cycle | 3,19E-03 | 1,702 |
| Zwilch | Zwilch, kinetochore associated, homolog (Drosophila) | 6,90E-04 | 1,694 |
| Foxm1 | forkhead box M1 | 3,09E-03 | 1,686 |
| Bub1b | BUB1 mitotic checkpoint serine/threonine kinase B | 2,27E-03 | 1,674 |
| Cenpa | centromere protein A | 5,69E-03 | 1,673 |
| Mastl | microtubule associated serine/threonine kinase-like | 5,01E-03 | 1,672 |
| Cenpn | centromere protein N | 1,92E-03 | 1,649 |
| Oip5 | Opa interacting protein 5 | 3,02E-03 | 1,648 |
| Fam111a | family with sequence similarity 111, member A | 6,09E-03 | 1,635 |
| Cgref1 | cell growth regulator with EF hand domain 1 | 1,33E-03 | 1,631 |
| Cdca8 | cell division cycle associated 8 | 7,82E-03 | 1,628 |
| Kif4 | kinesin family member 4 | 7,96E-03 | 1,616 |
| E2f8 | E2F transcription factor 8 | 4,47E-03 | 1,603 |
| Cenpk | centromere protein K | 1,41E-03 | 1,600 |
| Spc24 | SPC24, NDC80 kinetochore complex component, homolog (S. cerevisiae) | 1,24E-03 | 1,561 |
| Cdca2 | cell division cycle associated 2 | 3,04E-03 | 1,555 |
| Kif20b | kinesin family member 20B | 2,80E-03 | 1,554 |
| Cdc25c | cell division cycle 25 homolog C (S. pombe) | 2,98E-03 | 1,547 |
| Racgap1 | Rac GTPase-activating protein 1 | 1,90E-03 | 1,546 |
| Ncapg2 | non-SMC condensin II complex, subunit G2 | 1,03E-02 | 1,544 |
| Melk | maternal embryonic leucine zipper kinase | 2,73E-03 | 1,531 |
| Hells | helicase, lymphoid specific | 5,74E-03 | 1,525 |
| Ccnf | cyclin F | 8,17E-03 | 1,523 |
| Mcm2 | minichromosome maintenance deficient 2 mitotin (S. cerevisiae) | 7,09E-03 | 1,522 |
| Kif2c | kinesin family member 2C | 7,19E-03 | 1,511 |
| Ttk | Ttk protein kinase | 3,13E-03 | 1,507 |
| Cenpi | centromere protein I | 7,19E-03 | 1,494 |
| Cks1b | CDC28 protein kinase 1b | 3,08E-03 | 1,491 |
| E2f1 | E2F transcription factor 1 | 3,69E-04 | 1,481 |
| Smc2 | structural maintenance of chromosomes 2 | 1,03E-02 | 1,467 |
| Mcm6 | minichromosome maintenance deficient 6 (MIS5 homolog, S. pombe) | 1,71E-02 | 1,462 |
| Cdca5 | cell division cycle associated 5 | 5,19E-03 | 1,460 |
| Mcm3 | minichromosome maintenance deficient 3 (S. cerevisiae) | 2,14E-02 | 1,459 |
| Ndc80 | NDC80 homolog, kinetochore complex component | 1,59E-02 | 1,455 |
| Kif22 | kinesin family member 22 | 5,95E-03 | 1,454 |
| Fam64a | family with sequence similarity 64, member A | 7,53E-03 | 1,446 |
| Ncapd2 | non-SMC condensin I complex, subunit D2 | 1,11E-02 | 1,444 |
| Cdt1 | chromatin licensing and DNA replication factor 1 | 1,01E-02 | 1,431 |
| Clspn | claspin homolog (Xenopus laevis) | 3,24E-03 | 1,413 |
| Cdkn2c | cyclin-dependent kinase inhibitor 2C (p18, inhibits CDK4) | 1,79E-03 | 1,410 |
| Kif15 | kinesin family member 15 | 2,06E-03 | 1,408 |
| Mad2l1 | MAD2 mitotic arrest deficient-like 1 (yeast) | 3,02E-02 | 1,407 |
| Sgol1 | shugoshin-like 1 (S. pombe) | 9,54E-03 | 1,406 |
| Cdc6 | cell division cycle 6 homolog (S. cerevisiae) | 1,58E-03 | 1,389 |
| Cenph | centromere protein H | 2,40E-02 | 1,380 |
| Chaf1b | chromatin assembly factor 1, subunit B (p60) | 2,34E-02 | 1,372 |
| Dbf4 | DBF4 homolog (S. cerevisiae) | 9,77E-03 | 1,371 |
| Uhrf1 | ubiquitin-like, containing PHD and RING finger domains, 1 | 1,46E-02 | 1,370 |
| Fam83d | family with sequence similarity 83, member D | 2,45E-03 | 1,364 |
| Mcm10 | minichromosome maintenance deficient 10 (S. cerevisiae) | 9,86E-03 | 1,362 |
| Dsn1 | DSN1, MIND kinetochore complex component, homolog (S. cerevisiae) | 1,50E-02 | 1,351 |
| Kif18a | kinesin family member 18A | 3,79E-02 | 1,346 |
| Mcm8 | minichromosome maintenance deficient 8 (S. cerevisiae) | 9,97E-03 | 1,338 |
| Mcm7 | minichromosome maintenance deficient 7 (S. cerevisiae) | 2,32E-02 | 1,318 |
| E2f7 | E2F transcription factor 7 | 7,37E-03 | 1,310 |
| Cep55 | centrosomal protein 55 | 5,99E-03 | 1,305 |
| Pkmyt1 | protein kinase, membrane associated tyrosine/threonine 1 | 2,77E-03 | 1,292 |
| Arhgef10 | Rho guanine nucleotide exchange factor (GEF) 10 | 2,49E-03 | 1,290 |
| Gins1 | GINS complex subunit 1 (Psf1 homolog) | 1,30E-02 | 1,279 |
| Smc4 | structural maintenance of chromosomes 4 | 2,15E-02 | 1,272 |
| Cdc45 | cell division cycle 45 homolog (S. cerevisiae) | 6,47E-03 | 1,272 |
| Espl1 | extra spindle poles-like 1 (S. cerevisiae) | 1,71E-02 | 1,262 |
| Reg3g | regenerating islet-derived 3 gamma | 2,38E-02 | 1,250 |
| Cenpp | centromere protein P | 4,77E-02 | 1,242 |
| Plk4 | polo-like kinase 4 (Drosophila) | 3,94E-02 | 1,237 |
| Nsl1 | NSL1, MIND kinetochore complex component, homolog (S. cerevisiae) | 8,70E-03 | 1,237 |
| Haus4 | HAUS augmin-like complex, subunit 4 | 2,30E-02 | 1,229 |
| Pmf1 | polyamine-modulated factor 1 | 7,23E-03 | 1,227 |
| Chaf1a | chromatin assembly factor 1, subunit A (p150) | 3,30E-02 | 1,218 |
| Jtb | jumping translocation breakpoint | 6,26E-04 | 1,206 |
| S100a4 | S100 calcium binding protein A4 | 2,98E-03 | 1,200 |
| Eapp | E2F-associated phosphoprotein | 5,37E-03 | -1,214 |
| Fam190a | family with sequence similarity 190, member A | 2,47E-03 | -1,228 |
| Dcaf12l1 | DDB1 and CUL4 associated factor 12-like 1 | 3,28E-03 | -1,231 |
| Klhl13 | kelch-like 13 (Drosophila) | 2,10E-02 | -1,241 |
| Chd7 | chromodomain helicase DNA binding protein 7 | 1,50E-02 | -1,301 |
| Ttc28 | tetratricopeptide repeat domain 28 | 3,83E-03 | -1,350 |
| Phf16 | PHD finger protein 16 | 1,33E-03 | -1,370 |
| **DNA repair/DNA recombination/DNA replication** | | | |
| Asf1b | ASF1 anti-silencing function 1 homolog B (S. cerevisiae) | 1,33E-03 | 2,400 |
| Pole | polymerase (DNA directed), epsilon | 2,20E-03 | 1,814 |
| Neil3 | nei like 3 (E. coli) | 3,90E-03 | 1,698 |
| Fignl1 | fidgetin-like 1 | 1,33E-03 | 1,686 |
| Fen1 | flap structure specific endonuclease 1 | 5,69E-03 | 1,531 |
| Fancd2 | Fanconi anemia, complementation group D2 | 8,04E-03 | 1,448 |
| Brip1 | BRCA1 interacting protein C-terminal helicase 1 | 1,61E-03 | 1,396 |
| Pole2 | polymerase (DNA directed), epsilon 2 (p59 subunit) | 4,07E-03 | 1,384 |
| Fancb | Fanconi anemia, complementation group B | 9,91E-03 | 1,381 |
| Brca1 | breast cancer 1 | 1,08E-02 | 1,371 |
| Rad51ap1 | RAD51 associated protein 1 | 1,34E-03 | 1,370 |
| Fanca | Fanconi anemia, complementation group A | 5,28E-03 | 1,364 |
| Exo1 | exonuclease 1 | 6,20E-03 | 1,359 |
| Fanci | Fanconi anemia, complementation group I | 7,29E-03 | 1,352 |
| Gins2 | GINS complex subunit 2 (Psf2 homolog) | 1,10E-02 | 1,349 |
| Rrm1 | ribonucleotide reductase M1 | 2,28E-02 | 1,332 |
| Rfc4 | replication factor C (activator 1) 4 | 1,39E-02 | 1,322 |
| Rpa2 | replication protein A2 | 3,72E-03 | 1,302 |
| Hmgb2 | high mobility group box 2 | 4,68E-02 | 1,298 |
| Rad18 | RAD18 homolog (S. cerevisiae) | 1,29E-02 | 1,297 |
| Rad54b | RAD54 homolog B (S. cerevisiae) | 1,86E-02 | 1,254 |
| Rexo2 | REX2, RNA exonuclease 2 homolog (S. cerevisiae) | 5,28E-03 | 1,228 |
| Dna2 | DNA replication helicase 2 homolog (yeast) | 2,56E-02 | 1,222 |
| Ogg1 | 8-oxoguanine DNA-glycosylase 1 | 3,07E-03 | 1,218 |
| Topbp1 | topoisomerase (DNA) II binding protein 1 | 2,50E-02 | 1,211 |
| Rad54l | RAD54 like (S. cerevisiae) | 1,16E-02 | 1,208 |
| Rfc5 | replication factor C (activator 1) 5 | 2,98E-02 | 1,207 |
| Ercc6l | excision repair cross-complementing rodent repair Deficiency,Complementation Group 6-Like | 2,23E-02 | 1,207 |
| Poll | polymerase (DNA directed), lambda | 2,18E-02 | 1,206 |
| Dclre1c | DNA cross-link repair 1C, PSO2 homolog (S. cerevisiae) | 1,59E-02 | -1,204 |
| Msh2 | mutS homolog 2 (E. coli) | 1,13E-02 | -1,207 |
| Rev1 | REV1 homolog (S. cerevisiae) | 2,73E-03 | -1,239 |
| Cdc14b | CDC14 cell division cycle 14 homolog B (S. cerevisiae) | 1,34E-03 | -1,243 |
| Rad9b | RAD9 homolog B (S. cerevisiae) | 1,55E-03 | -1,243 |
| Rdm1 | RAD52 motif 1 | 3,86E-03 | -1,312 |
| **Nucleosome assembly** | | | |
| Cenpw | centromere protein W | 3,04E-02 | 1,249 |
| Hist1h1a | histone cluster 1, H1a | 3,63E-02 | 1,284 |
| Hist1h1b | histone cluster 1, H1b | 1,25E-02 | 1,440 |
| Hist1h2ab | histone cluster 1, H2ab | 2,31E-02 | 1,481 |
| Hist1h2af | histone cluster 1, H2af | 8,82E-03 | 1,205 |
| Hist1h2ag | histone cluster 1, H2ag | 2,78E-02 | 1,205 |
| Hist1h2ah | histone cluster 1, H2ah | 7,61E-03 | 1,205 |
| Hist1h2ak | histone cluster 1, H2ak | 3,70E-02 | 1,265 |
| Hist1h2an | histone cluster 1, H2an | 8,25E-03 | 1,225 |
| Hist1h2ao | histone cluster 1, H2ao | 6,78E-03 | 1,211 |
| Hist1h2bb | histone cluster 1, H2bb | 1,70E-02 | 1,541 |
| Hist1h2bh | histone cluster 1, H2bh | 2,21E-02 | 1,535 |
| Hist1h3b | histone cluster 1, H3b | 1,55E-03 | 1,273 |
| Hist1h3c | histone cluster 1, H3c | 1,61E-03 | 1,280 |
| Hist1h3d | histone cluster 1, H3d | 1,90E-03 | 1,269 |
| Hist1h3e | histone cluster 1, H3e | 1,81E-03 | 1,271 |
| Hist1h3g | histone cluster 1, H3g | 1,73E-03 | 1,276 |
| Hist1h3h | histone cluster 1, H3h | 1,62E-03 | 1,267 |
| Hist1h3i | histone cluster 1, H3i | 1,93E-03 | 1,285 |
| Hist2h2bb | histone cluster 2, H2bb | 5,84E-03 | 1,314 |
| Hist2h3b | histone cluster 2, H3b | 2,40E-03 | 1,286 |
| **Epigenic regulation** | | | |
| Esco2 | establishment of cohesion 1 homolog 2 (S. cerevisiae) | 5,95E-03 | 1,831 |
| Rbm15b | RNA binding motif protein 15B | 7,26E-03 | 1,205 |
| Prdm11 | PR domain containing 11 | 7,08E-03 | -1,202 |
| Mll2 | myeloid/lymphoid or mixed-lineage leukemia 2 | 2,30E-02 | -1,208 |
| Eif2c1 | eukaryotic translation initiation factor 2C, 1 | 1,73E-03 | -1,209 |
| Phc3 | polyhomeotic-like 3 (Drosophila) | 2,83E-02 | -1,217 |
| Tet3 | tet oncogene family member 3 | 1,84E-02 | -1,248 |
| Mll3 | myeloid/lymphoid or mixed-lineage leukemia 3 | 2,71E-02 | -1,268 |
| A1cf | APOBEC1 complementation factor | 8,73E-03 | -1,278 |
| Dnmt3a | DNA methyltransferase 3A | 2,65E-03 | -1,280 |
| Cbx7 | chromobox homolog 7 | 4,51E-03 | -1,321 |
| Kdm6b | KDM1 lysine (K)-specific demethylase 6B | 2,26E-04 | -1,321 |
| Tet2 | tet oncogene family member 2 | 7,19E-03 | -1,348 |
| Phf15 | PHD finger protein 15 | 2,49E-03 | -1,427 |
| Tet1 | tet oncogene 1 | 1,39E-02 | -1,436 |
| **Apoptosis** | | | |
| Dapl1 | death associated protein-like 1 | 2,26E-04 | 1,778 |
| Stk17b | serine/threonine kinase 17b (apoptosis-inducing) | 1,69E-03 | 1,385 |
| Birc5 | baculoviral IAP repeat-containing 5 | 8,21E-03 | 1,370 |
| Clptm1l | CLPTM1-like | 1,33E-03 | 1,347 |
| Atp6v1g2 | ATPase, H+ transporting, lysosomal V1 subunit G2 | 2,26E-04 | 1,319 |
| Tpd52l1 | tumor protein D52-like 1 | 3,74E-03 | 1,286 |
| Plekhf1 | pleckstrin homology domain containing, family F (with FYVE domain) member 1 | 5,74E-03 | 1,229 |
| Lgals12 | lectin, galactose binding, soluble 12 | 1,91E-02 | 1,227 |
| Clu | clusterin | 7,92E-03 | 1,214 |
| Trim35 | tripartite motif-containing 35 | 1,79E-03 | -1,217 |
| Rnf122 | ring finger protein 122 | 4,46E-02 | -1,217 |
| Syngap1 | synaptic Ras GTPase activating protein 1 homolog (rat) | 6,04E-03 | -1,220 |
| Tnfaip8 | tumor necrosis factor, alpha-induced protein 8 | 2,10E-02 | -1,232 |
| Pycard | PYD and CARD domain containing | 4,06E-03 | -1,257 |
| Aatk | apoptosis-associated tyrosine kinase | 6,83E-03 | -1,257 |
| Robo2 | roundabout homolog 2 (Drosophila) | 8,21E-03 | -1,289 |
| Robo1 | roundabout homolog 1 (Drosophila) | 1,39E-03 | -1,295 |
| Ank2 | ankyrin 2, brain | 1,81E-03 | -1,341 |
| Bcl2l11 | BCL2-like 11 (apoptosis facilitator) | 1,58E-03 | -1,348 |
| Serpinb9 | serine (or cysteine) peptidase inhibitor, clade B, member 9 | 1,11E-02 | -1,351 |
| Unc5c | unc-5 homolog C (C. elegans) | 7,90E-03 | -1,455 |
| Amigo2 | adhesion molecule with Ig like domain 2 | 1,35E-03 | -1,484 |
| **Oxidative stress/DNA damage response** | | | |
| Gpx2 | glutathione peroxidase 2 | 4,64E-03 | 1,496 |
| Chek1 | checkpoint kinase 1 homolog (S. pombe) | 1,98E-03 | 1,461 |
| Prdx4 | peroxiredoxin 4 | 8,59E-03 | 1,378 |
| Gtse1 | G two S phase expressed protein 1 | 7,98E-03 | 1,355 |
| Osgin1 | oxidative stress induced growth inhibitor 1 | 9,17E-03 | 1,345 |
| Tacc3 | transforming, acidic coiled-coil containing protein 3 | 1,03E-02 | 1,325 |
| Phlda3 | pleckstrin homology-like domain, family A, member 3 | 1,31E-02 | 1,225 |
| Ppp2r5c | protein phosphatase 2, regulatory subunit B (B56), gamma | 1,77E-05 | 1,222 |
| Txnrd3 | thioredoxin reductase 3 | 4,81E-02 | -1,232 |
| Atm | ataxia telangiectasia mutated homolog (human) | 3,91E-02 | -1,243 |
| **Transcription Factors** | | | |
| Tcf19 | transcription factor 19 | 1,37E-03 | 1,823 |
| Mybl1 | myeloblastosis oncogene-like 1 | 2,67E-02 | 1,358 |
| Etv5 | ets variant gene 5 | 3,83E-02 | 1,288 |
| Bach2 | BTB and CNC homology 2 | 4,51E-03 | 1,235 |
| Carf | calcium response factor | 4,96E-02 | -1,201 |
| Prdm2 | PR domain containing 2, with ZNF domain | 1,16E-02 | -1,217 |
| Elf4 | E74-like factor 4 (ets domain transcription factor) | 4,19E-02 | -1,218 |
| Dach1 | dachshund 1 (Drosophila) | 6,31E-03 | -1,222 |
| Arid3b | AT rich interactive domain 3B (BRIGHT-like) | 6,59E-03 | -1,238 |
| Hivep3 | human immunodeficiency virus type I enhancer binding protein 3 | 7,05E-03 | -1,251 |
| Nfat5 | nuclear factor of activated T-cells 5 | 8,55E-03 | -1,251 |
| Mlxipl | MLX interacting protein-like | 1,85E-02 | -1,265 |
| Arx | aristaless related homeobox | 3,05E-03 | -1,275 |
| Hlf | hepatic leukemia factor | 6,27E-03 | -1,275 |
| Myt1 | myelin transcription factor 1 | 1,65E-02 | -1,278 |
| Hhex | hematopoietically expressed homeobox | 3,20E-02 | -1,283 |
| Pou3f4 | POU domain, class 3, transcription factor 4 | 8,67E-03 | -1,374 |
| Mnx1 | motor neuron and pancreas homeobox 1 | 3,56E-03 | -1,406 |
| Mafb | v-maf musculoaponeurotic fibrosarcoma oncogene homolog B | 4,18E-02 | -1,418 |
| Per3 | period homolog 3 (Drosophila) | 2,15E-03 | -1,592 |
| Plag1 | pleiomorphic adenoma gene 1 | 1,49E-03 | -1,613 |
| Nr1d1 | nuclear receptor subfamily 1, group D, member 1 | 1,85E-02 | -1,636 |
| **Nuclear receptor and related proteins** | | | |
| Nr4a2 | nuclear receptor subfamily 4, group A, member 2 | 4,36E-03 | 1,342 |
| Ncoa1 | nuclear receptor coactivator 1 | 1,86E-03 | -1,219 |
| Thra | thyroid hormone receptor alpha | 4,04E-03 | -1,291 |
| Nr3c2 | nuclear receptor subfamily 3, group C, member 2 | 1,53E-03 | -1,383 |
| **Transcription regulation/alternative splicing** | | | |
| Zfp367 | zinc finger protein 367 | 4,71E-03 | 1,445 |
| Atad2 | ATPase family, AAA domain containing 2 | 2,61E-03 | 1,376 |
| Ezh2 | enhancer of zeste homolog 2 (Drosophila) | 2,06E-03 | 1,343 |
| Carhsp1 | calcium regulated heat stable protein 1 | 5,42E-03 | 1,317 |
| Ldb2 | LIM domain binding 2 | 1,30E-02 | 1,283 |
| Wdhd1 | WD repeat and HMG-box DNA binding protein 1 | 3,27E-02 | 1,281 |
| Gemin6 | gem (nuclear organelle) associated protein 6 | 3,83E-03 | 1,280 |
| Hey1 | hairy/enhancer-of-split related with YRPW motif 1 | 4,08E-02 | 1,257 |
| Zcchc12 | zinc finger, CCHC domain containing 12 | 4,52E-02 | 1,250 |
| Zfp57 | zinc finger protein 57 | 5,74E-03 | 1,227 |
| Mbnl3 | muscleblind-like 3 (Drosophila) | 3,53E-02 | 1,213 |
| Parn | poly(A)-specific ribonuclease (deadenylation nuclease) | 7,66E-03 | 1,208 |
| Armcx3 | armadillo repeat containing, X-linked 3 | 5,25E-03 | 1,206 |
| Srp9 | signal recognition particle 9 | 1,60E-03 | 1,206 |
| Rnaseh2c | ribonuclease H2, subunit C | 2,25E-02 | 1,204 |
| Srsf7 | serine/arginine-rich splicing factor 7 | 1,78E-03 | -1,202 |
| Tnrc6a | trinucleotide repeat containing 6a | 2,02E-02 | -1,203 |
| Ppargc1b | peroxisome proliferative activated receptor, gamma, coactivator 1 beta | 4,78E-03 | -1,205 |
| Tle3 | transducin-like enhancer of split 3, homolog Of Drosophila E(Sp1) | 2,94E-03 | -1,206 |
| Bmi1 | Bmi1 polycomb ring finger oncogene | 3,51E-03 | -1,210 |
| Srfbp1 | serum response factor binding protein 1 | 9,17E-03 | -1,210 |
| Rbm27 | RNA binding motif protein 27 | 1,18E-04 | -1,210 |
| Ssbp2 | single-stranded DNA binding protein 2 | 2,65E-03 | -1,213 |
| Sin3a | transcriptional regulator, SIN3A (yeast) | 3,99E-03 | -1,213 |
| Prpf39 | PRP39 pre-mRNA processing factor 39 homolog (yeast) | 3,87E-02 | -1,214 |
| Zfp455 | zinc finger protein 455 | 7,96E-03 | -1,217 |
| Rcor3 | REST corepressor 3 | 1,43E-02 | -1,217 |
| Polr3g | polymerase (RNA) III (DNA directed) polypeptide G | 2,67E-02 | -1,219 |
| Chd6 | chromodomain helicase DNA binding protein 6 | 1,61E-02 | -1,221 |
| Bat2l | HLA-B associated transcript 2-like | 2,30E-03 | -1,221 |
| Zfp583 | zinc finger protein 583 | 4,85E-03 | -1,226 |
| Prox1 | prospero-related homeobox 1 | 1,61E-03 | -1,227 |
| Bcorl1 | BCL6 co-repressor-like 1 | 1,39E-03 | -1,231 |
| Ankrd12 | ankyrin repeat domain 12 | 8,26E-03 | -1,238 |
| Zfp654 | zinc finger protein 654 | 1,23E-02 | -1,240 |
| Bcor | BCL6 interacting corepressor | 5,49E-03 | -1,243 |
| Zbtb10 | zinc finger and BTB domain containing 10 | 3,00E-02 | -1,246 |
| Zfp28 | zinc finger protein 28 | 3,64E-03 | -1,252 |
| Cstf3 | cleavage stimulation factor, 3' pre-RNA, subunit 3 | 3,79E-02 | -1,253 |
| Elp4 | elongation protein 4 homolog (S. cerevisiae) | 1,81E-03 | -1,254 |
| Zfp317 | zinc finger protein 317 | 7,53E-03 | -1,255 |
| Zfp2 | zinc finger protein 2 | 1,44E-03 | -1,257 |
| Tra2a | transformer 2 alpha homolog (Drosophila) | 4,29E-02 | -1,258 |
| Klf7 | Kruppel-like factor 7 (ubiquitous) | 9,29E-03 | -1,260 |
| Zfp618 | zinc fingerprotein 618 | 4,36E-03 | -1,265 |
| Ikzf4 | IKAROS family zinc finger 4 | 1,42E-02 | -1,275 |
| Mov10 | Moloney leukemia virus 10 | 1,13E-03 | -1,275 |
| Irx2 | Iroquois related homeobox 2 (Drosophila) | 4,32E-02 | -1,278 |
| Cdx4 | caudal type homeobox 4 | 4,15E-02 | -1,279 |
| Irx1 | Iroquois related homeobox 1 (Drosophila) | 4,57E-02 | -1,285 |
| Chd3 | chromodomain helicase DNA binding protein 3 | 6,26E-04 | -1,289 |
| Zfp398 | zinc finger protein 398 | 4,01E-03 | -1,291 |
| Mll1 | myeloid/lymphoid or mixed-lineage leukemia 1 | 2,11E-02 | -1,292 |
| Pou6f2 | POU domain, class 6, transcription factor 2 | 4,30E-02 | -1,300 |
| Sp4 | trans-acting transcription factor 4 | 3,45E-03 | -1,314 |
| Tef | thyrotroph embryonic factor | 4,95E-03 | -1,325 |
| Rcor2 | REST corepressor 2 | 1,23E-02 | -1,340 |
| Prdm1 | PR domain containing 1, with ZNF domain | 2,51E-02 | -1,351 |
| Klhl3 | kelch-like 3 (Drosophila) | 1,97E-03 | -1,363 |
| Zbtb20 | zinc finger and BTB domain containing 20 | 5,00E-03 | -1,364 |
| Mamld1 | mastermind-like domain containing 1 | 6,70E-03 | -1,372 |
| Chd5 | chromodomain helicase DNA binding protein 5 | 1,39E-03 | -1,385 |
| Zkscan16 | zinc finger with KRAB and SCAN domains 16 | 7,61E-03 | -1,401 |
| Bhlhe41 | basic helix-loop-helix family, member e41 | 5,29E-04 | -1,407 |
| Msi1 | Musashi homolog 1(Drosophila) | 6,51E-03 | -1,448 |
| Zim1 | zinc finger, imprinted 1 | 1,52E-03 | -1,559 |
| Dbp | D site albumin promoter binding protein | 2,95E-03 | -1,760 |
| **Protein synthesis/translation regulation/protein folding/endoplasmic reticulum stress** | | | |
| Derl3 | Der1-like domain family, member 3 | 1,28E-04 | 2,300 |
| Kdelr3 | KDEL (Lys-Asp-Glu-Leu) endoplasmic reticulum protein retention receptor 3 | 5,01E-04 | 2,208 |
| Fkbp11 | FK506 binding protein 11 | 1,15E-04 | 1,528 |
| Fam129a | family with sequence similarity 129, member A | 2,06E-02 | 1,423 |
| Dnajb11 | DnaJ (Hsp40) homolog, subfamily B, member 11 | 2,32E-03 | 1,403 |
| Sec11c | SEC11 homolog C (S. cerevisiae) | 9,62E-04 | 1,379 |
| Spcs1 | signal peptidase complex subunit 1 homolog | 5,55E-03 | 1,350 |
| Prkcsh | protein kinase C substrate 80K-H | 6,67E-04 | 1,346 |
| Qsox1 | quiescin Q6 sulfhydryl oxidase 1 | 1,63E-03 | 1,321 |
| Pdrg1 | p53 and DNA damage regulated 1 | 1,43E-03 | 1,310 |
| Ppib | peptidylprolyl isomerase B | 2,07E-03 | 1,281 |
| Paip2b | poly(A) binding protein interacting protein 2B | 1,28E-04 | 1,276 |
| Pdia4 | protein disulfide isomerase associated 4 | 8,85E-03 | 1,271 |
| Pdia6 | protein disulfide isomerase associated 6 | 1,32E-03 | 1,270 |
| Golm1 | golgi membrane protein 1 | 2,40E-03 | 1,265 |
| Edem2 | ER degradation enhancer, mannosidase alpha-like 2 | 1,97E-03 | 1,261 |
| Nucb1 | nucleobindin 1 | 3,69E-04 | 1,247 |
| Pacrg | PARK2 co-regulated | 1,22E-02 | 1,245 |
| Hspb6 | heat shock protein, alpha-crystallin-related, B6 | 4,64E-02 | 1,244 |
| Os9 | amplified in osteosarcoma | 2,26E-04 | 1,242 |
| Rps8 | ribosomal protein S8 | 2,47E-03 | 1,239 |
| Edem1 | ER degradation enhancer, mannosidase alpha-like 1 | 1,62E-03 | 1,227 |
| Dnajc3 | DnaJ (Hsp40) homolog, subfamily C, member 3 | 1,18E-03 | 1,214 |
| H47 | histocompatibility 47 | 1,18E-03 | 1,207 |
| Manf | mesencephalic astrocyte-derived neurotrophic factor | 4,48E-03 | 1,207 |
| Ssr3 | signal sequence receptor, gamma | 3,69E-04 | 1,205 |
| Mrps12 | mitochondrial ribosomal protein S12 | 2,44E-02 | 1,200 |
| Utp15 | UTP15, U3 small nucleolar ribonucleoprotein, homolog (yeast) | 8,04E-03 | -1,204 |
| Zc3h3 | zinc finger CCCH type containing 3 | 2,43E-02 | -1,206 |
| Sez6l | seizure related 6 homolog like | 5,02E-03 | -1,210 |
| Rpl23a | ribosomal protein L23a | 4,01E-03 | -1,287 |
| Igf2bp2 | insulin-like growth factor 2 mRNA binding protein 2 | 1,62E-03 | -1,348 |
| Macrod2 | MACRO domain containing 2 | 1,39E-02 | -1,356 |
| Hspa12a | heat shock protein 12A | 2,38E-03 | -1,362 |
| Ppil6 | peptidylprolyl isomerase (cyclophilin)-like 6 | 1,72E-03 | -1,497 |
| **Vesicle transport/Protein trafficking** | | | |
| Rab18 | RAB18, member RAS oncogene family | 9,32E-03 | 1,477 |
| Copz2 | coatomer protein complex, subunit zeta 2 | 1,15E-04 | 1,452 |
| Sytl1 | synaptotagmin-like 1 | 5,07E-03 | 1,330 |
| Tmed3 | transmembrane emp24 domain containing 3 | 6,53E-04 | 1,314 |
| Ssr4 | signal sequence receptor, delta | 1,57E-04 | 1,290 |
| Sec16b | SEC16 homolog B (S. cerevisiae) | 3,45E-03 | 1,239 |
| Srprb | signal recognition particle receptor, B subunit | 1,64E-03 | 1,236 |
| Mall | mal, T-cell differentiation protein-like | 3,69E-02 | 1,234 |
| Slc18a1 | solute carrier family 18 (vesicular monoamine), member 1 | 2,20E-03 | 1,230 |
| Plvap | plasmalemma vesicle associated protein | 1,23E-02 | 1,202 |
| Erp29 | endoplasmic reticulum protein 29 | 1,67E-02 | 1,200 |
| Syt14 | synaptotagmin XIV | 1,61E-03 | -1,206 |
| Vps13c | vacuolar protein sorting 13C (yeast) | 1,56E-02 | -1,223 |
| Vps13d | vacuolar protein sorting 13 D (yeast) | 1,22E-02 | -1,231 |
| Pclo | piccolo (presynaptic cytomatrix protein) | 1,49E-02 | -1,281 |
| Cabp7 | calcium binding protein 7 | 3,48E-03 | -1,284 |
| Rab3b | RAB3B, member RAS oncogene family | 6,73E-03 | -1,285 |
| Kalrn | kalirin, RhoGEF kinase | 1,96E-03 | -1,421 |
| **Postranslational modification/ubiquination/glycosylation** | | | |
| Ube2c | ubiquitin-conjugating enzyme E2C | 4,32E-04 | 2,357 |
| Trim59 | tripartite motif-containing 59 | 1,84E-02 | 1,592 |
| Mogs | mannosyl-oligosaccharide glucosidase | 5,37E-03 | 1,343 |
| Otud7a | OTU domain containing 7A | 3,40E-03 | 1,341 |
| Asb9 | ankyrin repeat and SOCS box-containing 9 | 5,28E-03 | 1,332 |
| Gyltl1b | glycosyltransferase-like 1B | 9,23E-03 | 1,294 |
| Bard1 | BRCA1 associated RING domain 1 | 1,05E-02 | 1,290 |
| Qpctl | glutaminyl-peptide cyclotransferase-like | 7,05E-04 | 1,286 |
| Rnf215 | ring finger protein 215 | 1,39E-03 | 1,249 |
| Man1a | mannosidase 1, alpha | 2,15E-03 | 1,214 |
| Ube2s | ubiquitin-conjugating enzyme E2S | 1,62E-03 | 1,209 |
| Krtcap2 | keratinocyte associated protein 2 | 7,96E-03 | 1,208 |
| Uba5 | ubiquitin-like modifier activating enzyme 5 | 7,50E-03 | 1,204 |
| Dpagt1 | dolichyl-phosphate (UDP-N-acetylglucosamine) N-acetylglucosaminephosphotransferase 1 | 1,71E-02 | 1,203 |
| Rpn2 | ribophorin II | 2,34E-04 | 1,202 |
| Parp9 | poly (ADP-ribose) polymerase family, member 9 | 6,21E-03 | -1,205 |
| Setd2 | SET domain containing 2 | 7,60E-03 | -1,205 |
| Rnf43 | ring finger protein 43 | 1,91E-03 | -1,211 |
| Herc3 | hect domain and RLD 3 | 6,90E-03 | -1,211 |
| Dtx3l | deltex 3-like (Drosophila) | 3,69E-04 | -1,221 |
| Rimklb | ribosomal modification protein rimK-like family member B | 1,70E-02 | -1,223 |
| March4 | membrane-associated ring finger (C3HC4) 4 | 1,62E-03 | -1,223 |
| Mdm4 | transformed mouse 3T3 cell double minute 4 | 5,84E-03 | -1,229 |
| Fbxl12 | F-box and leucine-rich repeat protein 12 | 5,92E-03 | -1,233 |
| Rbbp6 | retinoblastoma binding protein 6 | 1,02E-03 | -1,236 |
| Rnf208 | ring finger protein 208 | 4,67E-02 | -1,248 |
| Zdhhc23 | zinc finger, DHHC domain containing 23 | 1,42E-02 | -1,252 |
| Uba7 | ubiquitin-like modifier activating enzyme 7 | 5,49E-04 | -1,292 |
| Galnt14 | UDP-N-acetyl-alpha-D-galactosamine: Polypeptide  N-Acetylgalactosaminyltransferase 14 | 1,33E-03 | -1,331 |
| Parp14 | poly (ADP-ribose) polymerase family, member 14 | 6,57E-03 | -1,339 |
| Herc6 | hect domain and RLD 6 | 1,84E-03 | -1,341 |
| Galntl4 | UDP-N-acetyl-alpha-D-galactosamine: Polypeptide  N-Acetylgalactosaminyltransferase-Like 4 | 2,81E-03 | -1,420 |
| Galnt13 | UDP-N-acetyl-alpha-D-galactosamine: Polypeptide  N-Acetylgalactosaminyltransferase 13 | 1,42E-03 | -1,429 |
| **Proteosome/lysosome/autophagy/phagocytosis** | | | |
| Wipi1 | WD repeat domain, phosphoinositide interacting 1 | 1,57E-03 | 1,256 |
| Ift88 | intraflagellar transport 88 homolog (Chlamydomonas) | 2,34E-03 | -1,231 |
| Wdfy3 | WD repeat and FYVE domain containing 3 | 7,59E-03 | -1,232 |
| Vps13a | vacuolar protein sorting 13A (yeast) | 8,49E-03 | -1,239 |
| Dnajc13 | DnaJ (Hsp40) homolog, subfamily C, member 13 | 9,48E-03 | -1,240 |
| Mcoln3 | mucolipin 3 | 1,11E-03 | -1,770 |
| **Peptidase/protease and related inhibitors** | | | |
| Serpina7 | serine (or cysteine) peptidase inhibitor, Clade A (Alpha-1 Antiproteinase, Antitrypsin), Member 7 | 8,64E-04 | 2,515 |
| Pappa2 | pappalysin 2 | 1,35E-03 | 1,584 |
| Prss23 | protease, serine, 23 | 5,63E-03 | 1,419 |
| C1rl | complement component 1, r subcomponent-like | 1,03E-02 | 1,267 |
| Serpinb8 | serine (or cysteine) peptidase inhibitor, clade B, member 8 | 4,71E-03 | 1,263 |
| Plat | plasminogen activator, tissue | 1,68E-02 | 1,257 |
| Serpini1 | serine (or cysteine) peptidase inhibitor, clade I, member 1 | 2,26E-04 | 1,251 |
| Prss16 | protease, serine, 16 (thymus) | 1,88E-02 | 1,220 |
| Erap1 | endoplasmic reticulum aminopeptidase 1 | 1,61E-03 | -1,201 |
| Zufsp | zinc finger with UFM1-specific peptidase domain | 2,49E-02 | -1,221 |
| Lmln | leishmanolysin-like (metallopeptidase M8 family) | 9,01E-03 | -1,221 |
| Agbl4 | ATP/GTP binding protein-like 4 | 2,23E-02 | -1,236 |
| Usp11 | ubiquitin specific peptidase 11 | 2,52E-02 | -1,282 |
| Usp29 | ubiquitin specific peptidase 29 | 4,59E-03 | -1,290 |
| Lonrf1 | LON peptidase N-terminal domain and ring finger 1 | 2,40E-03 | -1,380 |
| Cpm | carboxypeptidase M | 3,59E-02 | -1,429 |
| Pcsk6 | proprotein convertase subtilisin/kexin type 6 | 1,18E-03 | -1,454 |
| Thsd4 | thrombospondin, type I, domain containing 4 | 1,30E-03 | -1,484 |
| Reln | reelin | 9,36E-03 | -1,711 |
| **Cytoskeleton and related proteins** | | | |
| Gas2l3 | growth arrest-specific 2 like 3 | 3,47E-03 | 1,902 |
| Kif18b | kinesin family member 18B | 5,65E-03 | 1,624 |
| Frmd5 | FERM domain containing 5 | 4,62E-03 | 1,409 |
| Kif14 | kinesin family member 14 | 7,09E-03 | 1,368 |
| Diap3 | diaphanous homolog 3 (Drosophila) | 4,97E-03 | 1,365 |
| Cd93 | CD93 antigen | 2,92E-03 | 1,307 |
| Synpo2 | synaptopodin 2 | 5,90E-03 | 1,244 |
| Csrp1 | cysteine and glycine-rich protein 1 | 7,65E-03 | 1,228 |
| Pak3 | p21 protein (Cdc42/Rac)-activated kinase 3 | 2,25E-03 | 1,227 |
| Psrc1 | proline/serine-rich coiled-coil 1 | 2,07E-03 | 1,226 |
| Spc25 | SPC25, NDC80 kinetochore complex component, homolog (S. cerevisiae) | 1,40E-02 | 1,225 |
| Smtn | smoothelin | 4,74E-03 | 1,223 |
| Dynll1 | dynein light chain LC8-type 1 | 4,00E-03 | 1,216 |
| Cep72 | centrosomal protein 72 | 1,77E-02 | 1,206 |
| Tmsb10 | thymosin, beta 10 | 4,53E-02 | 1,201 |
| Cldn4 | claudin 4 | 1,39E-02 | -1,200 |
| Pcm1 | pericentriolar material 1 | 1,68E-03 | -1,202 |
| Baiap2 | Brain-Specific Angiogenesis Inhibitor 1-Associated Protein 2 | 1,30E-02 | -1,206 |
| Kifc3 | kinesin family member C3 | 1,41E-03 | -1,216 |
| Trio | triple functional domain (PTPRF interacting) | 1,54E-02 | -1,219 |
| Armc4 | armadillo repeat containing 4 | 1,52E-03 | -1,224 |
| Scin | scinderin | 2,78E-03 | -1,226 |
| Tubb3 | tubulin, beta 3 | 3,36E-02 | -1,233 |
| Tpm3 | tropomyosin 3, gamma | 6,10E-03 | -1,237 |
| Kif3c | kinesin family member 3C | 1,11E-02 | -1,238 |
| Kif5a | kinesin family member 5A | 3,51E-03 | -1,267 |
| Tnik | TRAF2 and NCK interacting kinase | 1,03E-02 | -1,269 |
| Nebl | nebulette | 9,08E-03 | -1,296 |
| Epb4.1l3 | erythrocyte protein band 4.1-like 3 | 6,96E-04 | -1,301 |
| Tmsb15l | thymosin beta 15b like | 1,49E-02 | -1,311 |
| Sntg1 | syntrophin, gamma 1 | 2,40E-03 | -1,333 |
| Syne1 | synaptic nuclear envelope 1 | 3,34E-02 | -1,346 |
| Myo3a | myosin IIIA | 6,78E-03 | -1,359 |
| Syne2 | synaptic nuclear envelope 2 | 2,87E-02 | -1,389 |
| Myo9a | myosin Ixa | 2,98E-03 | -1,527 |
| Edn3 | endothelin 3 | 9,81E-03 | -1,536 |
| Epb4.1l4a | erythrocyte protein band 4.1-like 4a | 1,37E-03 | -1,653 |
| **Channels and transporters** | | | |
| Slc2a6 | solute carrier family 2 (facilitated glucose transporter), member 6 | 2,92E-04 | 2,014 |
| Ttyh1 | tweety homolog 1 (Drosophila) | 2,80E-03 | 1,927 |
| Slc17a9 | solute carrier family 17, member 9 | 3,69E-04 | 1,868 |
| Slco1a5 | solute carrier organic anion transporter family, member 5 | 7,96E-03 | 1,768 |
| Cngb3 | cyclic nucleotide gated channel beta 3 | 6,53E-04 | 1,658 |
| Car4 | carbonic anhydrase 4 | 7,97E-04 | 1,622 |
| Rhd | Rh blood group, D antigen | 9,19E-04 | 1,594 |
| Kcnh1 | potassium voltage-gated channel, subfamily H (eag-related) | 3,69E-04 | 1,529 |
| Kcnip2 | Kv channel-interacting protein 2 | 2,47E-03 | 1,473 |
| Slc39a11 | solute carrier family 39 (metal ion transporter), member 11 | 2,30E-03 | 1,458 |
| Tmem38b | transmembrane protein 38B | 3,89E-02 | 1,457 |
| Slco1a6 | solute carrier organic anion transporter family, member 6 | 1,12E-02 | 1,444 |
| Kcnk10 | potassium channel, subfamily K, member 10 | 1,64E-03 | 1,423 |
| Slc38a10 | solute carrier family 38, member 10 | 4,46E-03 | 1,409 |
| Abcb6 | ATP-binding cassette, sub-family B (MDR/TAP), member 6 | 1,33E-03 | 1,391 |
| Slc1a5 | solute carrier family 1 (neutral amino acid transporter), member 5 | 1,45E-02 | 1,389 |
| Slc35f4 | solute carrier family 35, member F4 | 2,98E-03 | 1,358 |
| Abcc9 | ATP-binding cassette, sub-family C (CFTR/MRP), member 9 | 5,82E-03 | 1,313 |
| Rbp7 | retinol binding protein 7, cellular | 1,22E-02 | 1,303 |
| Slc46a1 | solute carrier family 46, member 1 | 2,56E-03 | 1,291 |
| Abca4 | ATP-binding cassette, sub-family A (ABC1), member 4 | 2,15E-03 | 1,288 |
| Synpr | synaptoporin | 1,33E-03 | 1,274 |
| Trpc4 | transient receptor potential cation channel, subfamily C, | 4,44E-02 | 1,255 |
| Kcnk1 | potassium channel, subfamily K, member 1 | 1,47E-03 | 1,249 |
| Atp13a2 | ATPase type 13A2 | 5,49E-04 | 1,248 |
| Tomm40l | translocase of outer mitochondrial membrane 40 homolog (yeast)-like | 7,38E-04 | 1,242 |
| Slc39a13 | solute carrier family 39 (metal ion transporter), member 13 | 3,45E-03 | 1,239 |
| Slc35b1 | solute carrier family 35, member B1 | 1,33E-03 | 1,236 |
| Slc26a2 | solute carrier family 26 (sulfate transporter), member 2 | 1,29E-02 | 1,227 |
| Slc31a1 | solute carrier family 31, member 1 | 2,34E-03 | 1,221 |
| Slc35c2 | solute carrier family 35, member C2 | 1,66E-03 | 1,219 |
| Slc35a2 | solute carrier family 35 (UDP-galactose transporter), member 35 | 5,96E-04 | 1,218 |
| Tmc6 | transmembrane channel-like gene family 6 | 9,22E-03 | 1,212 |
| Lhfp | lipoma HMGIC fusion partner | 2,22E-02 | 1,209 |
| Accn1 | amiloride-sensitive cation channel 1, neuronal | 3,74E-02 | -1,203 |
| Cacna2d1 | calcium channel, voltage-dependent, alpha2/delta subunit | 1,57E-03 | -1,206 |
| Tmem30b | transmembrane protein 30B | 3,12E-03 | -1,206 |
| Tmem151b | transmembrane protein 151B | 4,19E-03 | -1,227 |
| Tmed8 | transmembrane emp24 domain containing 8 | 4,93E-03 | -1,227 |
| Tmem87b | transmembrane protein 87B | 1,33E-03 | -1,228 |
| Sfxn4 | sideroflexin 4 | 6,47E-03 | -1,236 |
| Unc80 | unc-80 homolog (C. elegans) | 5,93E-03 | -1,239 |
| Cacna1c | calcium channel, voltage-dependent, L type, alpha 1C subunit | 9,90E-03 | -1,243 |
| Cacna1a | calcium channel, voltage-dependent, P/Q type, alpha 1A subunit | 3,44E-03 | -1,251 |
| Prrt1 | proline-rich transmembrane protein 1 | 3,60E-02 | -1,255 |
| Slc7a2 | solute carrier family 7 (cationic amino acid transporter, Y+ System),Member 2 | 1,14E-02 | -1,257 |
| Slc16a9 | solute carrier family 16 (monocarboxylic acid transporters), member 9 | 4,62E-02 | -1,258 |
| Aqp4 | aquaporin 4 | 4,19E-03 | -1,258 |
| Dync2h1 | dynein cytoplasmic 2 heavy chain 1 | 3,83E-02 | -1,283 |
| Slc12a7 | solute carrier family 12, member 7 | 1,18E-02 | -1,286 |
| Tmem146 | transmembrane protein 146 | 5,73E-03 | -1,289 |
| Atp1b2 | ATPase, Na+/K+ transporting, beta 2 polypeptide | 1,13E-03 | -1,294 |
| Trpm5 | transient receptor potential cation channel, subfamily M, member 5 | 6,38E-03 | -1,310 |
| Psd | pleckstrin and Sec7 domain containing | 2,58E-03 | -1,320 |
| Nlgn2 | neuroligin 2 | 5,96E-04 | -1,343 |
| Atp8a2 | ATPase, aminophospholipid transporter-like, class I, type 8A, member 2 | 2,92E-03 | -1,344 |
| Sv2b | synaptic vesicle glycoprotein 2 b | 6,05E-03 | -1,345 |
| Ap1s2 | adaptor-related protein complex 1, sigma 2 subunit | 8,84E-03 | -1,347 |
| Hhatl | hedgehog acyltransferase-like | 3,37E-03 | -1,372 |
| Slc30a1 | solute carrier family 30 (zinc transporter), member 1 | 3,24E-03 | -1,392 |
| Lgi1 | leucine-rich repeat LGI family, member 1 | 2,49E-03 | -1,394 |
| Jph3 | junctophilin 3 | 6,68E-04 | -1,413 |
| Tmem106a | transmembrane protein 106A | 6,26E-04 | -1,425 |
| Tmem132b | transmembrane protein 132B | 5,19E-03 | -1,427 |
| Kcnj12 | potassium inwardly-rectifying channel, subfamily J, member 12 | 6,83E-03 | -1,434 |
| Kcnh8 | potassium voltage-gated channel, subfamily H (eag-related), member 8 | 1,33E-03 | -1,442 |
| Slc29a4 | solute carrier family 29 (nucleoside transporters), member 4 | 1,18E-04 | -1,496 |
| Lyve1 | lymphatic vessel endothelial hyaluronan receptor 1 | 2,56E-02 | -1,541 |
| Kcng3 | potassium voltage-gated channel, subfamily G, member 3 | 1,33E-03 | -1,558 |
| Mt1 | metallothionein 1 | 1,35E-03 | -1,599 |
| Aqp7 | aquaporin 7 | 1,24E-03 | -1,656 |
| **Hormones/growth factors/receptors/neuropeptides and exocytosis** | | | |
| Mc5r | melanocortin 5 receptor | 5,54E-03 | 2,412 |
| Tnfrsf23 | tumor necrosis factor receptor superfamily, member 23 | 2,06E-03 | 1,808 |
| Gabra4 | gamma-aminobutyric acid (GABA) A receptor, subunit alpha | 8,83E-03 | 1,781 |
| Inhba | inhibin beta-A | 1,20E-04 | 1,594 |
| Tgfb3 | transforming growth factor, beta 3 | 3,10E-04 | 1,477 |
| Nucb2 | nucleobindin 2 | 1,23E-04 | 1,433 |
| Aplnr | apelin receptor | 4,81E-02 | 1,410 |
| Egf | epidermal growth factor | 2,65E-04 | 1,395 |
| Gprc5b | G protein-coupled receptor, family C, group 5, member B | 3,80E-03 | 1,379 |
| Oxtr | oxytocin receptor | 2,72E-02 | 1,376 |
| Ffar2 | free fatty acid receptor 2 | 1,22E-02 | 1,369 |
| Rab3d | RAB3D, member RAS oncogene family | 1,15E-04 | 1,355 |
| Olfr558 | olfactory receptor 558 | 1,43E-02 | 1,341 |
| Ldlrad3 | low density lipoprotein receptor class A domain containing 3 | 1,61E-02 | 1,337 |
| Vgf | VGF nerve growth factor inducible | 1,20E-03 | 1,328 |
| Ros1 | Ros1 proto-oncogene | 8,24E-03 | 1,286 |
| Ccbp2 | chemokine binding protein 2 | 1,05E-02 | 1,274 |
| Ednra | endothelin receptor type A | 4,53E-02 | 1,261 |
| Gabrq | gamma-aminobutyric acid (GABA) A receptor, subunit theta | 7,09E-03 | 1,260 |
| Gast | gastrin | 3,22E-03 | 1,229 |
| Fgf1 | fibroblast growth factor 1 | 1,58E-02 | 1,207 |
| Olfr765 | olfactory receptor 765 | 3,20E-02 | 1,203 |
| Fzd7 | frizzled homolog 7 (Drosophila) | 7,66E-03 | -1,205 |
| Spry2 | sprouty homolog 2 (Drosophila) | 4,41E-02 | -1,205 |
| Adora1 | adenosine A1 receptor | 1,34E-03 | -1,208 |
| Glul | glutamate-ammonia ligase (glutamine synthetase) | 1,60E-03 | -1,209 |
| Jmjd1c | jumonji domain containing 1C | 1,36E-02 | -1,210 |
| Stxbp3a | syntaxin binding protein 3A | 1,69E-02 | -1,212 |
| Cnr1 | cannabinoid receptor 1 (brain) | 2,38E-03 | -1,218 |
| Sorcs2 | sortilin-related VPS10 domain containing receptor 2 | 1,35E-02 | -1,223 |
| Avpr1b | arginine vasopressin receptor 1B | 1,37E-02 | -1,227 |
| Itpr3 | inositol 1,4,5-triphosphate receptor 3 | 3,84E-03 | -1,228 |
| Maob | monoamine oxidase B | 2,06E-03 | -1,233 |
| Ephb2 | Eph receptor B2 | 3,47E-03 | -1,236 |
| Olfm2 | olfactomedin 2 | 1,33E-03 | -1,241 |
| Grik5 | glutamate receptor, ionotropic, kainate 5 (gamma 2) | 8,59E-04 | -1,247 |
| Plxna3 | plexin A3 | 2,99E-03 | -1,248 |
| Itpr2 | inositol 1,4,5-triphosphate receptor 2 | 2,28E-02 | -1,259 |
| Hgf | hepatocyte growth factor | 1,95E-02 | -1,270 |
| Rims3 | regulating synaptic membrane exocytosis 3 | 1,57E-02 | -1,278 |
| Fgf14 | fibroblast growth factor 14 | 2,30E-03 | -1,285 |
| Tnfrsf14 | tumor necrosis factor receptor superfamily, member 14 | 9,77E-03 | -1,287 |
| Rara | retinoic acid receptor, alpha | 2,10E-03 | -1,294 |
| Igf1r | insulin-like growth factor I receptor | 5,74E-03 | -1,303 |
| Trpm2 | transient receptor potential cation channel, subfamily M, member 2 | 4,39E-03 | -1,311 |
| Epha7 | Eph receptor A7 | 2,69E-02 | -1,317 |
| Itgb8 | integrin beta 8 | 3,29E-03 | -1,317 |
| Cntfr | ciliary neurotrophic factor receptor | 1,23E-02 | -1,318 |
| Rab3c | RAB3C, member RAS oncogene family | 8,59E-04 | -1,328 |
| Tfrc | transferrin receptor | 2,95E-03 | -1,342 |
| Cd79a | CD79A antigen (immunoglobulin-associated alpha) | 2,80E-03 | -1,345 |
| Cacna1b | calcium channel, voltage-dependent, N type, alpha 1B | 3,90E-03 | -1,347 |
| Gpr137b | G protein-coupled receptor 137B | 2,73E-03 | -1,362 |
| Rnf213 | ring finger protein 213 | 5,65E-03 | -1,393 |
| Gria3 | glutamate receptor, ionotropic, AMPA3 (alpha 3) | 3,99E-03 | -1,404 |
| Gipr | gastric inhibitory polypeptide receptor | 1,03E-02 | -1,404 |
| Chrna4 | cholinergic receptor, nicotinic, alpha polypeptide 4 | 2,17E-03 | -1,407 |
| Grin2c | glutamate receptor, ionotropic, NMDA2C (epsilon 3) | 5,02E-03 | -1,417 |
| Tgfbr3 | transforming growth factor, beta receptor III | 2,49E-02 | -1,430 |
| Itpr1 | inositol 1,4,5-triphosphate receptor 1 | 2,80E-03 | -1,583 |
| Sult1c2 | sulfotransferase family, cytosolic, 1C, member 2 | 2,54E-03 | -1,639 |
| Glra1 | glycine receptor, alpha 1 subunit | 2,40E-03 | -1,644 |
| Stxbp5l | syntaxin binding protein 5-like | 2,26E-04 | -1,844 |
| **Signal transduction** | | | |
| C1qtnf1 | C1q and tumor necrosis factor related protein 1 | 4,65E-03 | 1,731 |
| Cthrc1 | collagen triple helix repeat containing 1 | 1,33E-03 | 1,605 |
| Mctp1 | multiple C2 domains, transmembrane 1 | 2,34E-02 | 1,401 |
| Apcdd1 | adenomatosis polyposis coli down-regulated 1 | 3,00E-03 | 1,373 |
| Shcbp1 | Shc SH2-domain binding protein 1 | 2,36E-02 | 1,360 |
| Cyb561 | cytochrome b-561 | 8,30E-04 | 1,347 |
| Taar4 | trace amine-associated receptor 4 | 1,32E-02 | 1,312 |
| Cdk18 | cyclin-dependent kinase 18 | 1,58E-03 | 1,307 |
| Tspan6 | tetraspanin 6 | 9,06E-03 | 1,304 |
| Ell2 | elongation factor RNA polymerase II 2 | 5,92E-05 | 1,304 |
| Ctnnal1 | catenin (cadherin associated protein), alpha-like 1 | 4,39E-02 | 1,292 |
| Gna14 | guanine nucleotide binding protein, alpha 14 | 1,61E-03 | 1,280 |
| Igfbp3 | insulin-like growth factor binding protein 3 | 9,17E-03 | 1,265 |
| Steap4 | STEAP family member 4 | 2,27E-02 | 1,252 |
| Anxa2 | annexin A2 | 2,39E-02 | 1,238 |
| Diap2 | diaphanous homolog 2 (Drosophila) | 4,03E-03 | -1,200 |
| Gprasp2 | G protein-coupled receptor associated sorting protein 2 | 2,14E-02 | -1,201 |
| Strn | striatin, calmodulin binding protein | 2,44E-02 | -1,205 |
| Slc20a1 | solute carrier family 20, member 1 | 1,63E-02 | -1,205 |
| Bcl9 | B-cell CLL/lymphoma 9 | 2,17E-03 | -1,206 |
| Arhgef9 | CDC42 guanine nucleotide exchange factor (GEF) 9 | 4,57E-02 | -1,208 |
| Sufu | suppressor of fused homolog (Drosophila) | 1,97E-03 | -1,210 |
| Arhgap5 | Rho GTPase activating protein 5 | 2,21E-02 | -1,211 |
| Atxn1 | ataxin 1 | 8,37E-03 | -1,211 |
| Hfe | hemochromatosis | 2,25E-03 | -1,212 |
| Adrbk2 | adrenergic receptor kinase, beta 2 | 2,09E-02 | -1,216 |
| Cbl | Casitas B-lineage lymphoma | 2,36E-03 | -1,216 |
| Appl2 | adaptor protein, phosphotyrosine interaction, PH domain and leucine zipper containing 2 | 1,66E-03 | -1,217 |
| Akt2 | thymoma viral proto-oncogene 2 | 4,04E-03 | -1,219 |
| Dok7 | docking protein 7 | 2,16E-04 | -1,219 |
| Cdon | cell adhesion molecule-related/down-regulated by oncogenes | 5,76E-03 | -1,221 |
| Gabbr1 | gamma-aminobutyric acid (GABA) B receptor, 1 | 1,03E-02 | -1,226 |
| Tspan12 | tetraspanin 12 | 1,62E-02 | -1,238 |
| Axin2 | axin2 | 1,87E-02 | -1,240 |
| Rasa2 | RAS p21 protein activator 2 | 2,06E-03 | -1,240 |
| Gpr179 | G protein-coupled receptor 179 | 4,04E-03 | -1,257 |
| Odz2 | odd Oz/ten-m homolog 2 (Drosophila) | 9,93E-03 | -1,266 |
| Grk5 | G protein-coupled receptor kinase 5 | 2,87E-02 | -1,271 |
| Inpp5e | inositol polyphosphate-5-phosphatase E | 4,98E-03 | -1,276 |
| Lpp | LIM domain containing preferred translocation partner in lipoma | 5,42E-03 | -1,278 |
| Bid | BH3 interacting domain death agonist | 3,24E-03 | -1,278 |
| Sema3e | sema domain, immunoglobulin domain (Ig), short basic domain, secreted, (semaphorin) | 3,94E-03 | -1,288 |
| Atrnl1 | attractin like 1 | 4,57E-03 | -1,290 |
| Mapk15 | mitogen-activated protein kinase 15 | 8,45E-03 | -1,301 |
| Magi2 | membrane associated guanylate kinase, WW and PDZ domain | 1,00E-02 | -1,310 |
| Dpp10 | dipeptidylpeptidase 10 | 1,86E-02 | -1,395 |
| Gpr75 | G protein-coupled receptor 75 | 3,07E-03 | -1,397 |
| Gprc5c | G protein-coupled receptor, family C, group 5, member | 4,09E-03 | -1,407 |
| Gpr98 | G protein-coupled receptor 98 | 2,30E-03 | -1,441 |
| Psd4 | pleckstrin and Sec7 domain containing 4 | 4,47E-03 | -1,441 |
| Baiap3 | BAI1-associated protein 3 | 3,86E-03 | -1,449 |
| **AMPK and mTOR pathways** | | | |
| Akt1s1 | AKT1 substrate 1 (proline-rich) | 3,64E-03 | 1,235 |
| Prkab2 | protein kinase, AMP-activated, beta 2 non-catalytic subunit | 9,86E-03 | -1,216 |
| Rps6ka5 | ribosomal protein S6 kinase, polypeptide 5 | 1,58E-02 | -1,295 |
| Rictor | RPTOR independent companion of MTOR, complex 2 | 1,39E-03 | -1,302 |
| Nptx1 | neuronal pentraxin 1 | 7,52E-03 | -1,359 |
| **Insulin signaling pathway** | | | |
| Shc4 | SHC (Src homology 2 domain containing) family, member 4 | 1,84E-03 | 1,445 |
| Insrr | insulin receptor-related receptor | 2,16E-02 | -1,203 |
| Grb10 | growth factor receptor bound protein 10 | 2,54E-02 | -1,213 |
| Shc2 | SHC (Src homology 2 domain containing) transforming protein 2 | 2,85E-03 | -1,298 |
| Igfbp5 | insulin-like growth factor binding protein 5 | 7,96E-03 | -1,378 |
| Nnat | neuronatin | 6,35E-04 | -1,690 |
| **GTPase activity and regulation** | | | |
| Depdc1a | DEP domain containing 1a | 2,61E-03 | 1,894 |
| Arhgap11a | Rho GTPase activating protein 11A | 5,00E-03 | 1,713 |
| Rasgrf2 | RAS protein-specific guanine nucleotide-releasing factor | 6,53E-04 | 1,511 |
| Arhgap19 | Rho GTPase activating protein 19 | 2,92E-02 | 1,403 |
| Iqgap3 | IQ motif containing GTPase activating protein 3 | 2,80E-02 | 1,391 |
| Arhgef37 | Rho guanine nucleotide exchange factor (GEF) 37 | 1,74E-03 | 1,346 |
| Arhgdig | Rho GDP dissociation inhibitor (GDI) gamma | 1,03E-02 | 1,300 |
| Rapgef5 | Rap guanine nucleotide exchange factor (GEF) 5 | 3,88E-02 | 1,261 |
| Ralgapa2 | Ral GTPase activating protein, alpha subunit 2 (catalytic) | 2,30E-03 | 1,214 |
| Vav3 | vav 3 oncogene | 2,82E-03 | 1,208 |
| Sept3 | septin 3 | 7,70E-03 | -1,221 |
| Rab12 | RAB12, member RAS oncogene family | 8,59E-03 | -1,241 |
| Dennd4c | DENN/MADD domain containing 4C | 6,85E-03 | -1,242 |
| Tbc1d2b | TBC1 domain family, member 2B | 2,31E-03 | -1,252 |
| Rasgef1a | RasGEF domain family, member 1A | 1,84E-02 | -1,254 |
| Gbp6 | guanylate binding protein 6 | 2,58E-02 | -1,267 |
| Rab19 | RAB19, member RAS oncogene family | 1,62E-03 | -1,276 |
| Dock5 | dedicator of cytokinesis 5 | 1,39E-02 | -1,291 |
| Rragb | Ras-related GTP binding B | 4,76E-04 | -1,310 |
| Rit2 | Ras-like without CAAX 2 | 1,81E-02 | -1,330 |
| Dock6 | dedicator of cytokinesis 6 | 4,13E-03 | -1,334 |
| Spata13 | spermatogenesis associated 13 | 3,48E-03 | -1,397 |
| Srgap1 | SLIT-ROBO Rho GTPase activating protein 1 | 3,99E-03 | -1,403 |
| Trib1 | tribbles homolog 1 (Drosophila) | 2,99E-02 | -1,455 |
| Dock10 | dedicator of cytokinesis 10 | 5,42E-03 | -1,461 |
| Rem2 | rad and gem related GTP binding protein 2 | 2,03E-03 | -1,463 |
| Mlph | melanophilin | 1,33E-03 | -1,572 |
| **Kinases/Phosphatases and related proteins** | | | |
| Pbk | PDZ binding kinase | 2,03E-03 | 2,427 |
| Akap6 | A kinase (PRKA) anchor protein 6 | 1,35E-03 | 1,448 |
| Dusp23 | dual specificity phosphatase 23 | 1,22E-03 | 1,350 |
| Ckmt1 | creatine kinase, mitochondrial 1, ubiquitous | 1,41E-03 | 1,220 |
| Stk32a | serine/threonine kinase 32A | 7,36E-03 | 1,213 |
| Dak | dihydroxyacetone kinase 2 homolog (yeast) | 1,39E-03 | 1,213 |
| Ppapdc3 | phosphatidic acid phosphatase type 2 domain containing 3 | 1,83E-02 | 1,200 |
| Prkx | protein kinase, X-linked | 1,62E-03 | -1,201 |
| Ppp2r2b | protein phosphatase 2 (formerly 2A), regulatory subunit | 3,43E-03 | -1,201 |
| Pip5k1c | phosphatidylinositol-4-phosphate 5-kinase, type 1 gamma | 2,26E-04 | -1,202 |
| Akap8 | A kinase (PRKA) anchor protein 8 | 3,09E-03 | -1,203 |
| Prkcb | protein kinase C, beta | 1,39E-03 | -1,207 |
| Prkce | protein kinase C, epsilon | 1,35E-03 | -1,209 |
| Mpp3 | membrane protein, palmitoylated 3 (MAGUK p55 subfamily member 3 | 7,23E-03 | -1,209 |
| Ccnl1 | cyclin L1 | 2,23E-03 | -1,211 |
| Ptpru | protein tyrosine phosphatase, receptor type, U | 4,38E-03 | -1,219 |
| Ankrd44 | ankyrin repeat domain 44 | 2,03E-02 | -1,223 |
| Ulk4 | unc-51-like kinase 4 (C. elegans) | 2,62E-03 | -1,226 |
| Tec | tec protein tyrosine kinase | 8,50E-03 | -1,228 |
| Camk1g | calcium/calmodulin-dependent protein kinase I gamma | 7,53E-03 | -1,229 |
| Map3k2 | mitogen-activated protein kinase kinase kinase 2 | 5,87E-03 | -1,230 |
| Agphd1 | aminoglycoside phosphotransferase domain containing 1 | 1,74E-02 | -1,230 |
| Camk2n1 | calcium/calmodulin-dependent protein kinase II inhibitor | 1,38E-03 | -1,237 |
| Spata5 | spermatogenesis associated 5 | 1,05E-02 | -1,241 |
| Phlpp2 | PH domain and leucine rich repeat protein phosphatase | 6,67E-04 | -1,244 |
| Itpkb | inositol 1,4,5-trisphosphate 3-kinase B | 1,61E-03 | -1,252 |
| Dusp18 | dual specificity phosphatase 18 | 3,36E-03 | -1,259 |
| Inpp4a | inositol polyphosphate-4-phosphatase, type I | 3,83E-03 | -1,279 |
| Rad54l2 | RAD54 like 2 (S. cerevisiae) | 3,42E-03 | -1,290 |
| Cdkl1 | cyclin-dependent kinase-like 1 (CDC2-related kinase) | 3,99E-03 | -1,301 |
| Ppp2r2c | protein phosphatase 2 (formerly 2A), regulatory subunit B, gamma | 2,45E-02 | -1,383 |
| Upp1 | uridine phosphorylase 1 | 1,08E-03 | -1,395 |
| Nek5 | NIMA (never in mitosis gene a)-related expressed kinase 5 | 4,64E-03 | -1,430 |
| Ncs1 | neuronal calcium sensor 1 | 1,61E-03 | -1,443 |
| Mast1 | microtubule associated serine/threonine kinase 1 | 4,75E-04 | -1,667 |
| **Cell-cell signaling** | | | |
| Hmmr | hyaluronan mediated motility receptor (RHAMM) | 2,84E-04 | 2,501 |
| Dll1 | delta-like 1 (Drosophila) | 1,62E-03 | -1,345 |
| **Extracellular matrix/collagen formation** | | | |
| F13a1 | coagulation factor XIII, A1 subunit | 5,92E-05 | 1,765 |
| Sgcd | sarcoglycan, delta (dystrophin-associated glycoprotein) | 6,53E-04 | 1,570 |
| Postn | periostin, osteoblast specific factor | 2,84E-02 | 1,567 |
| Leprel1 | leprecan-like 1 | 1,96E-02 | 1,508 |
| Hapln4 | hyaluronan and proteoglycan link protein 4 | 6,53E-04 | 1,425 |
| Frem2 | Fras1 related extracellular matrix protein 2 | 3,69E-04 | 1,420 |
| Spon2 | spondin 2, extracellular matrix protein | 7,25E-03 | 1,418 |
| Lama5 | laminin, alpha 5 | 2,75E-03 | 1,319 |
| Smoc1 | SPARC related modular calcium binding 1 | 1,84E-02 | 1,288 |
| Lepre1 | leprecan 1 | 5,74E-03 | 1,280 |
| Naglu | alpha-N-acetylglucosaminidase (Sanfilippo disease IIIB) | 3,44E-03 | 1,247 |
| Sparc | secreted acidic cysteine rich glycoprotein | 1,30E-02 | 1,247 |
| Hapln1 | hyaluronan and proteoglycan link protein 1 | 5,95E-03 | 1,223 |
| Plod3 | procollagen-lysine, 2-oxoglutarate 5-dioxygenase 3 | 2,25E-03 | 1,204 |
| Mfap1a | microfibrillar-associated protein 1A | 3,57E-03 | -1,202 |
| Mmp16 | matrix metallopeptidase 16 | 4,19E-03 | -1,205 |
| Vcan | versican | 3,06E-02 | -1,233 |
| Egflam | EGF-like, fibronectin type III and laminin G domains | 5,93E-03 | -1,359 |
| Col6a6 | collagen, type VI, alpha 6 | 5,75E-03 | -1,367 |
| **Chemokines/cytokines/adhesion molecules/innate immunity and related proteins** | | | |
| Il1r2 | interleukin 1 receptor, type II | 1,84E-03 | 1,584 |
| Susd2 | sushi domain containing 2 | 6,26E-04 | 1,582 |
| Troap | trophinin associated protein | 1,74E-03 | 1,511 |
| Tubb6 | tubulin, beta 6 | 1,86E-02 | 1,509 |
| Ccl3 | chemokine (C-C motif) ligand 3 | 2,82E-02 | 1,368 |
| Pcdh18 | protocadherin 18 | 1,85E-02 | 1,348 |
| Cd44 | CD44 antigen | 3,10E-03 | 1,346 |
| Otoa | otoancorin | 6,87E-03 | 1,340 |
| Emilin1 | elastin microfibril interfacer 1 | 4,04E-03 | 1,331 |
| Clec14a | C-type lectin domain family 14, member a | 3,51E-03 | 1,328 |
| Nid2 | nidogen 2 | 2,11E-03 | 1,295 |
| Cd34 | CD34 antigen | 7,25E-03 | 1,291 |
| Fam19a1 | family with sequence similarity 19, member A1 | 1,91E-02 | 1,279 |
| Lama4 | laminin, alpha 4 | 1,89E-02 | 1,262 |
| Igsf5 | immunoglobulin superfamily, member 5 | 1,05E-02 | 1,253 |
| Cntn1 | contactin 1 | 1,66E-03 | 1,245 |
| Sema3f | sema domain, immunoglobulin domain (Ig), short basic domain | 8,73E-03 | 1,218 |
| Rpsa | ribosomal protein SA | 6,71E-04 | 1,209 |
| Vstm2a | V-set and transmembrane domain containing 2A | 2,78E-02 | 1,207 |
| Jam2 | junction adhesion molecule 2 | 7,95E-03 | 1,205 |
| Nrxn1 | neurexin I | 3,99E-03 | -1,202 |
| Il18bp | interleukin 18 binding protein | 4,48E-02 | -1,203 |
| Pcdhb8 | protocadherin beta 8 | 8,23E-03 | -1,203 |
| Sema4f | sema domain, immunoglobulin domain (Ig), TM domain, and s | 1,03E-02 | -1,208 |
| AK129341 | cDNA sequence AK129341 | 1,94E-02 | -1,210 |
| Ppfia2 | protein tyrosine phosphatase, receptor type, f polypeptide (PTPRF), interacting protein (liprin), alpha 2 | 4,37E-02 | -1,211 |
| Plekha2 | pleckstrin homology domain-containing, family A (phosphoinositide binding specific) member 2 | 2,73E-02 | -1,214 |
| Dscam | Down syndrome cell adhesion molecule | 4,06E-02 | -1,218 |
| Igsf9b | immunoglobulin superfamily, member 9B | 9,17E-03 | -1,223 |
| Csf1 | colony stimulating factor 1 (macrophage) | 1,41E-02 | -1,224 |
| Pvrl1 | poliovirus receptor-related 1 | 8,17E-03 | -1,232 |
| Tjp2 | tight junction protein 2 | 1,37E-03 | -1,243 |
| Il18 | interleukin 18 | 1,03E-02 | -1,258 |
| Oas1g | 2'-5' oligoadenylate synthetase 1G | 7,96E-03 | -1,258 |
| Mslnl | mesothelin-like | 2,25E-03 | -1,268 |
| Ddx58 | DEAD (Asp-Glu-Ala-Asp) box polypeptide 58 | 1,35E-03 | -1,268 |
| Pcdh9 | protocadherin 9 | 2,02E-02 | -1,268 |
| Pard3 | par-3 (partitioning defective 3) homolog (C. elegans) | 3,36E-03 | -1,271 |
| Dnm1 | dynamin 1 | 3,44E-03 | -1,283 |
| Tnr | tenascin R | 2,85E-02 | -1,285 |
| Cx3cl1 | chemokine (C-X3-C motif) ligand 1 | 1,57E-03 | -1,287 |
| L1cam | L1 cell adhesion molecule | 1,81E-02 | -1,293 |
| Hpgds | hematopoietic prostaglandin D synthase | 4,52E-02 | -1,295 |
| Ppl | periplakin | 4,68E-02 | -1,308 |
| Dhx58 | DEXH (Asp-Glu-X-His) box polypeptide 58 | 1,42E-02 | -1,311 |
| Ncam2 | neural cell adhesion molecule 2 | 9,29E-03 | -1,312 |
| Pkhd1 | polycystic kidney and hepatic disease 1 | 8,73E-03 | -1,313 |
| Syt1 | synaptotagmin I | 3,64E-02 | -1,321 |
| Cd274 | CD274 antigen | 7,78E-03 | -1,327 |
| Cer1 | cerberus 1 homolog (Xenopus laevis) | 2,47E-03 | -1,369 |
| Igsf21 | immunoglobin superfamily, member 21 | 4,64E-03 | -1,498 |
| Cdh7 | cadherin 7, type 2 | 2,03E-03 | -1,500 |
| Cdh22 | cadherin 22 | 3,69E-04 | -1,531 |
| Pcdh15 | protocadherin 15 | 8,30E-04 | -1,634 |
| Flrt1 | fibronectin leucine rich transmembrane protein 1 | 1,97E-03 | -1,671 |
| **HLA-related** | | | |
| H2-T22 | histocompatibility 2, T region locus 22 | 1,13E-03 | 1,252 |
| **Other functions** | | | |
| Pdyn | prodynorphin | 2,83E-03 | 2,241 |
| Sema3c | sema domain, immunoglobulin domain (Ig), short basic domain | 1,72E-03 | 2,001 |
| S100z | S100 calcium binding protein, zeta | 1,23E-02 | 1,671 |
| Stil | Scl/Tal1 interrupting locus | 4,85E-03 | 1,589 |
| Mfi2 | antigen p97 (melanoma associated) identified by monoclonal antibodies 133.2 and 96.5 | 5,65E-03 | 1,585 |
| Creld2 | cysteine-rich with EGF-like domains 2 | 3,84E-04 | 1,564 |
| Necab2 | N-terminal EF-hand calcium binding protein 2 | 3,69E-04 | 1,474 |
| Car5b | carbonic anhydrase 5b, mitochondrial | 4,74E-03 | 1,425 |
| Csn3 | casein kappa | 4,18E-03 | 1,415 |
| Tmem160 | transmembrane protein 160 | 4,32E-04 | 1,411 |
| Gnat2 | guanine nucleotide binding protein, alpha transducing 2 | 7,97E-03 | 1,387 |
| Nrm | nurim (nuclear envelope membrane protein) | 1,41E-02 | 1,369 |
| Yif1b | Yip1 interacting factor homolog B (S. cerevisiae) | 1,62E-03 | 1,365 |
| Dcx | doublecortin | 7,66E-03 | 1,358 |
| Morc1 | microrchidia 1 | 1,34E-03 | 1,358 |
| Gmfg | glia maturation factor, gamma | 6,57E-03 | 1,349 |
| Entpd1 | ectonucleoside triphosphate diphosphohydrolase 1 | 1,45E-02 | 1,339 |
| St7 | suppression of tumorigenicity 7 | 4,62E-03 | 1,298 |
| Pon3 | paraoxonase 3 | 3,42E-03 | 1,287 |
| Krtap17-1 | keratin associated protein 17-1 | 1,90E-02 | 1,285 |
| Aspn | asporin | 3,32E-02 | 1,282 |
| Nomo1 | nodal modulator 1 | 5,06E-04 | 1,278 |
| Tmem150a | transmembrane protein 150A | 3,84E-03 | 1,254 |
| Prom1 | prominin 1 | 4,57E-02 | 1,254 |
| Lrrtm2 | leucine rich repeat transmembrane neuronal 2 | 2,66E-02 | 1,241 |
| Crygb | crystallin, gamma B | 8,64E-03 | 1,241 |
| Fez1 | fasciculation and elongation protein zeta 1 (zygin I) | 1,72E-03 | 1,235 |
| Atp13a1 | ATPase type 13A1 | 2,92E-03 | 1,234 |
| Nbl1 | neuroblastoma, suppression of tumorigenicity 1 | 4,73E-03 | 1,229 |
| Arfip2 | ADP-ribosylation factor interacting protein 2 | 5,28E-03 | 1,220 |
| Tmem66 | transmembrane protein 66 | 7,42E-04 | 1,218 |
| Sez6l2 | seizure related 6 homolog like 2 | 1,35E-03 | 1,211 |
| Spaca1 | sperm acrosome associated 1 | 3,10E-02 | 1,210 |
| Gap43 | growth associated protein 43 | 3,54E-02 | -1,205 |
| Iqcb1 | IQ calmodulin-binding motif containing 1 | 1,74E-03 | -1,207 |
| Nbeal2 | neurobeachin-like 2 | 2,09E-03 | -1,207 |
| Fam113b | family with sequence similarity 113, member B | 1,66E-02 | -1,211 |
| Zfp62 | zinc finger protein 62 | 1,28E-02 | -1,217 |
| Zcchc11 | zinc finger, CCHC domain containing 11 | 1,62E-03 | -1,218 |
| Phldb2 | pleckstrin homology-like domain, family B, member 2 | 2,02E-02 | -1,218 |
| Vwa5a | von Willebrand factor A domain containing 5A | 2,10E-03 | -1,225 |
| Zswim6 | zinc finger, SWIM domain containing 6 | 1,05E-02 | -1,225 |
| Dzip1 | DAZ interacting protein 1 | 1,71E-02 | -1,232 |
| Tuft1 | tuftelin 1 | 7,92E-03 | -1,238 |
| Edaradd | EDAR (ectodysplasin-A receptor)-associated death domain | 1,70E-02 | -1,241 |
| Tcp11 | t-complex protein 11 | 2,20E-02 | -1,271 |
| Erc2 | ELKS/RAB6-interacting/CAST family member 2 | 4,74E-03 | -1,277 |
| Car15 | carbonic anhydrase 15 | 1,37E-02 | -1,278 |
| AW551984 | expressed sequence AW551984 | 7,23E-03 | -1,281 |
| Mfap2 | microfibrillar-associated protein 2 | 1,53E-02 | -1,281 |
| Nav3 | neuron navigator 3 | 4,36E-03 | -1,325 |
| Zbtb7c | zinc finger and BTB domain containing 7C | 1,61E-03 | -1,362 |
| Mreg | melanoregulin | 6,66E-03 | -1,378 |
| Tekt2 | tektin 2 | 6,26E-04 | -1,381 |
| Dlgap1 | discs, large (Drosophila) homolog-associated protein 1 | 1,62E-03 | -1,390 |
| F3 | coagulation factor III | 1,15E-04 | -1,448 |
| Ifit1 | interferon-induced protein with tetratricopeptide repeats | 1,49E-02 | -1,542 |
| Mt2 | metallothionein 2 | 2,26E-04 | -1,823 |
| **Unknown functions** | | | |
| Tmem179 | transmembrane protein 179 | 1,69E-03 | 1,767 |
| Prr11 | proline rich 11 | 5,59E-03 | 1,728 |
| Svopl | SV2 related protein homolog (rat)-like | 1,62E-03 | 1,617 |
| Slfn9 | schlafen 9 | 1,12E-02 | 1,552 |
| Sdf2l1 | stromal cell-derived factor 2-like 1 | 5,15E-04 | 1,479 |
| Heatr5b | HEAT repeat containing 5B | 2,65E-04 | 1,395 |
| Ccdc85a | coiled-coil domain containing 85A | 3,33E-03 | 1,372 |
| Maged2 | melanoma antigen, family D, 2 | 3,51E-03 | 1,326 |
| Ttc13 | tetratricopeptide repeat domain 13 | 4,98E-04 | 1,312 |
| Wdr90 | WD repeat domain 90 | 8,43E-03 | 1,287 |
| Fam46a | family with sequence similarity 46, member A | 1,39E-02 | 1,285 |
| Gm996 | predicted gene 996 | 1,15E-02 | 1,278 |
| Tmem212 | transmembrane protein 212 | 4,56E-03 | 1,275 |
| Fam55d | family with sequence similarity 55, member D | 2,35E-02 | 1,271 |
| Pter | phosphotriesterase related | 2,32E-02 | 1,262 |
| Gm5465 | predicted gene 5465 | 3,76E-02 | 1,257 |
| Gm5105 | predicted gene 5105 | 1,12E-02 | 1,249 |
| Tmem176a | transmembrane protein 176A | 4,67E-03 | 1,245 |
| Ccdc18 | coiled-coil domain containing 18 | 2,98E-03 | 1,231 |
| Yipf2 | Yip1 domain family, member 2 | 5,28E-03 | 1,218 |
| Tmem218 | transmembrane protein 218 | 4,93E-03 | 1,216 |
| Fam70a | family with sequence similarity 70, member A | 2,81E-02 | 1,215 |
| D17Wsu104e | DNA segment, Chr 17, Wayne State University 104, expression | 1,49E-03 | 1,214 |
| Armcx6 | armadillo repeat containing, X-linked 6 | 4,46E-03 | 1,214 |
| Sval2 | seminal vesicle antigen-like 2 | 2,31E-03 | 1,213 |
| Reep5 | receptor accessory protein 5 | 2,06E-03 | 1,203 |
| Samd9l | sterile alpha motif domain containing 9-like | 2,84E-03 | -1,201 |
| Wdr78 | WD repeat domain 78 | 5,85E-03 | -1,202 |
| Bend7 | BEN domain containing 7 | 8,02E-03 | -1,206 |
| Plekha5 | pleckstrin homology domain containing, family A member 5 | 1,06E-02 | -1,209 |
| Nol4 | nucleolar protein 4 | 5,26E-03 | -1,213 |
| Fhdc1 | FH2 domain containing 1 | 2,26E-04 | -1,213 |
| Gm9958 | predicted gene 9958 | 4,36E-03 | -1,218 |
| Fam159b | family with sequence similarity 159, member B | 6,51E-03 | -1,219 |
| Xkr6 | X Kell blood group precursor related family member 6 | 6,51E-03 | -1,220 |
| Gm10336 | predicted gene 10336 | 3,51E-03 | -1,220 |
| Wdr47 | WD repeat domain 47 | 1,54E-03 | -1,221 |
| Mllt6 | myeloid/lymphoid or mixed-lineage leukemia (Trithorax Homolog,Drosophila); Translocated To, 6 | 1,73E-03 | -1,221 |
| Rnf157 | ring finger protein 157 | 8,17E-03 | -1,222 |
| Zfp229 | zinc finger protein | 4,91E-02 | -1,224 |
| Klhl18 | kelch-like 18 (Drosophila) | 8,11E-03 | -1,225 |
| Zfp568 | zinc finger protein 568 | 4,59E-02 | -1,225 |
| Gm10786 | predicted gene 10786 | 2,64E-02 | -1,239 |
| Vwa5b2 | von Willebrand factor A domain containing 5B2 | 1,11E-02 | -1,240 |
| Dbpht2 | DNA binding protein with his-thr domain | 1,16E-02 | -1,241 |
| Fnbp4 | formin binding protein 4 | 1,30E-03 | -1,241 |
| Ahdc1 | AT hook, DNA binding motif, containing 1 | 7,97E-04 | -1,245 |
| Zcchc2 | zinc finger, CCHC domain containing 2 | 4,92E-03 | -1,246 |
| Kbtbd4 | kelch repeat and BTB (POZ) domain containing 4 | 2,45E-03 | -1,247 |
| Zcwpw2 | zinc finger, CW type with PWWP domain 2 | 3,44E-02 | -1,251 |
| Bod1l | biorientation of chromosomes in cell division 1-like | 1,07E-02 | -1,252 |
| Gm5595 | predicted gene 5595 | 3,39E-02 | -1,255 |
| Nynrin | NYN domain and retroviral integrase containing | 2,39E-02 | -1,258 |
| Ociad2 | OCIA domain containing 2 | 3,29E-02 | -1,259 |
| Gpr137b-ps | G protein-coupled receptor 137B, pseudogene | 9,13E-03 | -1,259 |
| Zfp619 | zinc finger protein 619 | 1,84E-03 | -1,260 |
| Zmym5 | zinc finger, MYM-type 5 | 4,02E-03 | -1,261 |
| Aard | alanine and arginine rich domain containing protein | 2,72E-02 | -1,265 |
| Fam169a | family with sequence similarity 169, member A | 6,54E-03 | -1,270 |
| Klhl32 | kelch-like 32 (Drosophila) | 1,92E-03 | -1,285 |
| Lrrc36 | leucine rich repeat containing 36 | 7,66E-03 | -1,288 |
| BC068157 | cDNA sequence BC068157 | 2,78E-03 | -1,292 |
| Mctp2 | multiple C2 domains, transmembrane 2 | 3,50E-02 | -1,294 |
| Nbeal1 | neurobeachin like 1 | 2,64E-02 | -1,296 |
| Heatr5b | HEAT repeat containing 5B | 1,73E-03 | -1,303 |
| Auts2 | autism susceptibility candidate 2 | 4,99E-03 | -1,313 |
| T2 | brachyury 2 | 1,13E-02 | -1,318 |
| Gm609 | predicted gene 609 | 2,25E-02 | -1,326 |
| Samd14 | sterile alpha motif domain containing 14 | 5,15E-03 | -1,352 |
| Trim12a | tripartite motif-containing 12A | 2,36E-02 | -1,358 |
| Pisd-ps1 | phosphatidylserine decarboxylase, pseudogene 1 | 3,68E-03 | -1,361 |
| Tmem215 | transmembrane protein 215 | 1,35E-03 | -1,374 |
| Gipc2 | GIPC PDZ domain containing family, member 2 | 1,77E-03 | -1,383 |
| Vwa5b1 | von Willebrand factor A domain containing 5B1 | 4,02E-03 | -1,423 |
| Wdr49 | WD repeat domain 49 | 7,24E-03 | -1,476 |
| Lancl3 | LanC lantibiotic synthetase component C-like 3 (bacterial) | 2,46E-03 | -1,495 |
| Lrrc16b | leucine rich repeat containing 16B | 1,44E-03 | -1,596 |
| Fam196a | family with sequence similarity 196, member A | 2,26E-04 | -1,678 |
| Gm11992 | predicted gene 11992 | 1,20E-03 | -1,740 |
